# A Tale of Two Stories: Astrocyte Regulation of Synaptic Depression and Facilitation

**DOI:** 10.1371/journal.pcbi.1002293

**Published:** 2011-12-01

**Authors:** Maurizio De Pittà, Vladislav Volman, Hugues Berry, Eshel Ben-Jacob

**Affiliations:** 1 School of Physics and Astronomy, Tel Aviv University, Ramat Aviv, Israel; 2 Center for Theoretical Biological Physics, University of California, San Diego, La Jolla, California, United States of America; 3 Computational Neurobiology Laboratory, The Salk Institute, La Jolla, California, United States of America; 4 Project-Team Beagle, INRIA Rhône-Alpes, Université de Lyon, LIRIS, UMR5205, Villeurbanne, France; École Normale Supérieure, College de France, CNRS, France

## Abstract

Short-term presynaptic plasticity designates variations of the amplitude of synaptic information transfer whereby the amount of neurotransmitter released upon presynaptic stimulation changes over seconds as a function of the neuronal firing activity. While a consensus has emerged that the resulting decrease (depression) and/or increase (facilitation) of the synapse strength are crucial to neuronal computations, their modes of expression in vivo remain unclear. Recent experimental studies have reported that glial cells, particularly astrocytes in the hippocampus, are able to modulate short-term plasticity but the mechanism of such a modulation is poorly understood. Here, we investigate the characteristics of short-term plasticity modulation by astrocytes using a biophysically realistic computational model. Mean-field analysis of the model, supported by intensive numerical simulations, unravels that astrocytes may mediate counterintuitive effects. Depending on the expressed presynaptic signaling pathways, astrocytes may globally inhibit or potentiate the synapse: the amount of released neurotransmitter in the presence of the astrocyte is transiently smaller or larger than in its absence. But this *global* effect usually coexists with the opposite *local* effect on paired pulses: with release-decreasing astrocytes most paired pulses become facilitated, namely the amount of neurotransmitter released upon spike *i*+1 is larger than that at spike *i*, while paired-pulse depression becomes prominent under release-increasing astrocytes. Moreover, we show that the frequency of astrocytic intracellular Ca^2+^ oscillations controls the effects of the astrocyte on short-term synaptic plasticity. Our model explains several experimental observations yet unsolved, and uncovers astrocytic gliotransmission as a possible transient switch between short-term paired-pulse depression and facilitation. This possibility has deep implications on the processing of neuronal spikes and resulting information transfer at synapses.

## Author Summary

Synaptic plasticity is the capacity of a preexisting connection between two neurons to change in strength as a function of neuronal activity. Because it admittedly underlies learning and memory, the elucidation of its constituting mechanisms is of crucial importance in many aspects of normal and pathological brain function. Short-term presynaptic plasticity refers to changes occurring over short time scales (milliseconds to seconds) that are mediated by frequency-dependent modifications of the amount of neurotransmitter released by presynaptic stimulation. Recent experiments have reported that glial cells, especially hippocampal astrocytes, can modulate short-term plasticity, but the mechanism of such modulation is poorly understood. Here, we explore a plausible form of modulation of short-term plasticity by astrocytes using a biophysically realistic computational model. Our analysis indicates that astrocytes could simultaneously affect synaptic release in two ways. First, they either decrease or increase the overall synaptic release of neurotransmitter. Second, for stimuli that are delivered as pairs within short intervals, they systematically increase or decrease the synaptic response to the second one. Hence, our model suggests that astrocytes could transiently trigger switches between paired-pulse depression and facilitation. This property explains several challenging experimental observations and has a deep impact on our understanding of synaptic information transfer.

## Introduction

Activity-dependent modification of synaptic transmission critically moulds the properties of synaptic information transfer with important implications for computation performed by neuronal circuitry [1]–[4]. Multiple mechanisms could coexist in the same synapse, regulating the strength or the efficacy of synaptic transmission therein in a way that depends on the timing and frequency of prior activity at that same synaptic terminal [5].

One widely studied mechanism responsible for the dependence of synaptic transmission on past activity has been dubbed presynaptic short-term plasticity [6]. Upon repetitive action potential stimulation, the response of a presynaptic terminal – usually assessed as the amount of neurotransmitter molecules released from this latter – will not follow with uniform strength but will be modified in a time- and activity-dependent manner, leading either to facilitation or to depression of synaptic release, or to a mixture of both [2]. Such stimulus-related variations of presynaptic response can span a time scale from few milliseconds to seconds from the stimulus onset [2], [7] and fade away after sufficiently prolonged synaptic inactivity [3], [5].

The ability of a presynaptic terminal to convey stimulus-related information is determined by the probability to release neurotransmitter-containing vesicles upon arrival of action potentials [3], [6]. The release probability depends on the number of vesicles that are ready to be released, i.e. the readily releasable pool, but also on the state of the calcium (Ca^2+^) sensor for the exocytosis of synaptic vesicles [8]. On the mechanistic level, both the finite size and the slow post-stimulus recovery of the readily releasable pool, that is the reintegration of the content of synaptic vesicles, give rise to the phenomenon of short-term presynaptic depression, with the extent of depression being determined by the frequency of prior synaptic stimulation [9]. The dependence of short-term facilitation on the pattern of synaptic activation is likely determined either by the slow removal of free presynaptic residual Ca^2+^ or by the slow unbinding of this latter from the Ca^2+^ sensor [3], although these issues are still debatable [10], [11].

Given the important role assumed by presynaptic short-term plasticity in neural computation [6], [12] and the variety of plastic responses – depression, facilitation or both – exhibited by central synapses [13], [14], it is important to unravel the mechanisms that might govern dynamical transitions between depressing and facilitating synapses. The goal of the present work was to investigate one such candidate mechanism: modulation of presynaptic plasticity by glial cells and astrocytes in particular.

Recent years have witnessed mounting evidence on a possible role of glial cells in the dynamics of neuronal networks [15]. In particular, the specific association of synapses with processes of astrocytes – the main type of glial cells in the hippocampus and the cortex [16]–[18] – together with the discovery of two-way astrocyte-neuron communication [19], [20], suggest an active role of these cells in modulation of synaptic transmission and information processing in the brain [21].

**Figure 1:**
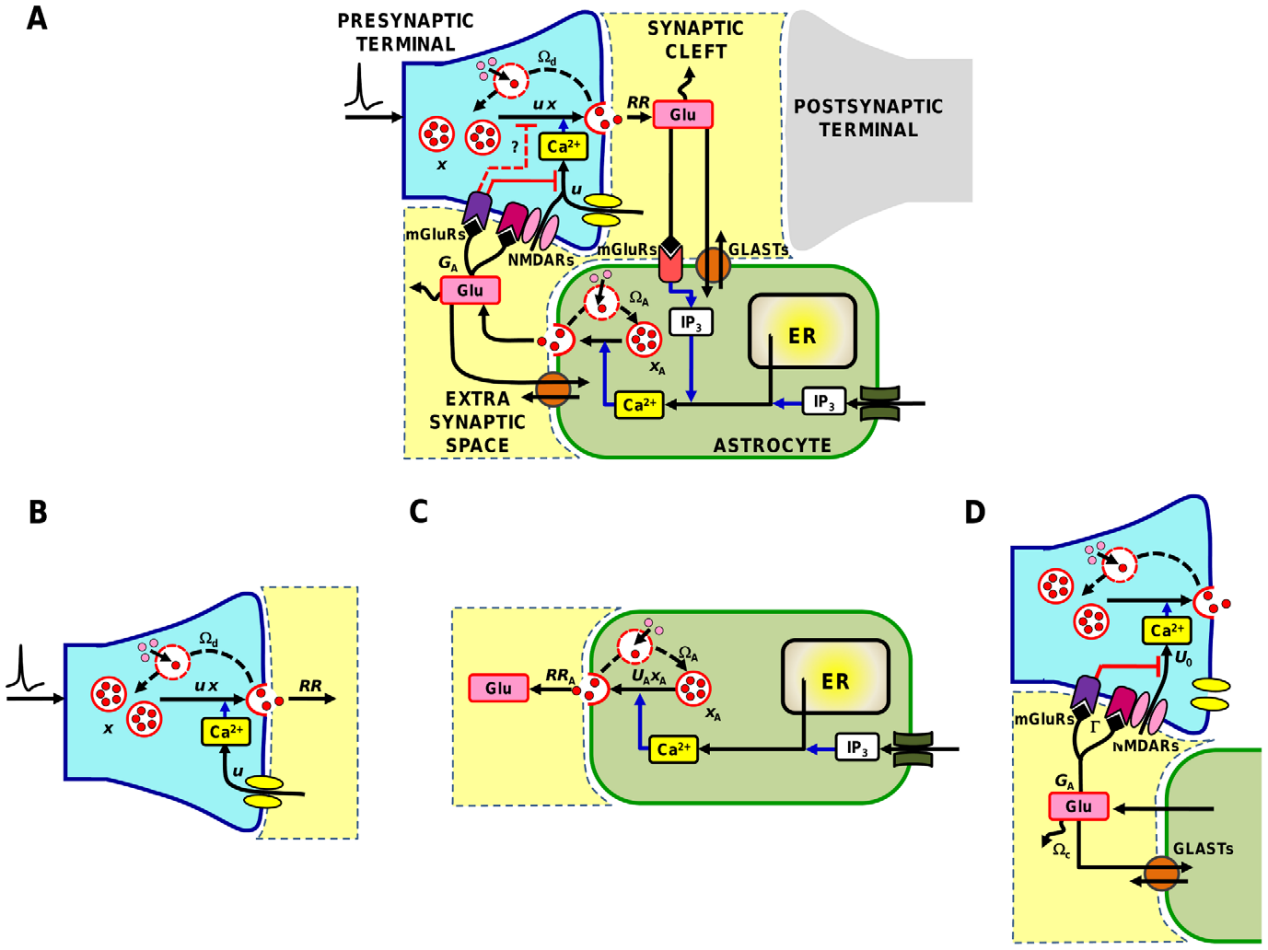
Glutamate-mediated astrocyte regulation of synaptic glutamate release in the hippocampus. (**A**) Glutamate exocytosis from synapses is modulated by the amount of available glutamate (*x*) and the fraction (*u*) of resources used by each presynaptic spike, which reflects presynaptic residual Ca^2+^ concentration. Upon an action potential, an amount *RR* = *ux* of available glutamate is released to produce a postsynaptic response, and it is later reintegrated into the synapse at rate Ω_d_. In the synaptic cleft, released glutamate is cleared by diffusion and uptake by astrocytic glutamate transporters (GLASTs). Part of such glutamate though could also spill out of the cleft and bind to metabotropic glutamate receptors (mGluRs) of neighboring astrocytic processes. The bound receptors then trigger Ca^2+^ release from astrocytic endoplasmic reticulum (ER) stores that is mediated by inositol 1,4,5-trisphospate (IP_3_). Increasing cytosolic Ca^2+^ levels then triggers glutamate release from the astrocyte by a process similar to synaptic glutamate exocytosis. In turn, released astrocytic glutamate diffuses extrasynaptically and binds to pre-terminal receptors (mGluRs or NMDARs) which can modulate further glutamate release from the synapse by different mechanisms, some of which remain to be elucidated. Calcium dynamics by the astrocyte can also be controlled by other mechanisms, including gap junction-mediated intercellular IP_3_ diffusion from neighboring astrocytes or external artificial stimulation. The present study takes into account only this scenario. (**B**–**D**) Building blocks of our model of astrocyte-synapse glutamatergic interactions: (**B**) presynaptic terminal; (**C**) astrocyte; (**D**) glutamate signaling between astrocyte and presynaptic terminal.

Astrocytes could modulate synaptic transmission at nearby synapses by releasing neurotransmitter (or “gliotransmitter”) in a Ca^2+^-dependent fashion [22]. In the hippocampus in particular, several studies have shown that astrocyte-released glutamate modulates short-term plasticity at excitatory synapses either towards depression or facilitation [23]–[25]. This is achieved by activation of presynaptic glutamate receptors [26] (see also Figure 1 for a schematic presentation). Thus, astrocytes are equipped with means to modulate the extent to which presynaptic terminal exhibits short-term depression or facilitation in response to sustained rhythmic stimulation [27].

We devised a biophysically plausible computational model to investigate the characteristics of astrocyte modulation of presynaptic short-term plasticity. Using the model, we were able to identify the parametric regime in which the synaptic response to action potential stimulation can switch from facilitating to depressing and vice versa. This ability to switch synaptic *modus operandi* depended critically on the characteristics of astrocyte-to-synapse signaling. These findings highlight the new potential role played by astrocytes in defining synaptic short-term plasticity and could explain contradicting experimental evidences.

Although based on experimental results in the hippocampus, [28]–[34], our description could also be extended to model other recognized neuron-glia signaling pathways such as GABAergic gliotransmission on interneuron-to-pyramidal cell synapses in the hippocampus [35], glia-mediated ATP release both on hippocampal synapses [36], [37] or in the hypothalamus [38] as well as in the retina [39], and glial modulation of neuromuscular transmission [40]–[42].

## Methods

### The road map of astrocyte regulation of presynaptic short-term plasticity

Regulation of synaptic transmission by astrocyte-released gliotransmitter is supported by an elaborate signaling network schematized in Figure 1. Here, we consider the well-characterized experimental case of glutamate-mediated astrocyte regulation of synaptic transmission in the hippocampus [27], [43]. At excitatory synapses there, astrocytes can respond to synaptically-released glutamate by intracellular Ca^2+^ elevations that in turn, may trigger the release of further glutamate from the astrocytes [22], [44]. This astrocyte-released glutamate (*G*_A_) diffuses in the extrasynaptic space and binds to presynaptic metabotropic glutamate receptors (mGluRs) or NMDA receptors (NMDARs) on neighboring presynaptic terminals [21], [30]. Glutamate activation of these receptors can modulate Ca^2+^ influx into the presynaptic terminal, affecting the release probability of glutamate-containing synaptic vesicles [26]. Thus, glutamate release from the presynaptic terminal is expected to increase the astrocytic intracellular Ca^2+^, eventually leading to glutamate release from that astrocyte. In turn, astrocytic glutamate modulates presynaptic Ca^2+^ and thus affects the amount of glutamate released from that same synapse in response to action potentials that will follow [27].

Astrocyte Ca^2+^ dynamics may also not be modulated by glutamate originating from the very presynaptic terminal that is regulated by the astrocyte, but rather by an exogenous source [45]. This could correspond to the heterosynaptic case whereby two distinct synapses, **A** and **B**, are contacted by processes from the same astrocyte [21]. Glutamate released by the presynaptic terminal of synapse **A** modulates astrocytic Ca^2+^, leading to modulation of glutamate release from the presynaptic terminal of synapse **B**. Alternatively astrocyte Ca^2+^ dynamics could be modulated by intercellular IP_3_ diffusion from neighboring astrocytes through gap junctions [46] or by exogenous stimulation of the astrocyte by different techniques or external stimuli [47], [48], or occur spontaneously [49], [50].

Although both homosynaptic and non-homosynaptic scenarios equally occur physiologically [21], [45], here we focus only on the latter. This approach, which is often adopted in the majority of experiments [30]–[32], [49], presents several advantages. First, it allows us to characterize the effect of astrocytic glutamate on short-term synaptic plasticity in general, that is, independently of the nature of synaptic inputs. Second, it uses Ca^2+^ signals to merely trigger glutamate exocytosis from the astrocyte. Thus we can focus on the timing of glutamate release without considering the complexity of the underlying Ca^2+^ dynamics [48] which can be ultimately modeled by simple stereotypical analytical functions (Text S1, Section I.2). Third, it can be used in the derivation of a mean-field description of synaptic transmission [51], [52] aimed at understanding regulation of short-term synaptic plasticity by a large variety of astrocytic glutamate signals impinging on the synapse, without the need to consider an equally large number of cases.

### Modeling of the astrocyte-to-synapses interaction

#### The Tsodyks-Markram model of a dynamical synapse

To describe the kinetics of a synaptic terminal, we use the model of an activity-dependent synapse first introduced by Tsodyks and Markram [6]. This model assumes that neurotransmitter resources in the presynaptic bouton are limited and only a fraction *x*(*t*) of them is available for release at time *t*. Upon arrival of a presynaptic spike at time *t*_i_, a fraction *u* of these latter is released into the cleft, thus reducing *x* by the amount of “released resources” *RR* = *ux*. As *x*(*t*) recovers to its original value at a rate Ω_d_, the process mimics neurotransmitter depletion and reintegration [9]. The dynamics of *x*(*t*) thus reads:
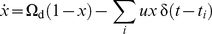
(1)On a par with the classical quantal model of synaptic transmission [53], *x*(*t*) is analogous to the probability of a glutamate-containing vesicle to be available for release at any time *t*, whereas *u* corresponds to the probability of release of a docked vesicle [54]. Accordingly, *u* biophysically correlates with the state of occupancy of the Ca^2+^ sensor of synaptic glutamate exocytosis and its value is incremented following incoming spikes, mimicking Ca^2+^ influx into the presynaptic terminal and its effects on release probability [8]. In particular, at each spike a fraction *U*_0_ of the (1-*u*) vacant states of the sensors is occupied by presynaptic Ca^2+^ ions and later returns to be available at rate Ω_f_. Hence, the dynamics of *u* follows the equation
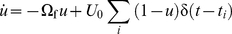
(2)The parameter *U*_0_ coincides with the value of *u* for very low frequencies of stimulation so that it can be regarded as the basal value of synaptic release probability (Text S1, Section I.1).

#### Mechanisms of short-term presynaptic plasticity

Despite its apparent simplicity, the Tsodyks-Markram (TM) model (equations 1–2) can generate surprisingly complex synaptic dynamics including multiple mechanisms of short-term plasticity among which are facilitation and depression. Nonetheless, the occurrence of each mechanism ultimately depends on specific values of synaptic parameters and the rate and the pattern of synaptic activation [55]. The biophysical correlates of different synaptic parameters (e.g., time of recovery from synaptic depression and per-spike usage of synaptic resource) have been extensively documented for central synapses [14], [56], but relatively little effort was done to understand in the TM framework, the nature of transitions between facilitating and depressing synaptic response. Accordingly, we performed thorough theoretical and computational analysis of the TM model.

**Figure 2:**
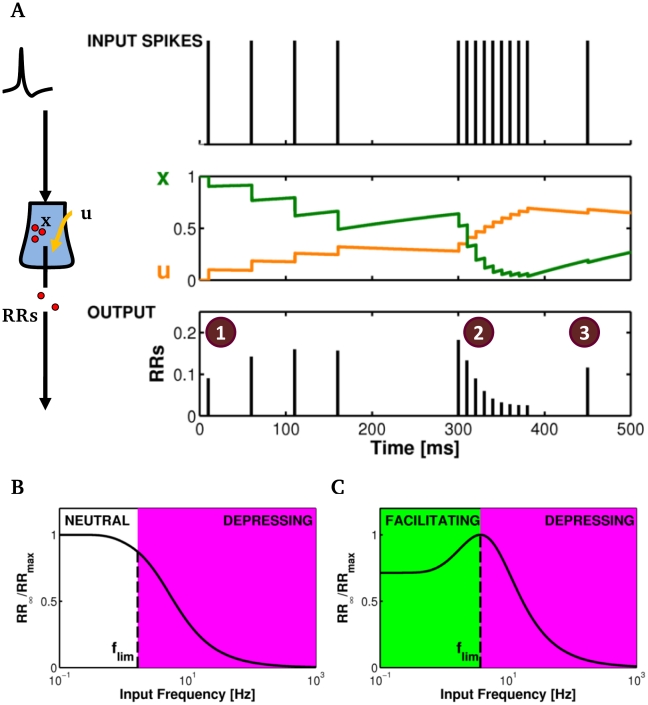
Mechanisms of short-term synaptic plasticity in the TM model. (**A**) A train of presynaptic spikes (*top*) can trigger release of synaptic glutamate resources (RRs, *bottom*) in a variegated fashion by different mechanisms of short-term synaptic plasticity. The interplay between dynamics of synaptic variables *u* and *x* (*middle*) can bring forth (1) facilitation (STP), (2) short-term depression (STD) or (3) recovery from depression. (**B–C**) Mean-field analysis can be deployed to obtain the (normalized) steady-state frequency response of a synapse (*solid line*). (**B**) The latter monotonically decreases for input frequencies larger than the limiting frequency (*f*_lim_, *dashed line*) for a depressing synapse (*red shaded area*). (**C**) In the case of facilitating synapses instead, the frequency response is bimodal hinting occurrence of facilitation for input frequencies below *f*_lim_ (*green shaded area*). Parameters: (**A**) Ω_d_ = 1.67 s^−1^, Ω_f_ = 1.0 s^−1^, *U*_0_ = 0.5; (**B**) Ω_d_ = 2 s^−1^, Ω_f_ = 3.3 s^−1^, *U*_0_ = 0.5, *RR*_max_ 0.5; (**C**) Ω_d_ = 2 s^−1^, Ω_f_ = 2 s^−1^, *U*_0_ = 0.15, *RR*_max_ = 0.21.

In Figure 2A we show a sample response of the TM model to a train of a few input spikes (*top*). The low frequency of the first four spikes largely enables the recovery of available synaptic resource *x* between spikes (*middle*) so that depletion of releasable resources is limited. This process is coupled with a progressive increase of per-spike resource usage *u*, so that the amount of released resources (*RRs*) per spike (*bottom*) increases and short-term potentiation (STP or facilitation) of synaptic response is observed [3]. On the contrary, stimulating the model synapse with a series of high frequency spikes at *t* = 300 ms, results in a rapid increase of *u* but also in a larger depletion of *x*, so that from one spike to the next one, progressively less neurotransmitter is available for release. Consequently, the amount of released resources decreases after each input spike hallmarking the onset of short-term depression (STD) [9]. Finally, a relatively long quiescence before the occurrence of last input spike in the series allows for partial recovery of *x* while *u* hardly changes, which accounts for the increase of resources released by the last spike with respect to immediately preceding ones (compare response in state “3” to the last response in state “2”). Thus, the frequency and the temporal pattern of synaptic stimulation can modulate the synaptic response either transiently facilitating it or transiently depressing it (see also Figure S1).

While the precise pattern of synaptic response is shaped by the timing of input spikes and depends also on initial conditions [13] (Figures 2A, S1, S2; see also Text S1, Section II.1), it is of interest to be able to characterize synaptic release and related plasticity on “average”, namely over different trials of inputs with shared statistics. With this aim, mean-field analysis can be deployed to show that, depending on the basal value *U*_0_, two fundamentally different behaviors can be exhibited by the TM model [51], [57] (Text S1, Section II.3, Figures S3, S7A). When *U*_0_ is larger than the threshold value 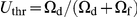, the amount of released resources in the steady state is roughly independent of the input frequency *f*_in_ for low-frequency synaptic stimulation, and decreases only above some cut-off frequency (Figure 2B, *left*). Hence, if *U*_0_>*U*_thr_, the synapse is depressing. On the other hand, when *U*_0_<*U*_thr_ the amount of released resources first increases up to a peak input frequency so that the synapse is facilitating, then it decreases afterwards, marking the onset of depression (Figure 2B, *right*). Therefore, both the cut-off frequency in depressing synapses and the peak frequency in facilitating ones, set an upper limit for the range of input frequencies beyond which STD is observed [6]. For this reason both the cut-off frequency and the peak frequency can be regarded as the “limiting” frequency (*f*_lim_) for the onset of STD for the specific synapse under consideration.

The steady-state frequency response 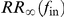 of a synapse can be computed using the mean-field analysis (Text S1, Section II.4) as
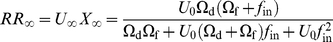
(3)The above equation can then be solved to obtain the expression for *f*_lim_ which reads
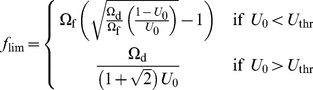
(4)Thus, depending on the value of *U*_0_ with respect to the threshold *U*_thr_, *f*_lim_ is described by different analytical functions with different dependencies on synaptic parameters. Furthermore, while a negative slope of 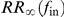 coincides with the onset of depression, a positive slope marks occurrence of facilitation. Accordingly, two conditions are necessary for the occurrence of facilitation in the TM model: (1) that *U*_0_<*U*_thr_, which guarantees the existence of *f*_in_ values for which 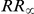 could have either positive or negative slope; and (2) that *f*_in_<*f*_lim_, which assures that the input stimulus effectively falls within the frequency range of positive slope values of 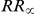.

#### Modeling the action of the astrocytic glutamate on synaptic release

Glutamate release from astrocytes bears several similarities with synaptic exocytosis [27], [44]. Both processes are Ca^2+^-dependent [8], [58]. Furthermore, glutamate is released from astrocytes in quanta consistently with vesicle exocytosis [44], [59]. A vesicular compartment competent for regulated exocytosis, is indeed present in astrocytes [60], [61] and synaptic-like vesicle fusion and recycling is observed in concomitance with astrocytic glutamate exocytosis [62].

Based on such arguments, we assumed that the dynamics of astrocytic glutamate resources could be modeled in a way that is mathematically similar to the TM description of the dynamics of synaptic neurotransmitter resources, although it should be kept in mind that the biological interpretation of the two mechanisms is different [62]. Accordingly, we assumed that a fraction *x*_A_(*t*) of the intracellular astrocytic glutamate is available for release at any time *t*. Any increase of intracellular Ca^2+^ concentration beyond a threshold value *C*_thr_ [59], [63] results in the release of a constant fraction *U*_A_ of *x*_A_ to the extrasynaptic space, and this released gliotransmitter is later reintegrated into the pool of available glutamate resources of the astrocyte at rate Ω_A_ (Text S1, Section I.3).

The effect of the astrocyte-released glutamate (*G*_A_) on the release probability of synaptic neurotransmitter is mediated by the activation of presynaptic glutamate receptors [30], [31], [33]. Several experiments showed that activation of these receptors could modulate the magnitude of Ca^2+^ influx into the presynaptic terminal, thus defining the levels of residual Ca^2+^ therein (reviewed in [26]). Furthermore, activation of presynaptic glutamate receptors can modulate the synaptic response to an action potential via changes in residual synaptic Ca^2+^ [64]. It is important to note that this kind of modulation does not require synaptic activation by action potentials and is observed even in basal conditions [3], [8], [13], likely reflecting changes of the occupancy of Ca^2+^ sensors of exocytosis of synaptic vesicles.

We modeled the effect of astrocytic glutamate on synaptic neurotransmitter release assuming the modulation of synaptic basal release probability *U*_0_ by astrocytic glutamate. In particular, we assumed that *U*_0_ is not a constant (as it is in the original TM model), but rather is a function *U*_0_(Γ) of the fraction Γ of presynaptic glutamate receptors that are activated by astrocyte-derived glutamate. In the absence of quantitative physiological data, we assumed that the function *U*_0_(Γ) is analytic around zero and we considered its first-order expansion, i.e. 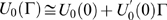. The 0-th order term 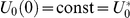 corresponds to the value of *U*_0_ in absence of the astrocyte; hence, in the 0-th order approximation, the model of short-term presynaptic plasticity is just the classical TM model. To express 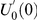, we note that both *U*_0_(Γ) and Γ represent fractions and as such are constrained to the interval [0,1] so that it must be 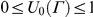. Hence, we define 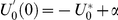 (with 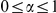), and accordingly (Text S1; Section I.5):
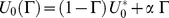
(5)In the above equation, the parameter α lumps in a phenomenological way, all the information related to the activation properties of presynaptic glutamate receptors that mediate the effect of astrocyte on synaptic release (see “The road map of astrocyte regulation of presynaptic short-term plasticity” in “Methods”). Finally, the fraction Γ of presynaptic glutamate receptors that are occupied by astrocyte-released glutamate *G*_A_ is modeled as (Text S1, Section I.5):
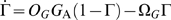
(6)The above parameters *O*_G_ and Ω_G_ are rate constants that biophysically correlate with the rise and decay of the effect of astrocyte glutamate on synaptic neurotransmitter release.

**Figure 3:**
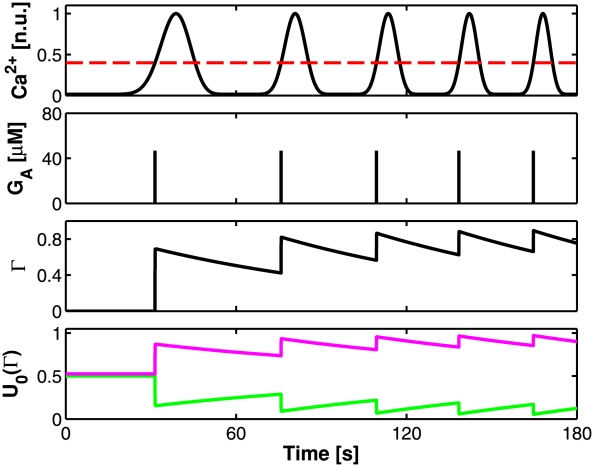
A model of astrocyte modulation of synaptic basal release probability. Astrocyte Ca^2+^ oscillations beyond a threshold value (*C*_thr_, *dashed red line*, *top panel*), trigger transient increases of glutamate (*G*_A_) in the extrasynaptic space surrounding presynaptic receptors. The fraction (Γ) of these latter that bind with astrocytic glutamate modulate synaptic basal release probability (*U*_0_(Γ), *bottom panel*). Depending on the nature of presynaptic receptors, lumped in the “effect” parameter α, astrocytic glutamate can either decrease (for 0<α<*U*_0_ *) or increase for *U*_0_ *<α<1) synaptic release, decreasing or increasing *U*_0_ respectively. Here we show the two border cases of α = 0 (*green line*) and α = 1 (*magenta line*). Parameters: *U*_A_ = 0.3, Ω_G_ = 1 min^−1^. Other parameters as in Table S1.

Figure 3 illustrates how in our model, astrocytic Ca^2+^ oscillations (*top*) modulate synaptic basal release probability (*bottom*) via presynaptic receptors activation (Γ) by astrocyte-released glutamate (*G*_A_) (*middle panels*). The observed saw-shaped increase of Γ is due to the large difference between the rise and decay rates of the astrocyte effect on synaptic release, being *O*_G_ *G*_A_≪Ω_G_ (Text S1, Appendix C). Since in our approximation, *U*_0_ is a linear function of Γ, the time evolution of Γ also determines *U*_0_ according to equation (5). Depending on the value of the effect parameter α, *U*_0_(Γ) can either decrease as low as 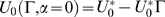 (*bottom panel*, *green line*) or increase as high as 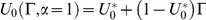 (*bottom panel*, *magenta line*).

## Results

### Astrocyte can either depress or facilitate synaptic neurotransmitter release

**Figure 4:**
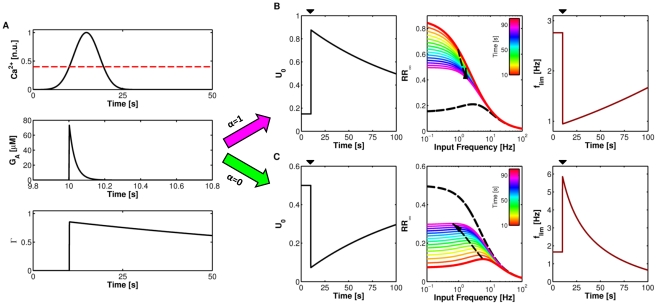
Mechanism of astrocyte regulation of synaptic release. (**A**, *top*) When intracellular Ca^2+^ increases beyond the threshold value for exocytosis (*dashed red line*), the astrocyte releases an amount of glutamate into the extrasynaptic space (*black mark*, *middle*). The resulting fast transient increase of extracellular glutamate activates presynaptic receptors which, depending on the “effect” parameter α in our model, can decrease (**C**, α = 0) or increase (**B**, α = 1) the synaptic basal release probability *U*_0_. Mean-field analysis predicts that steady-state evoked synaptic glutamate release (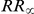) is respectively diminished (**C**, *middle*) or increased (**B**, *middle*) (*colored lines, snapshot color codes* for the time after release of astrocytic glutamate) with respect to the case without astrocytic glutamate (*dashed black line*). (**B,C**, *right*) Equation (4) allows to elucidate how the limiting frequency (*f*_lim_) of the synapse changes under astrocyte signaling. Parameters as in Table S1.

We first studied the effect of astrocytic glutamate release on the transfer properties of our model synaptic terminal. Because the response of a synapse to action potential critically depends on the value of *U*_0_ (equation 3), which in turn could be modulated by astrocytic glutamate binding to presynaptic glutamate receptors (equation 5), we expected that the steady-state frequency response of a synapse (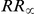) could also be modulated by the astrocyte-synapse signaling. Since both geometry of synaptic bouton and diffusion of glutamate in the extracellular space are beyond the scope of the present work, we implicitly assumed, based on experimental evidence [31], that the release site of astrocytic glutamate apposes targeted presynaptic glutamate receptors. When the intracellular Ca^2+^ in the astrocyte crossed over the threshold of glutamate exocytosis (Figure 4A, *top*, *dashed red line*), the extracellular concentration of glutamate in proximity of presynaptic receptors first increased rapidly and then decayed exponentially at rate Ω_c_, as a result of the concomitant uptake by astrocytic glutamate transporters and diffusion away from the site of exocytosis (Figure 4A, *middle*) (see also Text S1, Section I.4; Figure S5).

For α = 0, equations (5–6) predict that this glutamate peak should lead to a sharp decay of *U*_0,_ followed by a slower recovery phase (Figure 4C, *left*). Using equation (5), we can also predict the resulting dependence of the steady-state synaptic response 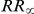 on the input frequency (Figure 4C, *middle*). In the absence of astrocytic glutamate release (*thick dashed black line*), 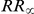 monotonously decreases for increasing input frequency *f*_in_ for the merely depressing synapse considered in this figure. At the release of astrocytic glutamate (Figure 4A, *middle*), the peak of bound presynaptic receptors (Figure 4A, *bottom*) and the resulting sharp drop of *U*_0_ (Figure 4C, *left*, *black mark*) induce a strong decrease of the steady-state amount of released resources at low to intermediate input frequencies (0.1–10 Hz) (Figure 4C, middle, *thick red line*). In addition, the steady-state response loses its monotonicity and displays a peak frequency characteristic of facilitating synapses (see “Mechanisms of short-term presynaptic plasticity” in “Methods”). The 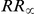 curve then slowly transforms back to its baseline form (*thin colored lines*) and the peak synaptic input frequency appears to progressively shift toward smaller input frequencies (*thick dashed arrow*). Hence, for α = 0, the limiting frequency (equation 4) is predicted to sharply increase following astrocytic glutamate release and then to slowly relax back to smaller values (Figure 4C, *right*).

The exact opposite picture instead describes the scenario of α = 1 (Figure 4B). In this case, the parameter *U*_0_ increases upon astrocytic glutamate release (Figure 4B, *left*) causing a dramatic increase of the steady-state response 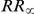 for a range of frequencies within 0.1–10 Hz (Figure 4B, *middle*). Accordingly, the limiting frequency of the synapse dramatically reduces following astrocytic glutamate release, and slowly recovers back to its baseline value (Figure 4B, *right*). Taken together, the above results of the mean-field analysis predict that, depending on the parametric scenario, astrocyte can either transiently decrease, when α = 0, or increase, if α = 1, the release of a model synapse.

**Figure 5:**
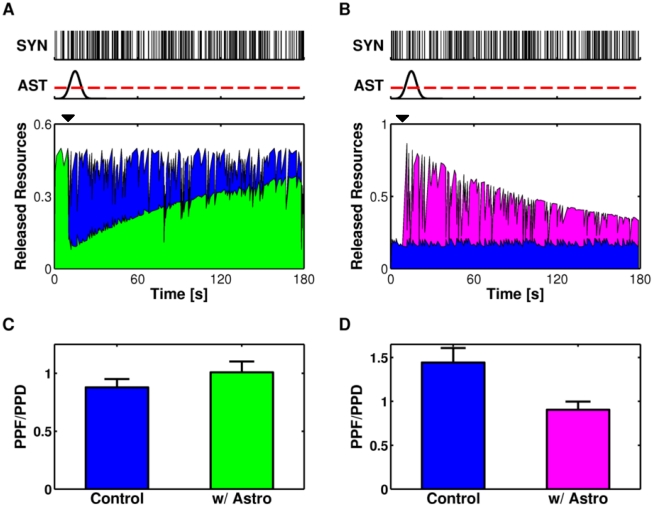
Release-decreasing vs. release-increasing astrocytes. The glutamate resources released by two different model synapses (**A**: depressing; **B**: facilitating) in response to a generic Poisson spike train (SYN, *top*), and without astrocytic signaling, are shown in *blue* (*bottom*). When the astrocyte is included, even a single event of glutamate exocytosis from this latter (onset at *t* = 10 s, *black mark*) triggered by a Ca^2+^ increase therein (AST, *top*), can deeply affect the amount of synaptic resources released by the same input. The nature of the change depends on the nature of presynaptic receptors. (**A**) For α = 0, the effect of the astrocytic glutamate is a global decrease of synaptic release, which fades away slowly from its onset at rate Ω_G_ (*green area*). (**B**) On the other hand, for α = 1 the effect of astrocytic glutamate is to increase synaptic release (*magenta area*). The global change of the amount of released resources is accompanied also by local changes in terms of paired-pulse plasticity. (**C**) For the depressing synapse with release-decreasing astrocyte in (**A**), the ratio between facilitated (PPF) and depressed (PPD) spike pairs, increases in favor of the former. (**D**) The opposite instead occurs for the case of release-increasing astrocyte with the facilitating synapse in (**B**). Bar+Error bar: Mean+Standard Deviation for *n* = 100 Poisson spike trains with the same average rate. Parameters as in Table S1.

To assess the validity of these predictions, we show in Figure 5 the responses of two different model synapses (A: depressing; B: facilitating) to Poisson spike trains delivered at frequency *f*_in_ (Figure 5, *top panels* for specific realizations of such spike trains). To simplify the presentation, we considered the case in which a single Ca^2+^ peak (Figure 5, *middle*) is sufficient to trigger the release of glutamate from the astrocyte. The synaptic response under different scenarios of astrocytic glutamate modulation (A: α = 0; B: α = 1) is then compared to the “Control” scenario obtained for the model synapses without astrocyte. In the case of α = 0 (Figure 5A, *bottom*) the amount of resources released by the model synapse steeply decreased at the onset of glutamate release from the astrocyte (*green area*) and slowly, i.e. tens of seconds, recovered to the levels comparable to those of the control scenario (*blue area*). The opposite effect was observed instead for α = 1 (Figure 5B). The synaptic response in this case was strongly augmented by astrocytic glutamate (*magenta area*) and then slowly decayed back to the levels obtained in control conditions (Figure 5B, *bottom*).

Collectively our mean field analysis (Figure 4) and simulations (Figure 5) suggest that glutamate release by the astrocyte can induce STD or STP of synaptic response to action potentials (Figure S6). Which one between these scenarios occurs depends on the value of the “effect” parameter α that lumps together both the density and the biophysical properties of presynaptic receptors targeted by astrocytic glutamate. These results are consistent with a large body of experimental observations in the hippocampus, where astrocyte-released glutamate could transiently decrease [33] or increase the synaptic response to stimulation [30]–[32], [34], [49].

### Astrocyte-synapse signaling mediates transitions between paired-pulse depression and facilitation at the same synapse

#### Modulation of paired-pulse plasticity by astrocytic glutamate

The analysis presented above disclosed two independent routes to affect synaptic efficacy. (1) On one hand astrocyte-to-synapse signaling could either decrease (α = 0) or increase (α = 1) synaptic release. (2) On the other hand, the synapse itself, in the absence of the astrocyte, could exhibit STP if *U*_0_<*U*_thr_ and *f*_in_<*f*_lim_, or STD otherwise. In principle, these two independent routes give rise to four possible scenarios to modulate the strength of synaptic response. For the sake of clarity, we restrict our attention in the rest of the paper to the intuitively simpler cases of “release-decreasing” astrocytes on otherwise depressing synapses, and of “release-increasing” astrocytes on otherwise facilitating synapses. The complementary cases – i.e. release-decreasing astrocytes on facilitating synapses and release-increasing astrocytes on depressing synapses – are addressed in the Supplementary Online Material (Figures S10 and S11 respectively).

Earlier studies suggested that variations of basal probability of synaptic release due to the activation of presynaptic glutamate receptors, are also expected to change synaptic plasticity as assessed by paired-pulse ratio (PPR) tests [5], [31]. Thus, we set to investigate how astrocytic glutamate modulated synaptic release in pairs of consecutive spikes for the realistic scenario of stochastic input trains such as those in Figure 5 (*top*) [65]. To this aim, we considered the synaptic response to Poisson-distributed spikes, computing the PPR value for each pair of consecutive spikes in the train (Text S1, Section II.2). The results are summarized in the histograms in Figures 5C,D in terms of ratio PPF/PPD of the number of facilitated pulse pairs (i.e. pairs for which PPR>1) over the number of depressed ones (i.e. pairs such that PPR<1), averaged over *n* = 100 Poisson spike trains with the same average frequency.

In the “Control” simulation (i.e. without astrocyte modulation), the depressing synapse of Figure 5A was expectedly characterized by PPF/PPD<1 (Figure 5C, *blue bar*). By contrast, when a release-decreasing astrocyte was incorporated in this synapse, paired-pulse facilitation dominated, with PPF/PPD>1 (*green bar*). The opposite picture was observed instead in the alternative scenario of release-increasing astrocyte modulating the facilitating synapse in Figure 5B. In this latter in fact, while in control simulations the synaptic response was consistently characterized by a ratio PPF/PPD>1 (Figure 5D, *blue bar*), the release-increasing astrocyte shifted instead the balance between facilitated and depressed pairs in favor of these latter thus resulting in PPF/PPD<1 (*magenta bar*).

**Figure 6:**
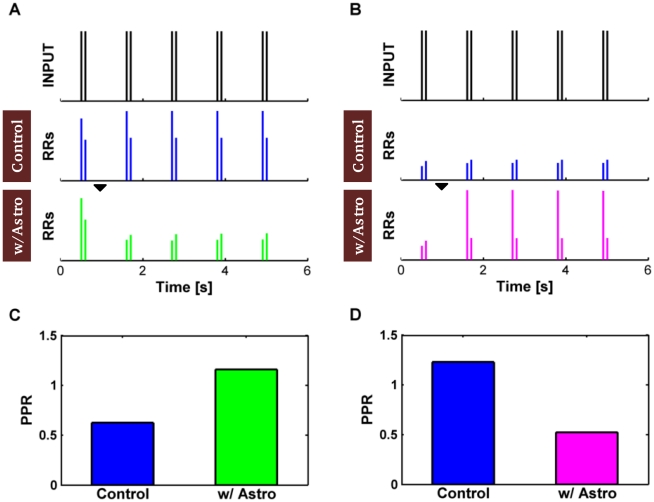
Astrocytic glutamate modifies paired-pulse plasticity. (**A**) A depressing and (**B**) a facilitating synapse are stimulated by a sequence of spike pairs (ISI = 100 ms) at 1 Hz (*top*) and the released resources (RRs) in absence (“Control”, *middle*) vs. in presence of the astrocyte (*bottom*) are monitored. The amount of resources released by spike pairs dramatically changes in presence of glutamate exocytosis from the astrocyte (*black mark* at *t* = 1 s, *bottom*). This behavior evidences a change of paired-pulse plasticity at these synapses which is summarized by the histograms in (**C**,**D**). The release-decreasing astrocyte (i.e. α = 0) on the depressing synapse in (**A**) remarkably increases the average synaptic paired-pulse ratio (PPR) (**C**), while the release-increasing astrocyte (i.e. α = 1) on the facilitating synapse in (**B**) decreases the PPR (**D**), which marks the onset of stronger paired-pulse depression. Parameters as in Table S1.

To rule out the possibility that such increase of PPF (PPD) could have resulted out of the slow increase (decrease) of synaptic release during the recovery (decay) of the effect of release-decreasing (increasing) astrocytic glutamate, model synapses were also stimulated by pairs of spikes (Figure 6). For each spike pair we compared the amount of resources released after the first spike in the pair (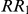) to those released after the second spike in the pair (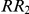). The averaged paired-pulse ratio, defined as 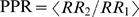, was expected to be larger than 1 for a potentiating synapse, but less than 1 in the case of a depressing synapse (Text S1, Section II.2; Figure S2).

In absence of glutamate release from the astrocyte a depressing synapse responded to a spike pair releasing an amount of resources at the second spike that was *less* than the one at the first spike (Figure 6A, *middle*). Accordingly, the average paired-pulse ratio in this case was PPR<1 (Figure 6C, *blue bar*). In presence of release-decreasing astrocyte however (equation 5-6, α = 0), the response of that same synapse to paired-pulse stimulation changed from depressing to facilitating – that is the amount of resources released upon the second spike in a pair was *larger* than that at the first spike (Figure 6A, *bottom*) –, and the average PPR became larger than 1 (Figure 6C, *green bar*). For the scenario of a release-increasing astrocyte on otherwise facilitating synapse, the exact opposite was observed (Figures 6B,D). Namely, glutamate release from the astrocyte transformed the model synapse from facilitating, i.e. PPR>1 (Figure 6D, *blue bar*) to depressing, i.e. PPR<1 (Figure 6D, *magenta bar*).

Taken together, the above results obtained both from Poisson input spike trains and paired-pulse stimulation hint that astrocytes could modulate short-term paired-pulse synaptic plasticity in a nontrivial way, triggering transitions from PPD to PPF or vice versa.

#### Theoretical explanation of astrocyte-mediated transitions between PPD and PPF

**Figure 7:**
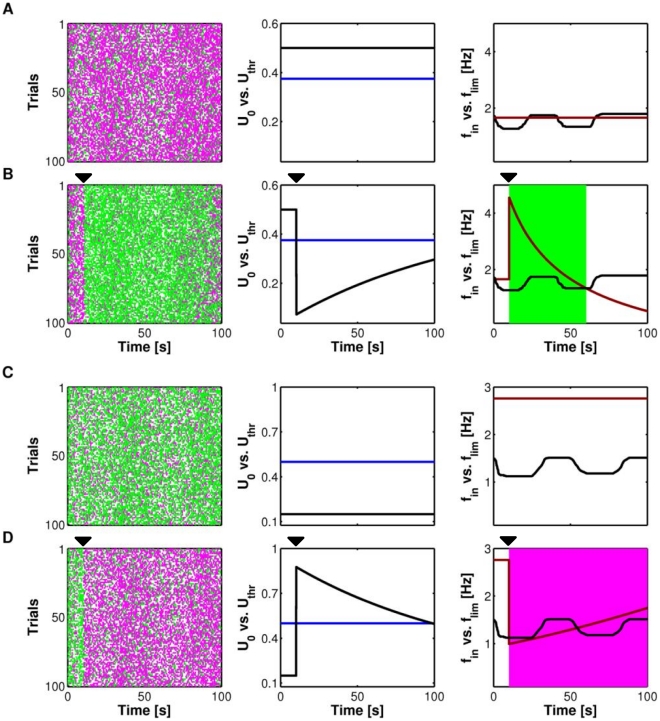
Astrocytic glutamate regulates transitions between facilitation and depression at the same synapse. (**A**,**B**, *left*) Raster plots of a depressing synapse, without (**A**) and in presence of (**B**) a single event of glutamate exocytosis from the astrocyte (onset at the *black mark* at *t* = 1 s, α = 0) for *n* = 100 Poisson spike trains with the same average frequency *f_in_* (*black line* in **A**,**B**, *right*). A *green dot* marks an input spike that released more resources than its preceding one, while a *magenta dot* represents an input spike that released less resources than its previous one. (**A**,**B**, *middle*) The increase of facilitated spike pairs by release-decreasing astrocytic glutamate on the depressing synapse is due to the decrease of synaptic basal release probability *U*_0_ (*black line*) beyond the switching threshold *U*_thr_ (*blue line*) while the limiting frequency (*f*_lim_, *dark red line*) increase above the average input frequency (*f*_in_, *black line*). In such situation in fact, both conditions needed for short-term facilitation are fulfilled (see “Mechanisms of short-term presynaptic plasticity” in “Methods”). (**C, D**) The opposite occurs for a facilitating synapse under the effect of a release-increasing astrocyte (α = 1). In this case in fact, astrocytic glutamate makes *U*_0_ increase beyond *U*_thr_ (**D**, *middle*) while *f*_lim_ switches from above to below *f*_in_, thus marking onset of depression (**D**, *right*). The same results can alternatively be obtained analyzing the slope of the 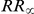 curve (equation 3) for *f*_in_(*t*) (Figure S9). Parameters as in Table S1.

Although the exact order of PPF and PPD for generic input spike trains, such as those in Figure 5, depends on the detail of spike timings of the stimulus, in light of the above observations, the effect of astrocytic glutamate on paired-pulse plasticity is expected to be similar for individual spike trains but all sharing the same statistics. We show this in the raster plots in Figures 7A,B (*left*) for the released resources of a depressing synapse modulated by a release-decreasing astrocyte (i.e. α = 0). Facilitated (*green dots*) vs. depressed spike pairs (*magenta dots*) are displayed for 100 simulated Poisson spike trains sharing the same average frequency. In absence of the astrocyte (“Control”), the majority of pulse pairs is depressed given the depressing nature of the synapse, but as soon as the depressing astrocyte releases a glutamate pulse (Figure 7B, *black mark* at time *t* = 10 s), the number of facilitated pulse pairs becomes the majority, (*green dots* in the raster plot in Figure 7B, *left*). Notably, the alternation of facilitated and depressed pairs is different for each trial, but on average, the number of facilitated pairs increases for all trials right after astrocytic glutamate release.

The occurrence of this scenario can be explained by noting that the effect of the depressing astrocyte complies the two conditions required for PPF (see “Mechanisms of short-term presynaptic plasticity” in “Methods”) namely: (1) that the baseline synaptic release probability *U*_0_ is less than the switching threshold *U*_thr_ and (2) that the frequency of presynaptic spikes is less than the limiting frequency of the synapse. In Figures 7A,B (*middle*) we show how *U*_0_ (*black line*) changes during the stimulus with respect to *U*_thr_ (*blue line*). In the absence of the astrocyte, *U*_0_ is constant and because the synapse is depressing, it is larger than *U*_thr_ (Figure 7A, *middle*). In presence of the astrocyte instead, *U*_0_ changes, rapidly decreasing beyond *U*_thr_ at the onset of glutamate exocytosis from the astrocyte (Figure 7B, *middle*), so that the first condition of facilitation is satisfied.

With regards to the second condition, in Figures 7A,B (*right*) we compare the instantaneous input frequency *f*_in_ (*black line*), i.e. the inverse of the interspike interval averaged over trials, to the limiting frequency *f*_lim_, as given by our mean-field analysis (Figure 4C, *right*, reported as the *dark red line* in Figure 7B, *right*). In control conditions, *f*_lim_ is fixed (because *U*_0_ is constant, see equation 4), and intersections of *f*_lim_ with *f*_in_ do not change synaptic plasticity, because in these conditions *U*_0_>*U*_thr_ anyway. On the other hand, in presence of the astrocyte, *f*_lim_ intersects *f*_in_ at two points (Figure 7.B, *right*): first at the onset of glutamate release and then about 60 seconds later. Hence, at the first intersection of the two curves, the input frequency becomes smaller than the limiting frequency and the second condition for facilitation is also verified. In the raster plot of Figure 7B (*left*), this is marked by a dramatic increase at *t* = 10 s, of the number of green dots that mark facilitated pulse pairs. Conversely, after the second intersection, the input frequency becomes again larger than the limiting frequency, and the return to an essentially depressing regime can be noticed in the associated raster plot by an increasing occurrence of PPD towards the end of the considered time window.

The mirror reasoning also explains why a release-increasing astrocyte increases the chances of observing depressed paired-pulses in a facilitating synapse (Figures 7C,D). Here we start from a control case where both conditions for PPF are satisfied, that is *U*_0_<*U*_thr_ (Figure 7C, *middle*) and *f*_in_<*f*_lim_ (Figure 7C, *right*). Upon glutamate release by the astrocyte this scenario changes instead because *U*_0_ increases below *U*_thr_ thus bringing forth predominant PPD as can be seen in the raster plot in Figure 7D (*left*). Depressed pulse pairs remain predominant also when *U*_0_ recovers back to values below *U*_thr_ towards the end of the considered time window, i.e. at *t* = 100 s. In this case in fact, *f*_lim_ drops to zero by definition (equation 4) (results not shown) becoming less than *f*_in_, which accounts for the predominance of PPD. Identical results can also be obtained by analysis of the slope of the frequency response curve as a function of *f*_in_ (Figure S9).

In summary, our hitherto analysis shows that the effect of the astrocyte can be segregated into two components. First, the astrocyte modulates the overall amount of synaptic resources released after each input spike compared to the case without it. This imposes global decrease or increase of synaptic efficacy in terms of amount of released neurotransmitter. Second, because this effect shifts the location of the limiting frequency of the synapse, the astrocyte can also simultaneously modulate paired-pulse plasticity. Notably, these modulations are in the opposite direction with respect to the global depressing effect. That is, while a release-decreasing astrocyte is predicted to enhance PPF, a release-increasing one could instead reinforce PPD.

### Persistent Ca^2+^ oscillations in astrocytes can regulate presynaptic short-term plasticity

#### Different Ca^2+^ dynamics correspond to different frequencies of glutamate release from the astrocyte

In many cases, astrocytic processes are found to display oscillating Ca^2+^ dynamics. Because the Ca^2+^ threshold for glutamate release is relatively low compared to the amplitude of Ca^2+^ signal [47], [59], [63], one would expect persistent exocytosis of glutamate into the extrasynaptic space [47]. Thus, we proceeded to study the implications of such persistent glutamate release from the astrocyte on modulation of short-term presynaptic plasticity.

Generally speaking, the frequency of astrocytic Ca^2+^ oscillations translates into the frequency at which astrocytic glutamate is released, implying that high rates of Ca^2+^ oscillations would likely lead to stronger and faster depletion of releasable astrocytic glutamate [59]. Conversely, if the frequency of Ca^2+^ oscillations is much smaller than the recovery rate Ω_A_ [44], [66], glutamate can recover in between the oscillation peaks and roughly the same amount of glutamate be released per oscillation. The rate at which the astrocytic glutamate pool is depleted is also likely to depend on the amplitude of Ca^2+^ oscillations, with smaller-amplitude oscillations corresponding to lower probability of exocytosis [59].

**Figure 8:**
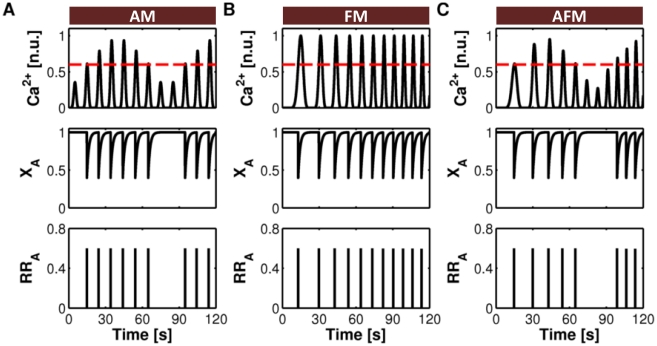
Different Ca^2+^ patterns trigger glutamate release from the astrocyte at different frequencies. Fast reintegration of released glutamate and low-frequency Ca^2+^ oscillations translate (**A**) AM, (**B**) FM and (**C**) AFM Ca^2+^ dynamics (*top*) into different frequency-modulated sequences of glutamate release events (GREs) from the astrocyte (*middle*), all of equal magnitude (*bottom*). Parameters as in Table S1.

The effects of amplitude and frequency of astrocytic Ca^2+^ oscillations on the modulation of synaptic response properties are summarized in Figure 8. We considered three different stereotypical patterns of Ca^2+^ oscillations modulated in time by their amplitude (AM), frequency (FM) or both (AFM) (Text S1, Section I.2; Figure S4). The Ca^2+^ threshold *C*_thr_ for glutamate exocytosis (*dashed red line*) was such that FM oscillations (Figure 8B) always crossed it, each triggering a single glutamate release event [67]. Conversely, AM or AFM oscillations (Figures 8A and 8C) did not always lead to the release of glutamate, as the Ca^2+^ levels did not always reach *C*_thr_. Thus, while the FM oscillations triggered glutamate exocytosis at the same frequency as their own (Figure 8B, *bottom*), the amplitude of AM and AFM oscillations selectively discriminated which oscillations triggered glutamate exocytosis, eventually dictating the frequency of “measured” glutamate release events. Hence, AM oscillations at *constant* frequency (Figure 8A, *top*) would generate sequences of glutamate release events of identical magnitude yet at *variable* frequency (Figure 8A, *bottom*). An implication of this mechanism is that different patterns of Ca^2+^ oscillations could be encoded mainly by the frequency rather than the magnitude of astrocytic glutamate release.

#### Astrocyte regulates transitions between facilitation and depression

**Figure 9:**
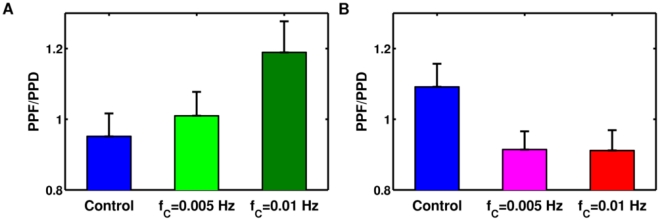
The frequency of astrocytic glutamate release controls the transition between depression and facilitation. Paired-pulse plasticity is considered here for *n* = 100 different Poisson spike trains with the same statistics (as in Figure 7) in presence of persistent glutamate release from the astrocyte. A synapse that in the absence of astrocytic glutamate (“Control”) is otherwise depressing (**A**), can display increasingly more PPF for increasing GRE frequencies (*f*_C_) in presence of release-decreasing astrocyte (α = 0). Conversely, a facilitating synapse (**B**) shows increasing PPD for increasing *f*_C_, under the influence of release-increasing astrocyte (α = 1). Parameters as in Table S1.

Results presented in the previous section lead to the model prediction that if astrocytic Ca^2+^ dynamics is encoded by the frequency *f*_C_ of “measured” glutamate release events (GREs), then this frequency should critically shape the astrocytic modulatory effect on synaptic plasticity. We demonstrate this in Figure 9, where the effect of different GRE frequencies *f*_C_ on facilitated (PPF) vs. depressed (PPD) pulse pairs is shown for *n* = 100 Poisson spike trains with the same average input rate. For a “release-decreasing” astrocyte acting on a depressing synapse, higher rates of GREs lead to stronger facilitation (Figure 9A). By contrast, increasing GRE frequency results in more synaptic depression when a “release-increasing” astrocyte modulates a facilitating synapse (Figure 9B).

We set to determine the effect that the rate *f*_C_ of GREs might have on the basal synaptic release probability *U*_0_. We employed mean-field analysis, assuming the existence of multiple GREs at different frequencies. The steady-state synaptic basal release probability 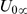 was computed as an explicit function of the GRE frequency *f_C_*, the four rates Ω_A_, Ω_c_, *O*_G_ and Ω_G_ (see Table S1 for an explanation), the total amount of releasable astrocytic glutamate β, and the effect parameter α (the detailed derivation can be found in the Text S1, Section II.5; see also Figures S7B,S8):
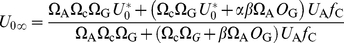
(7)Experimental data [30]–[34] suggests that the rate-limiting step in *U*_0_ dynamics is due to the slow astrocyte modulation of synaptic neurotransmitter release. Thus, we assumed that 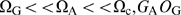 (Table S1). This implies that 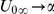 in the high GRE rate regime (for *f*_C_≫Ω_G_). In other words, in the presence of fast astrocytic Ca^2+^ oscillations that would cause persistent release of glutamate at a high rate, the synaptic basal release probability would be stable and would be defined by the nature of presynaptic glutamate receptors.

**Figure 10:**
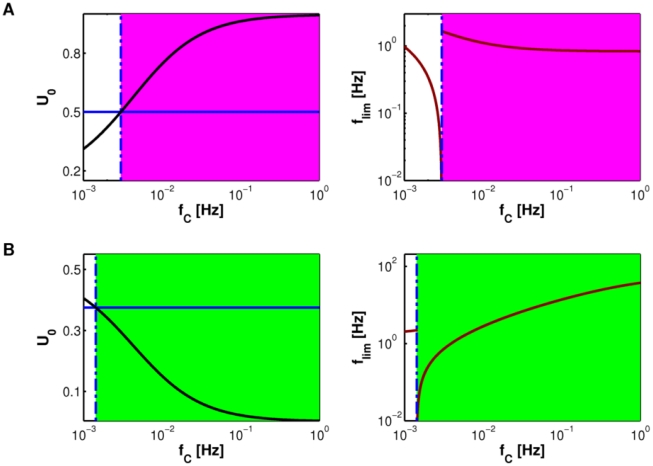
Mean-field analysis of astrocyte regulation of presynaptic short-term plasticity. (**A**,**B**, *left*) With increasing GRE frequencies *f*_C_, *U*_0_ (*solid black line*) crosses the switching threshold *U*_thr_ (*solid blue line*), setting the conditions for a transition either towards predominant depression, for a facilitating synapse with release-increasing astrocyte (**A**, *magenta-shaded area*) or towards predominant facilitation, for a depressing synapse with release-decreasing astrocyte (**B**, *green-shaded area*). (**A**,**B**, *right*) We can also map *f*_lim_ as a function of *f*_C_ (*dark red line*). The crossing of *U*_0_ with *U*_thr_, coincides with a discontinuity of *f*_lim_ (equation 4) and sets a threshold frequency (*f*_thr_) (*dashed blue line*) which marks, for proper input stimuli, the frequency of astrocyte glutamate release that allows switching from facilitation to depression or vice versa. Parameters: (**A**) α = 0; (**B**) α = 1. Other parameters as in Table S1.

This concept is further elucidated in Figures 10. At *f*_C_ = 0.001 Hz, when glutamate release from astrocyte is sporadic, a facilitating synapse without astrocyte expectedly has basal release probability *U*_0_ lower than the threshold value *U*_thr_ at which the synapse switches from facilitating to depressing (Figure 10A, *left*, *blue line*). When a release-increasing astrocyte is added, our prediction suggests that *U*_0_ should *increase* towards α for progressively higher GRE rates. Indeed, above a critical rate *f*_thr_ of GREs, *U*_0_ crosses over from facilitating regime to depressing one (*magenta area* in Figure 10A). For release-decreasing astrocytes acting on depressing synapses, the opposite holds instead, as shown in Figure 10B (*left*). In this latter case however the astrocyte effectively induces PPD-to-PPF transition only if the condition for facilitation on the limiting frequency condition is also satisfied, that is if the input rate of incoming spikes is such that *f*_in_<*f*_lim_ (see “Mechanisms of short-term presynaptic plasticity” in “Methods”).

The synaptic limiting frequency *f*_lim_ for the above-discussed cases as function of the GRE rate is shown in Figures 10A,B (*right*). Taking as an example the case of the release-decreasing astrocyte that modulates the depressing synapse in Figure 10B, we note that for incoming spikes at average frequency *f*_in_ = 1.5 Hz, PPF is effectively expected to prevail on PPD for GRE rates close to 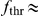
0.0015 Hz (*dashed-dotted blue line*). On the other hand, for higher input frequencies, 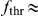
0.0015 Hz is not the effective threshold for the switch between PPD and PPF because for such input rates, the condition required for facilitation that *f*_in_<*f*_lim_ is verified only for *f*_C_>0.0015 Hz. Opposite dependence can be found for the release-increasing astrocyte on the facilitating synapse in Figure 10A.

## Discussion

The character of synaptic information transfer is shaped by several factors [2]. Synaptic strength at any given moment is determined by an earlier “activation history” of that same synapse [3], [5]. Structural and functional organization of presynaptic bouton affects the release and reintegration of neurotransmitter vesicles, ultimately defining the filtering feature (depressing or facilitating) of a synapse in response to spike train stimulation [3], [68]. Existing models of synaptic dynamics assume “fixed plasticity mode”, in which the depression/facilitation properties of a synapse do not change with time. However, in biological synapses, plasticity itself seems to be a dynamic feature; for example, the filtering characteristics of a given synapse is not fixed, but rather can be adjusted by modulation of the initial release probability of docked vesicles [13]. Using a computational modeling approach, we showed here that astrocytes have the potential to modulate the flow of synaptic information via glutamate release pathway. In particular, astrocyte-mediated regulation of synaptic release could greatly increase paired-pulse facilitation (PPF) at otherwise depressing synapses (and thus switch the synapse from depressing to facilitating); conversely, it could reinforce paired-pulse depression (PPD) at otherwise facilitating synapses (therefore switching the synapse from facilitating to depressing). These findings imply that astrocytes could dynamically control the transition between different “plasticity modes”. The present model also lends an explanation to several pieces of experimental data, as we detail below.

In agreement with experimental results [26], [27], our model suggests that the type of presynaptic glutamate receptors targeted by astrocytic glutamate critically determines the type of modulation that takes place. The modulatory action of an astrocyte is lumped in our model into the so-called “effect” parameter α: lower values of α make the action of an astrocyte depressing with respect to the overall synaptic release but also increase paired-pulse facilitation. On the contrary higher α values make the effect of an astrocyte facilitating but at the same time paired-pulse depression is enhanced. Interestingly, some recent experiments on perforant path-granule cell synapses in the hippocampal dentate gyrus, show that facilitation of synaptic release mediated by astrocyte-derived glutamate correlates with a decrease of paired-pulse ratio [31]. Our model provides a natural explanation of these experimental results.

Several lines of experimental evidence suggest that different types of glutamate receptors may be found at the same synaptic bouton [26]. The different types of receptors have different activation properties and hence could be recruited simultaneously or in a complex fashion [30], [41]. Thus it is likely that α could take intermediate values between 0 and 1. In one particular scenario, concurrence of astrocyte-mediated depression and facilitation could also lead these two effects to effectively cancel each other so that no apparent modulation of synaptic release is observed. Interestingly, in some recent studies, the Ca^2+^-dependent release of glutamate from astrocytes was reported not to affect synaptic function [69], [70], thus questioning the vast body of earlier experimental evidence pointing to the contrary. In our model we posit that an apparent lack of astrocytic effect on synaptic function could arise when the “effect” parameter α exactly matches the basal release probability of that presynaptic terminal, that is when α = *U*_0_ * (in which case equation 5 becomes *U*_0_(Γ) = α, meaning that *U*_0_ does not depend on Γ anymore). This scenario would lead to concurrence of astrocyte-mediated depression and facilitation with no net observable effect on synaptic transmission.

Whether *de facto* astrocytes decrease or increase synaptic release likely depends on the specific synapse under consideration and the functional implications that such different modulations could lead to [15], [21], [30]. In the former case for example, enhanced PPF could be not functionally relevant if release of neurotransmitter is strongly reduced by astrocyte glutamate signaling. In such situation the astrocyte would essentially shut down synaptic transmission, hindering the flow of information carried by presynaptic spikes [71]. On the other hand, for astrocyte-induced facilitation, an increase of released neurotransmitter could correspond to a similar increase of transmission probability [72]. However, the associated modulations of paired-pulse plasticity could also account for complex processing of specific – i.e. temporal vs. rate – features of input spike trains [2], [51], [57] that could not otherwise be transmitted by the single synapse, that is without the astrocyte.

In a recent line of experiments on frog neuromuscular junction, it was observed that glial cells could govern the outcome of synaptic plasticity based on their ability to bring forth variegated Ca^2+^ dynamics [40], [41]. In other words, different patterns of Ca^2+^ oscillations in perisynaptic glia were shown to activate different presynaptic receptors and thus to elicit different modulatory effects on neurotransmitter release [41]. This scenario would call for a future modification of our model to include a dependence on astrocytic Ca^2+^ dynamics of the effect parameter α. Nevertheless such observations are generally bolstered by our study. Our model predicts the existence of a threshold frequency for Ca^2+^ oscillations below which PPD (PPF) is predominant with respect to PPF (PPD) and above which the opposite occurs. This supports the idea that different spatiotemporal Ca^2+^ dynamics in astrocytes, possibly due to different cellular properties [73]–[76], could provide specialized feedback to the synapse [40]. Moreover, our model suggests that different types of presynaptic glutamate receptors might not be necessary to trigger different modulations of synaptic transfer properties. The fact that the frequency of Ca^2+^ oscillations could bias synaptic paired-pulse plasticity subtends the notion that not only the nature of receptors, but also the dynamics of their recruitment by gliotransmitter could be a further critical factor in the regulation of synaptic plasticity [27], [41]. This latter could eventually be dictated by the timing and the amount of released glutamate [27], [44] as well as by the ultrastructure of astrocytic process with respect to synaptic terminals which defines the geometry of extracellular space [18], [77] thus controlling the time course of glutamate therein [78].

Remarkably, the threshold frequency of Ca^2+^ oscillations that discriminates between PPD and PPF falls, in our analysis, within the range <2.5 min^−1^ of spontaneous Ca^2+^ oscillations displayed by astrocytes in basal conditions independently of neuronal activity [32], [49], [50], [79], [80], hinting a possible role for these latter in the regulation of synaptic physiology. Spontaneous Ca^2+^ oscillations can indeed trigger astrocytic glutamate release [32], [79]–[82] which could modulate ambient glutamate leading to tonic activation of presynaptic receptors [26], [83]. In this fashion, spontaneous glutamate gliotransmission could constitute a mechanism of regulation of basal synaptic release. Notably, in a line of recent experiments, selective metabolic arrest of astrocytes was observed to depress Schaffer collateral synaptic transmission towards increasing PPF, consistently with a reduction of the basal synaptic release probability as predicted by our analysis [49]. The latter could be also relevant in the homosynaptic case of astrocytic glutamate exocytosis evoked by basal activity of the same presynaptic terminal that it feeds back to [21], [27], [84]. In such conditions, the ensuing influence of astrocytic glutamate on synaptic release correlates with the incoming synaptic stimulus also through Ca^2+^ dynamics in the astrocyte [85], unraveling potentially new mechanisms of modulation of synaptic transmission and plasticity.

Although we focused on regulation of astrocyte at single synapses, our analysis could also apply to synaptic ensembles [51], [54] that could be “contacted” either by the same astrocytic process [30], [32], [80] or by different ones with locally synchronized Ca^2+^ dynamics [81]. In this case, modulation of the release probability by the astrocyte would support the existence of “functional synaptic islands” [86], namely groups of synapses, intermittently established by different spatiotemporal Ca^2+^ dynamics, whose transmission mode and plasticity share common features. The implications that such dynamic astrocyte-synapse interaction might have with regard to information flow in neural circuitry remain to be investigated.

Due to their capacity to modulate the characteristics of synaptic transmission, astrocytes could also alter the temporal order of correlated pre- and postsynaptic spiking that critically dictates spike-timing dependent plasticity (STDP) at the synapse [87]. Thus, astrocyte modulation of short-term plasticity could potentially contribute to ultimately shape persistent modifications of synaptic strength [49], [88], [89] underlying processing, memory formation and storage that provides the exquisite balance, subtlety and smoothness of operation for which nervous systems are held in awe [90]. Future combined physiological and computational studies will determine whether or not this is the case.

## Supporting Information

Figure S1 Conditions for short-term depression and facilitation in the TM model. Short-term plasticity in the TM model is brought forth by inherent synaptic parameters such as Ω_d_, Ω_f_ and *U*_0_, and the frequency of incoming spikes. (**A**,**B**) Depressing synapses are generally characterized by Ω_f_>Ω_d_. In these latter, input spikes at *f*_in_>Ω_d_ (**A**) mark the onset of short-term depression (STD) due to fast depletion of the pool of releasable resources. (**B**) Alternatively, STD can also be observed in high-fidelity synapses, namely synapses characterized by high values of *U*_0_. (**C**,**D**) Facilitating synapses instead are characterized by Ω_f_<Ω_d_ and low release probability. In these latter (**C**), incoming spikes at Ω_f_<*f*_in_<Ω_d_ (or *f*_in_>Ω_d_, Ω_f_) build up presynaptic residual Ca^2+^ levels, increasing the synaptic release, thus evidencing facilitation. (**D**) However, the progressive increase of release probability due to facilitation leads to concomitant growing depletion of the releasable pool and STD eventually takes over facilitation. Legend: input presynaptic spikes are in *black*, released resources (RRs, *blue*) are normalized with respect to their maximum (**A**,**B**: *RR*_max_ = 0.5; **C**: *RR*_max_ = 0.18; **D**: *RR*_max_ = 0.2). Parameters: (**A**) Ω_d_ = 2 s^−1^, Ω_f_ = 1000 s s^−1^, *U*_0_ * = 0.5, *f*_in_ = 50 Hz; (**B**) Ω_d_ = 20 s^−1^, Ω_f_ = 1000 s^−1^, *U*_0_ = 0.5, *f*_in_ = 50 Hz; (**C**) Ω_d_ = 100 s^−1^, Ω_f_ = 1.25 s^−1^, *U*_0_ = 0.05, *f*_in_ = 50 Hz; (**D**) Ω_d_ = 10 s^−1^, Ω_f_ = 1.25 s^−1^, *U*_0_ = 0.1, *f*_in_ = 200 Hz.
(TIF)

Figure S2Paired-pulse plasticity. (**A**, *top*) In a typical paired-pulse stimulus protocol, a pair of spikes with controlled interspike interval is delivered to the synapse and synaptic response to the second spike (*RR*_2_) is compared to synaptic response to the first spike (*RR*_1_) by means of paired-pulse ratio, defined as 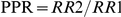. (**A**, *left*) Values of PPR less than 1 mark paired-pulse depression (PPD) as in such conditions *RR*_2_<*RR*_1_. (**A**, *right*) On the contrary, when PPR>1, then *RR*_2_>*RR*_1_ and paired-pulse facilitation (PPF) is observed. The farther the PPR from unity, the stronger the PPD (or PPF). (**A**, *bottom*) The value of PPR critically depends on the interspike interval (ISI) of spike pairs and approaches zero for very long ISIs reflecting the fact that short-term synaptic plasticity is a transient phenomena. (**B**) For a *generic* input spike trains, the PPR between consecutive spikes in a pair is not sufficient to distinguish between PPD and PPF. Depending on the spike timing and on the past synaptic activity in fact, PPR>1 could also result from sufficient reintegration of the pool of releasable resources (Δ*x*>0), despite a decrease of residual Ca^2+^ between the two spikes in a pair (i.e. Δ*u*<0). This situation corresponds to a different form of synaptic plasticity dubbed as “recovery from depression” [13]. (**C–E**) Examples of different short-term plasticity mechanisms listed in the Table (A) displayed by the TM model. Parameters: (**A**, *left*) Ω_d_ = 10 s^−1^, Ω_f_ = 100 s^−1^, *U*_0_ = 0.7, *RR*_max_ = 0.7; (**A**, *right*) Ω_d_ = 100 s^−1^, Ω_f_ = 33 s^−1^, *U*_0_ = 0.05; (**C**, *left*) Ω_d_ = 2 s^−1^, Ω_f_ = 20 s^−1^, *U*_0_ = 0.65; (**C**, *middle*) Ω_d_ = 3.33 s^−1^, Ω_f_ = 10 s^−1^, *U*_0_ = 0.1; (**C**, *right*) Ω_d_ = 4 s^−1^, Ω_f_ = 20 s^−1^, *U*_0_ = 0.1; (**D**, *left*) Ω_d_ = 10 s^−1^, Ω_f_ = 3.33 s^−1^, *U*_0_ = 0.2; (**C**, *right*) Ω_d_ = 5 s^−1^, Ω_f_ = 1 s^−1^, *U*_0_ = 0.16; (**E**) Ω_d_ = 10 s^−1^, Ω_f_ = 5 s^−1^, *U*_0_ = 0.2.(TIF)

Figure S3The switching threshold in the TM model. (**A**, *top*) Mapping of depressing (*red*) and facilitating (*green*) synapses in the parameter plane *U*_0_ vs. *ρ*_Ω_ = Ω_d_/Ω_f_. The two types of synapses are separated by the switching threshold (*black line*) given by 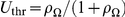 (equation S39). (**A**, *middle*) The limiting frequency *f*_lim_ of a facilitating synapse coincides with the peak frequency of maximal steady-state release of neurotransmitter is maximal (see also Figure 2D). For fixed facilitation rates (i.e. Ω_f_ = const), such limiting frequency increases with *ρ*_Ω_, namely with faster rates (Ω_d_) of reintegration of synaptic resources. In such conditions in fact the larger Ω_d_, the higher the rate of input spikes before the onset of depression. For the same reason, higher *f*_lim_ are also found in correspondence of lower values of synaptic basal release probability *U*_0_ at given *ρ*_Ω_. (**A**, *bottom*) The peak of released resources at the limiting frequency (equation S41) instead increases with *U*_0_ to the detriment of its range of variation (recall in fact, that 0<*RR*_lim_<1). (**B**, *top*) Facilitation regions in the parameter space and mapping therein of *f*_lim_ (equation S40) and (**B**, *bottom*) *RR*_lim_ (equation S41), show strong nonlinear dependence of both quantities on synaptic parameters.(TIF)

Figure S4Astrocyte calcium dynamics. (**A–C**) Superposition of stereotypical functions (*solid line*) on numerically-solved (*black circles*) amplitude and frequency of (**A**) AM-encoding, (**B**) FM-encoding and (**C**) AFM-encoding Ca^2+^ oscillations as obtained from the Li-Rinzel model of Ca^2+^ dynamics [76], [91] (see Text S1, Section I.2). (**D**) Corresponding Ca^2+^ oscillations pertaining to these three modes for the case of an IP_3_ stimulus as in (**E**). Data in (**A–C**, *left* and *middle*) are from [76]. For convenience, only persistent oscillations are considered. The oscillatory range is rescaled between 0 and 1 and amplitude of oscillations is normalized with respect to the maximal Ca^2+^ concentration. Data were fitted by equations (S4, S5, S6) with 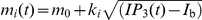 assuming *I*_b_ = 0. (**A**) *C*_0_ = 0.239, *m*_0_ = 0.256, *k* = 0.750; (**B**) *C*_0_ = 0.029, *C*_max_ = 0.939, *m*_0_ = 0.210, *k* = 0.470, *f*_C_ = 0.1 Hz; (**C**) *C*_0_ = 0.079, *m*_0,AM_ = 0.449, *k*_AM_ = 0.611, *m*_0,FM_ = 0.310, *k*_FM_ = 0.480, *f*_C_ = 0.1 Hz. (**D**) *C*_0_ = 0, *m*_0,AM_ = 0, *m*_0,FM_ = 0 Hz, *k*_AM_ = 1, *k*_FM_ = 1, *f*_C_ = 0.1 Hz, *I*_b_ = 0.(TIF)

Figure S5Astrocytic glutamate and presynaptic receptor activation. (**A**) Time course of astrocyte-released glutamate (*G*_A_) in the extrasynaptic space strongly depends on the affinity of astrocytic glutamate transporters for their substrate, i.e. *K*_u_. Several experiments showed that such transporters are not saturated [92] which allows approximating the time course of extrasynaptic glutamate by a single monoexponential decay at rate Ω*_c_* (Text S1, Section I.4). (**B**) Glutamate concentration in the extrasynaptic space around targeted presynaptic receptors depends on average on Ω_A_, that is the rate of reintegration of released glutamate in the astrocyte. On a par with depletion of synaptic resources, for presynaptic spike frequencies larger than Ω_d_, the slower Ω_A_ the stronger the depletion of the astrocytic pool of releasable glutamate for increasing Ca^2+^ oscillations (assumed suprathreshold in this figure). Accordingly, each Ca^2+^ oscillation releases progressively less glutamate. (**C**) The strength of astrocyte modulation of synaptic release depends among the others, on the time course of astrocyte-released glutamate, thus on both Ω_c_ and Ω_A_ rates. Accordingly, at steady-state the average peak of astrocyte effect on synaptic release (i.e. 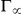, equation S49) increases with the GRE frequency and is stronger for faster rates of reintegration of astrocytic glutamate. (**D**) The strength of astrocyte modulation also depends on past activation of pre-terminal receptors. Thus, it is critically regulated by the decay rate Ω_G_, which biophysically correlates with inherent cellular properties of presynaptic terminal and/or targeted receptors. Experiments show that astrocyte modulation of synaptic release rises fast after glutamate exocytosis, and decays very slowly [30]–[33], at rates that could be comparable to typical frequencies of Ca^2+^ oscillations in the astrocyte [44]. This, in turn, accounts for a progressive saturation of receptors by increasing GRE frequencies for small values of Ω_G_. Parameters: (**A**) *v*_u_ = 60 mMs^−1^, *r*_d_ = 0 s^−1^; (**B**) *G*_v_ = 100 mM, *C*_thr_ = 0, Ω_c_ = 60 s^−1^; (**C–D**) Ω_c_ = 60 s^−1^, *O*_G_ = 1 µM^−1^ s^−1^; n_v_ = 4, G_v_ = 50 mM, *U*_A_ = 0.5, *ρ*_A_ = 6.5·10^−4^, Ω_G_ = 0.67 min^−1^.(EPS)

Figure S6 Regulation of synaptic release by presynaptic glutamate receptors. Simulated bath perfusion by 100 µM glutamate (Glu) for 20 s on a model synapse, can either increase (**A**) or decrease (**B**) synaptic release (RRs) evoked by a generic stimulus (**A,B**, *top*). These results closely reproduce experimental observations [93]–[95] and provide our model with general biophysical consistency. Parameters: (**A**) Ω_d_ = 2 s^−1^, Ω_f_ = 3.3 s^−1^, *U*_0_ * = 0.8, α = 0; (**B**) Ω_d_ = 2 s^−1^, Ω_f_ = 2 s^−1^, *U*_0_ * = 0.15, α = 1; *U*_A_ = 0.4, Ω_G_ = 1 min^−1^, *O*_G_ = 1 µM^−1^ s^−1^. Other parameters as in Table S1.
(EPS)

Figure S7 Range of validity of the mean-field description. (**A**) Product of coefficients of variations for the two synaptic variables *x* and *u* as a function of frequency, allows to estimate the region of validity of the mean-field description (equations S31–S32). In particular, in the domain of the parameter space considered in this study, the error made by averaging exceeds 10% only for a narrow region of such space confined between 4<*f*_in_<6 Hz. (**B**) Analogous considerations hold for averaging of equations (S7, S18, S19). Mapping of the product of coefficients of variations of *x*_A_ and Γ shows that in this case, the error is less than than 7% in the whole parameter space. Parameters: (**A**) Ω_f_ = 2.5 s^−1^; (**B**) *O*_G_ = 1.5 µMs^−1^. Other parameters as in Table S1.
(TIF)

Figure S8Estimation of 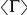. (**A**) Comparison between the exact analytical solution for 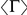 (i.e 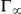 from equation S49; *solid line*) and the approximated one (i.e. 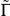 in equation SA6; *dashed line*) used in the computation of the coefficient of variation *c*_Γ_, and (**B**) relative percent error of 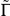 with respect to 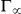. For very low frequencies of Ca^2+^ oscillations (*f*_C_), 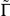 diverges from 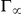 as a result of the assumption of *f_C_*-independent, constant quantal release from the astrocyte, introduced in equation (SA1). While 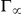 tends to zero as Ca^2+^ oscillations become more and more sporadic because eventually no glutamate is released from the astrocyte, 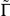 instead does not. This ultimately leads to an incorrect estimation of *c*_Γ_ which is not relevant however within the frequency range of Ca^2+^ oscillations considered in this study.(EPS)

Figure S9Slope analysis. Estimation of the trial-averaged slope 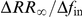 of the synaptic frequency response curve for any value in time of the input frequency *f*_in_ (that is the derivative of 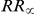 (equation 3) with respect to *f*_in_) allows characterization of any transitions of synaptic plasticity. The method is alternative to that outlined in Figure 7, and relies on the observation that in our model of synaptic plasticity, short-term facilitation is likely to occur whenever 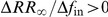 for given input rates, otherwise short-term depression is predominant (see also Text S1, Section II.1). Letters correspond to those in Figure 7, and refer to results of slope analysis for the corresponding cases therein, that is: (**A**) depressing synapse without and (**B**) with release-decreasing astrocyte, and (**C**) facilitating synapse without and (**D**) with release-increasing astrocyte. *Green-shaded* areas denote predominant PPF, *magenta-shaded* areas stand for predominant PPD. Slope values are normalized by their maximum absolute value. Parameters are as in Table S1.(TIF)

Figure S10 Release-decreasing astrocyte on a facilitating synapse. (**A**) Analysis of paired-pulse plasticity in presence of a single glutamate exocytotic event from the astrocyte (same conditions of Figure 5A) shows an increase of the number of facilitated spike pairs (*green bar*) with respect to “Control” simulations (i.e. without astrocyte) (*blue bar*) (bar+error bar: mean+standard deviation). (**B**) Moreover, the larger the frequency of glutamate release from the astrocyte, the stronger the effect. (**C**) Detailed analysis of the different forms of short-term plasticity ongoing within spike pairs – PPF (*dark green*), PPD (*red*) and “recovery from depression” (*black*) – reveals that the increase of the ratio PPF/PPD detected in (**A**–**B**) is mainly imputable to an increase of PPF accompanied by a reduction of recovery from depression. These results confirm the general notion discussed in the text that the effect of a release-decreasing astrocyte coincides with an increase of paired-pulse facilitation (PPF) (see also Figure 7D). Nonetheless, we note that this effect is less pronounced than in a depressing synapse (compare Figures 5A with S9A and Figure 9A with S9B). Data based on *n* = 100 Poisson input spike trains with average rate as in Figure 7C. Data in (**C**) are normalized with respect to their “Control” value: PPF = 197, PPD = 205, recovery = 27. Parameters as in Table S1 with α = 0.
(TIF)

Figure S11 Release-increasing astrocyte on a depressing synapse. (**A**) Analysis of paired-pulse plasticity either for a single (same conditions of Figure 5B) and (**B**) for persistent glutamate exocytosis from the astrocyte, shows an increase of facilitated spike pairs (*magenta*/*red bars*) with respect to the “Control” simulations (i.e. in absence of the astrocyte) (*blue bars*). (**C**) A closer inspection on the nature of ongoing paired-pulse plasticity (PPF: *green*, PPD: *red* and “recovery from depression”: *black*) reveals that such increase is actually caused by an increase of recovery from depression (Control: PPF = 4, PPD = 146, recovery = 135). Bar+Error bar: Mean+Standard deviation. Data based on *n* = 100 Poisson input spike trains with average rate as in Figure 7A. Parameters as in Table S1 with α = 1.
(TIF)

Table S1 Table of parameters of the model of astrocyte-synapse interactions, and corresponding values used in the simulations.
(DOC)

Text S1 Detailed description and derivation of the model of astrocyte-synapse interaction and analytical methods.
(PDF)

## References

[1] Barak O, Tsodyks M (2007). Persistent activity in neural networks with dynamic synapses.. PLoS Comput Biol.

[2] Abbott LF, Regehr WG (2004). Synaptic computation.. Nature.

[3] Zucker RS, Regehr WG (2002). Short-term synaptic plasticity.. Annual Rev Physiol.

[4] Mongillo M, Barak O, Tsodyks M (2008). Synaptic theory of working memory.. Science.

[5] Citri A, Malenka RC (2008). Synaptic plasticity: multiple forms, functions and mechanisms.. Neuropsychopharmacology Rev.

[6] Tsodyks MV, Markram H (1997). The neural code between neocortical pyramidal neurons depends on neurotransmitter release probability.. Proc Natl Acad Sci USA.

[7] Dobrunz LE, Stevens CF (1997). Heterogeneity of release probability, facilitation, and depletion at central synapses.. Neuron.

[8] Südhof TC (2004). The synaptic vesicle cycle.. Annu Rev Neurosci.

[9] Schneggenburger R, Sakaba T, Neher E (2002). Vesicle pools and short-term synaptic depression: lessons form a large synapse.. Trends Neurosci.

[10] Nadkarni S, Bartol TM, Sejnowski TJ, Levine H (2010). Modelling vesicular release at hippocampal synapses.. PLoS Comput Biol.

[11] Sun J, Pang ZP, Qin D, Fahim AT, Adachi R, Südhof TC (2007). A dual-Ca^2+^-sensor model for neurotransmitter release in a central synapse.. Nature.

[12] Abbott LF, Varela JA, Sen K, Nelson SB (1997). Synaptic depression and cortical gain control.. Science.

[13] Dittman JS, Kreitzer AC, Regehr WG (2000). Interplay between facilitation, depression, and residual calcium at three presynaptic terminals.. J Neurosci.

[14] Debanne D, Guerineau NC, Giihwiler BH, Thompson SM (1996). Paired-pulse facilitation and depression at unitary synapses in rat hippocampus: quantal fluctuation affects subsequent release.. J Physiol.

[15] Haydon PG, Carmignoto G (2006). Astrocyte control of synaptic transmission and neurovascular coupling.. Physiol Rev.

[16] Herculano-Houzel S (2009). The human brain in numbers: a linearly scaled-up primate brain.. Frontiers Human Neurosci.

[17] Savchenko VL, McKanna JA, Nikonenko IR, Skibo GG (2000). Microglia and astrocytes in the adult rat brain: comparative immunocytochemical analysis demonstrates the efficacy of lipocortin 1 immunoreactivity.. Neuroscience.

[18] Ventura R, Harris KM (1999). Three-dimensional relationships between hippocampal synapses and astrocytes.. J Neurosci.

[19] Haydon PG (2001). Glia: listening and talking to the synapse.. Nature Rev Neurosci.

[20] Araque A, Parpura V, Sanzgiri RP, Haydon PG (1999). Tripartite synapses: glia, the unacknowledged partner.. Trends Neurosci.

[21] Volterra A, Meldolesi J (2005). Astrocytes, from brain glue to communication elements: the revolution continues.. Nature Rev Neurosci.

[22] Parpura V, Zorec R (2010). Gliotransmission: exocytotic release from astrocytes.. Brain Res Rev.

[23] Perea G, Navarrete M, Araque A (2009). Tripartite synapse: astrocytes process and control synaptic information.. Trends Neurosci.

[24] Agulhon C, Petravicz J, McMullen AB, Sweger EJ, Minton SK (2008). What is the role of astrocyte calcium in neurophysiology?. Neuron.

[25] Barnes BA (2008). The mystery and magic of glia: a perspective on their roles in health and disease.. Neuron.

[26] Pinheiro PS, Mulle C (2008). Presynaptic glutamate receptors: physiological functions and mechanisms of action.. Nature Rev.

[27] Santello M, Volterra A (2009). Synaptic modulation by astrocytes via Ca^2+^-dependent glutamate release.. Neuroscience.

[28] Andersson M, Hanse E (2010). Astrocytes impose postburst depression of release probability at hippocampal glutamate synapses.. J Neurosci.

[29] Andersson M, Blomstrand F, Hanse E (2007). Astrocytes play a critical role in transient heterosynaptic depression in the rat hippocampal CA1 region.. J Physiol.

[30] Perea G, Araque A (2007). Astrocytes potentiate transmitter release at single hippocampal synapses.. Science.

[31] Jourdain P, Bergersen LH, Bhaukaurally K, Bezzi P, Santello M, Domercq M (2007). Glutamate exocytosis from astrocytes controls synaptic strength.. Nature Neurosci.

[32] Fiacco TA, McCarthy KD (2004). Intracellular astrocyte calcium waves *in situ* increase the frequency of spontaneous AMPA receptor currents in CA1 pyramidal neurons.. J Neurosci.

[33] Araque A, Parpura V, Sanzgiri RP, Haydon PG (1998). Glutamate-dependent astrocyte modulation of synaptic transmission between cultured hippocampal neurons.. Eur J Neurosci.

[34] Araque A, Sanzgiri RP, Parpura V, Haydon PG (1998). Calcium elevation in astrocytes causes an NMDA receptor-dependent increase in the frequency of miniature synaptic currents in cultured hippocampal neurons.. J Neurosci.

[35] Kang J, Jiang L, Goldman SA, Nedergaard M (1998). Astrocyte-mediated potentiation of inhibitory synaptic transmission.. Nat Neurosci.

[36] Serrano A, Haddjeri N, Lacaille J, Robitaille R (2006). GABAergic network activation of glial cells underlies heterosynaptic depression.. J Neurosci.

[37] Pascual O, Casper KB, Kubera C, Zhang J, Revilla-Sanchez R (2005). Astrocytic purinergic signaling coordinates synaptic networks.. Science.

[38] Gordon GRJ, Iremonger KJ, Kantevari S, Ellis-Davies GCR, MacVicar BA (2009). Astrocyte-mediated distributed plasticity at hypothalamic glutamate synapses.. Neuron.

[39] Newman EA (2003). Glial cell inhibition of neurons by release of ATP.. J Neurosci.

[40] Rousse I, St-Amour A, Darabid H, Robitaille R (2010). Synapse-glia interactions are governed by synaptic and intrinsic glial properties.. Neuroscience.

[41] Todd KJ, Darabid H, Robitaille R (2010). Perisynaptic glia discriminate patterns of motor nerve activity and influence plasticity at the neuromuscular junction.. J Neurosci.

[42] Robinson R (1998). Modulation of synaptic efficacy and synaptic depression by glial cells at the frog neuromuscular junction.. Neuron.

[43] Fellin T (2009). Communication between neurons and astrocytes: relevance to the modulation of synaptic and network activity.. J Neurochem.

[44] Montana V, Malarkey EB, Verderio C, Matteoli M, Parpura V (2006). Vesicular transmitter release from astrocytes.. Glia.

[45] Giaume C, Koulakoff A, Roux L, Holcman D, Rouach N (2010). Astroglial networks: a step further in neuroglial and gliovascular interactions.. Nat Rev Neurosci.

[46] Kang N, Xu J, Xu Q, Nedergaard M, Kang J (2005). Astrocytic glutamate release-induced transient depolarization and epileptiform discharges in hippocampal CA1 pyramidal neurons.. J Neurophysiol.

[47] Shigetomi E, Kracun S, Sovfroniew MS, Khakh BS (2010). A genetically targeted optical sensor to monitor calcium signals in astrocyte processes.. Nature Neurosci.

[48] Nimmerjahn A (2009). Astrocytes going live: advances and challenges.. J Physiol.

[49] Bonansco C, Couve A, Perea G, Ferradas CA, Roncagliolo M (2011). Glutamate released spontaneously from astrocytes sets the threshold for synaptic plasticity.. Eur J Neurosci.

[50] Nett WJ, Oloff SH, McCarthy KD (2002). Hippocampal astrocytes in situ exhibit calcium oscillations that occur independent of neuronal activity.. J Neurophysiol.

[51] Tsodyks M, Pawelzik K, Markram H (1998). Neural networks with dynamic synapses.. Neural Comput.

[52] Amit DJ, Tsodyks MV (1991). Quantitative study of attractor neural network retrieving at low spike rates: I. Substrate-spikes, rates and neuronal gain.. Network.

[53] Del Castillo J, Katz B (1954). Quantal components of the end-plate potential.. J Physiol.

[54] Fuhrmann G, Segev I, Markram H, Tsodyks M (2002). Coding of temporal information by activity-dependent synapses.. J Neurophysiol.

[55] Markram H, Pikus D, Gupta A, Tsodyks M (1998). Potential for multiple mechanisms, phenomena and algorithms for synaptic plasticity at single synapses.. Neuropharmacology.

[56] Dittman JS, Regehr WG (1998). Calcium dependence and recovery kinetics of presynaptic depression at the climbing fiber to Purkinje cell synapse.. J Neurosci.

[57] Tsodyks M, Chow CC, Gutkin B, Hansel D, Meunier C, Dalibard J (2005). Activity-dependent transmission in neocortical synapses.. Methods and Models in Neurophysics.

[58] Lee W, Parpura V, Bean A (2007). Exocytotic release of glutamate from astrocytes: comparison to neurons.. Protein trafficking in neurons.

[59] Pasti L, Zonta M, Pozzan T, Vicini S, Carmignoto G (2001). Cytosolic calcium oscillations in astrocytes may regulate exocytotic release of glutamate.. J Neurosci.

[60] Bergersen LH, Gundersen V (2009). Morphological evidence for vesicular glutamate release from astrocytes.. Neuroscience.

[61] Zhang Q, Fukuda M, Van Bockstaele E, Pascual O, Haydon PG (2004). Synaptotagmin IV regulates glial glutamate release.. Proc Natl Acad Sci USA.

[62] Marchaland J, Calì C, Voglmaier SM, Li H, Regazzi R (2008). Fast subplasma membrane Ca^2+^ transients control exo-endocytosis of synaptic-like microvesicles in astrocytes.. J Neurosci.

[63] Parpura V, Haydon PG (2000). Physiological astrocytic calcium levels stimulate glutamate release to modulate adjacent neurons.. Proc Natl Acad Sci USA.

[64] Hori T, Takahashi T (2009). Mechanisms underlying short-term modulation of transmitter release by presynaptic depolarization.. J Physiol.

[65] Softky W, Koch C (1993). The highly irregular firing pattern of cortical cells is inconsistent with temporal integration of random EPSPs.. J Neurosci.

[66] Bowser DN, Khakh BS (2007). Two forms of single-vesicle astrocyte exocytosis imaged with total internal reflection fluorescence microscopy.. Proc Natl Acad Sci USA.

[67] Pasti L, Volterra A, Pozzan T, Carmignoto G (1997). Intracellular calcium oscillations in astrocytes: a highly plastic, bidirectional form of communication between neurons and astrocytes *in situ*.. J Neurosci.

[68] Markram H, Wang Y, Tsodyks M (1998). Differential signaling via the same axon of neocortical pyramidal neurons.. Proc Natl Acad Sci USA.

[69] Agulhon C, Fiacco TA, McCarthy KD (2010). Hippocampal short- and long-term plasticity are not modulated by astrocyte Ca^2+^ signalling.. Science.

[70] Fiacco TA, Agulhon C, Taves SR, Petravicz J, Casper KB (2007). Selective stimulation of astrocyte calcium in situ does not affect neuronal excitatory synaptic activity.. Neuron.

[71] Volman V, Ben-Jacob E, Levine H (2007). The astrocyte as a gatekeeper of synaptic information transfer.. Neur Comput.

[72] Nadkarni S, Jung P, Levine H (2008). Astrocytes optimize the synaptic transmission of information.. PLoS Comput Biol.

[73] Goldberg M, De Pittà M, Volman V, Berry H, Ben-Jacob E (2010). Nonlinear gap junctions enable long-distance propagation of pulsating calcium waves in astrocyte networks.. PLoS Comput Biol.

[74] De Pittà M, Goldberg M, Volman V, Berry H, Ben-Jacob E (2009). Glutamate-dependent intracellular calcium and IP_3_ oscillating and pulsating dynamics in astrocytes.. J Biol Phys.

[75] De Pittà M, Volman V, Levine H, Ben-Jacob E (2009). Multimodal encoding in a simplified model of intracellular calcium signaling.. Cogn Proc.

[76] De Pittà M, Volman V, Levine H, Pioggia G, De Rossi D (2008). Coexistence of amplitude and frequency modulations in intracellular calcium dynamics.. Phys Rev E.

[77] Haber M, Zhou L, Murai KK (2006). Cooperative astrocyte and dendritic spine dynamics at hippocampal excitatory synapses.. J Neurosci.

[78] Rusakov DA, Kullmann DM (1998). Extrasynaptic glutamate diffusion in the hippocampus: ultrastructural constraints, uptake, and receptor activation.. J Neurosci.

[79] Angulo MC, Kozlov AS, Charpak S, Audinat E (2004). Glutamate released from glial cells synchronizes neuronal activity in the hippocampus.. J Neurosci.

[80] Fellin T, Pascual O, Gobbo S, Pozzan T, Haydon PG (2004). Neuronal synchrony mediated by astrocytic glutamate through activation of extrasynaptic NMDA receptors.. Neuron.

[81] Sasaki T, Kuga T, Namiki S, Matsuki N, Ikegaya Y (2011). Locally synchronized astrocytes.. Cerebral Cortex.

[82] Tian GF, Azmi H, Takahiro T, Xu Q, Peng W (2005). An astrocytic basis of epilepsy.. Nature Med.

[83] Oliet SHR, Piet R, Poulain DA (2001). Control of glutamate clearance and synaptic efficacy by glial coverage of neurons.. Science.

[84] Bezzi P, Volterra A (2001). A neuron-glia signalling network in the active brain.. Curr Opinion Neurobiol.

[85] Aguado F, Espinosa-Parrilla JF, Carmona MA, Soriano E (2002). Neuronal activity regulates correlated network properties of spontaneous calcium transients in astrocytes *in situ*.. J Neurosci.

[86] Halassa MM, Fellin T, Takano H, Dong JH, Haydon PG (2007). Synaptic islands defined by the territory of a single astrocyte.. J Neurosci.

[87] Dan Y, Poo MM (2004). Spike timing-dependent plasticity of neural circuits.. Neuron.

[88] Henneberger C, Papouin T, Oliet SHR, Rusakov DA (2010). Long-term potentiation depends on release of D-serine from astrocytes.. Nature.

[89] Santello M, Volterra A (2010). Astrocytes as aide-mémoires.. Nature.

[90] Abbott LF, Nelson SB (2000). Synaptic plasticity: taming the beast.. Nature.

[91] Li Y, Rinzel J (1994). Equations for InsP_3_ receptor-mediated [Ca^2+^]*_i_* oscillations derived from a detailed kinetic model: A Hodgkin-Huxley like formalism.. J Theor Biol.

[92] Diamond JS, Jahr CE (2000). Synaptically released glutamate does not overwhelm transporters on hippocampal astrocytes during high-frequency stimulation.. J Neurophysiol.

[93] Cochilla AJ, Alford S (1998). Metabotropic glutamate receptor-mediated control of neurotransmitter release.. Neuron.

[94] Gereau RW, Conn J (1995). Multiple presynaptic metabotropic glutamate receptors modulate excitatory and inhibitory synaptic transmission in hippocampal area CA1.. J Neurosci.

[95] Baskys A, Malenka RC (1991). Agonists at metabotropic glutamate receptors presynaptically inhibit EPSCs in neonatal rat hippocampus.. J Physiol.

