# Supplementary figures and images for: A Tale of Two Stories: Astrocyte Regulation of Synaptic Depression and Facilitation
Source: PLoS Comput Biol. 2011 Dec 1;7(12):e1002293. doi: 10.1371/journal.pcbi.1002293 (PMC3228793; doi:10.1371/journal.pcbi.1002293)

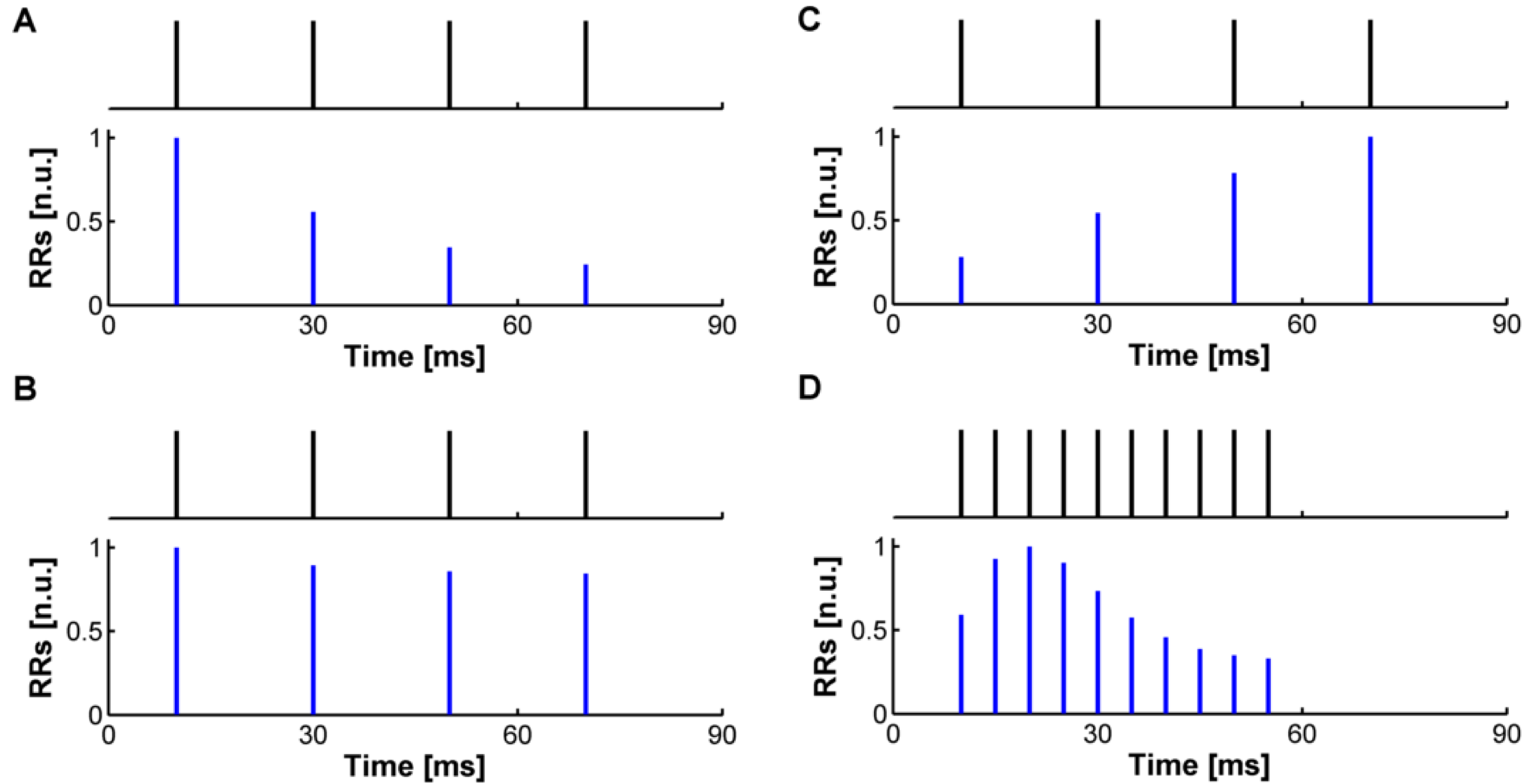

Supplement: Figure S1 — Conditions for short-term depression and facilitation in the TM model. Short-term plasticity in the TM model is brought forth by inherent synaptic parameters such as Ωd, Ωf and U 0, and the frequency of incoming spikes. (A,B) Depressing synapses are generally characterized by Ωf>Ωd. In these latter, input spikes at f in>Ωd (A) mark the onset of short-term depression (STD) due to fast depletion of the pool of releasable resources. (B) Alternatively, STD can also be observed in high-fidelity synapses, namely synapses characterized by high values of U 0. (C,D) Facilitating synapses instead are characterized by Ωf<Ωd and low release probability. In these latter (C), incoming spikes at Ωf<f in<Ωd (or f in>Ωd, Ωf) build up presynaptic residual Ca2+ levels, increasing the synaptic release, thus evidencing facilitation. (D) However, the progressive increase of release probability due to facilitation leads to concomitant growing depletion of the releasable pool and STD eventually takes over facilitation. Legend: input presynaptic spikes are in black, released resources (RRs, blue) are normalized with respect to their maximum (A,B: RR max = 0.5; C: RR max = 0.18; D: RR max = 0.2). Parameters: (A) Ωd = 2 s−1, Ωf = 1000 s s−1, U 0 * = 0.5, f in = 50 Hz; (B) Ωd = 20 s−1, Ωf = 1000 s−1, U 0 = 0.5, f in = 50 Hz; (C) Ωd = 100 s−1, Ωf = 1.25 s−1, U 0 = 0.05, f in = 50 Hz; (D) Ωd = 10 s−1, Ωf = 1.25 s−1, U 0 = 0.1, f in = 200 Hz. (TIF) [file pcbi.1002293.s001.tif]

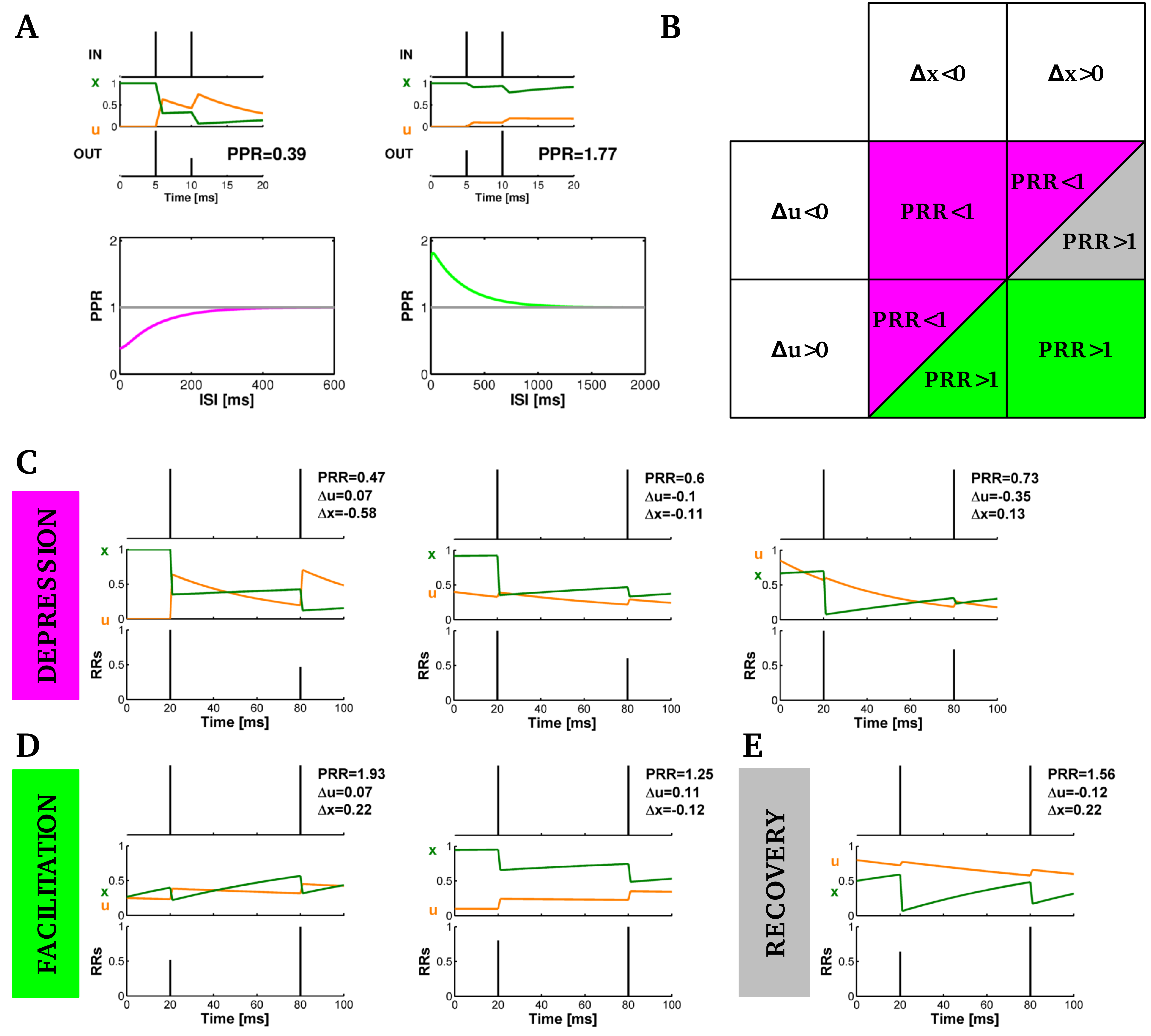

Supplement: Figure S2 — Paired-pulse plasticity. (A, top) In a typical paired-pulse stimulus protocol, a pair of spikes with controlled interspike interval is delivered to the synapse and synaptic response to the second spike (RR 2) is compared to synaptic response to the first spike (RR 1) by means of paired-pulse ratio, defined as . (A, left) Values of PPR less than 1 mark paired-pulse depression (PPD) as in such conditions RR 21, then RR 2>RR 1 and paired-pulse facilitation (PPF) is observed. The farther the PPR from unity, the stronger the PPD (or PPF). (A, bottom) The value of PPR critically depends on the interspike interval (ISI) of spike pairs and approaches zero for very long ISIs reflecting the fact that short-term synaptic plasticity is a transient phenomena. (B) For a generic input spike trains, the PPR between consecutive spikes in a pair is not sufficient to distinguish between PPD and PPF. Depending on the spike timing and on the past synaptic activity in fact, PPR>1 could also result from sufficient reintegration of the pool of releasable resources (Δx>0), despite a decrease of residual Ca2+ between the two spikes in a pair (i.e. Δu<0). This situation corresponds to a different form of synaptic plasticity dubbed as “recovery from depression” [13]. (C–E) Examples of different short-term plasticity mechanisms listed in the Table (A) displayed by the TM model. Parameters: (A, left) Ωd = 10 s−1, Ωf = 100 s−1, U 0 = 0.7, RR max = 0.7; (A, right) Ωd = 100 s−1, Ωf = 33 s−1, U 0 = 0.05; (C, left) Ωd = 2 s−1, Ωf = 20 s−1, U 0 = 0.65; (C, middle) Ωd = 3.33 s−1, Ωf = 10 s−1, U 0 = 0.1; (C, right) Ωd = 4 s−1, Ωf = 20 s−1, U 0 = 0.1; (D, left) Ωd = 10 s−1, Ωf = 3.33 s−1, U 0 = 0.2; (C, right) Ωd = 5 s−1, Ωf = 1 s−1, U 0 = 0.16; (E) Ωd = 10 s−1, Ωf = 5 s−1, U 0 = 0.2. (TIF) [file pcbi.1002293.s002.tif]

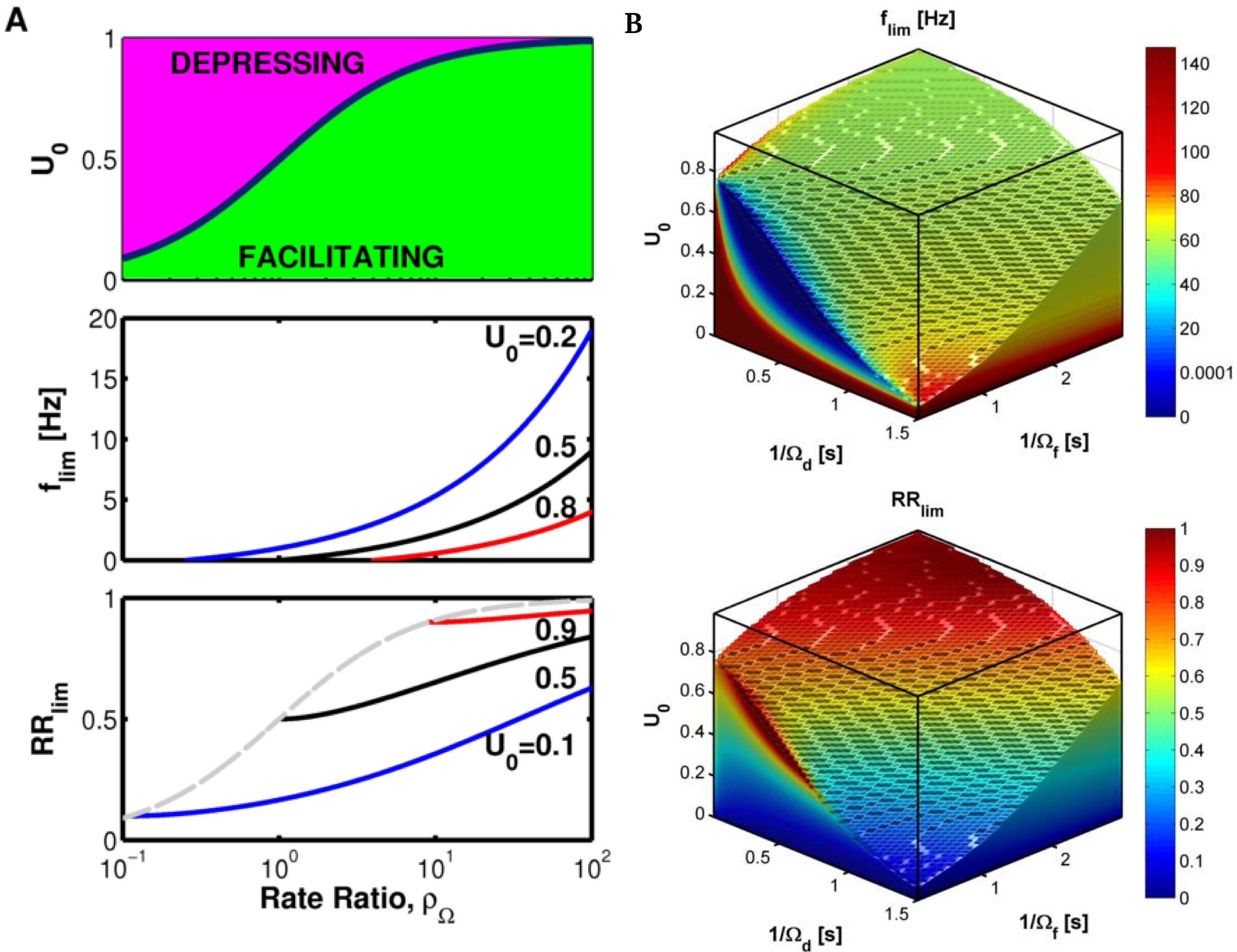

Supplement: Figure S3 — The switching threshold in the TM model. (A, top) Mapping of depressing (red) and facilitating (green) synapses in the parameter plane U 0 vs. ρ Ω = Ωd/Ωf. The two types of synapses are separated by the switching threshold (black line) given by (equation S39). (A, middle) The limiting frequency f lim of a facilitating synapse coincides with the peak frequency of maximal steady-state release of neurotransmitter is maximal (see also Figure 2D). For fixed facilitation rates (i.e. Ωf = const), such limiting frequency increases with ρ Ω, namely with faster rates (Ωd) of reintegration of synaptic resources. In such conditions in fact the larger Ωd, the higher the rate of input spikes before the onset of depression. For the same reason, higher f lim are also found in correspondence of lower values of synaptic basal release probability U 0 at given ρ Ω. (A, bottom) The peak of released resources at the limiting frequency (equation S41) instead increases with U 0 to the detriment of its range of variation (recall in fact, that 0<RR lim<1). (B, top) Facilitation regions in the parameter space and mapping therein of f lim (equation S40) and (B, bottom) RR lim (equation S41), show strong nonlinear dependence of both quantities on synaptic parameters. (TIF) [file pcbi.1002293.s003.tif]

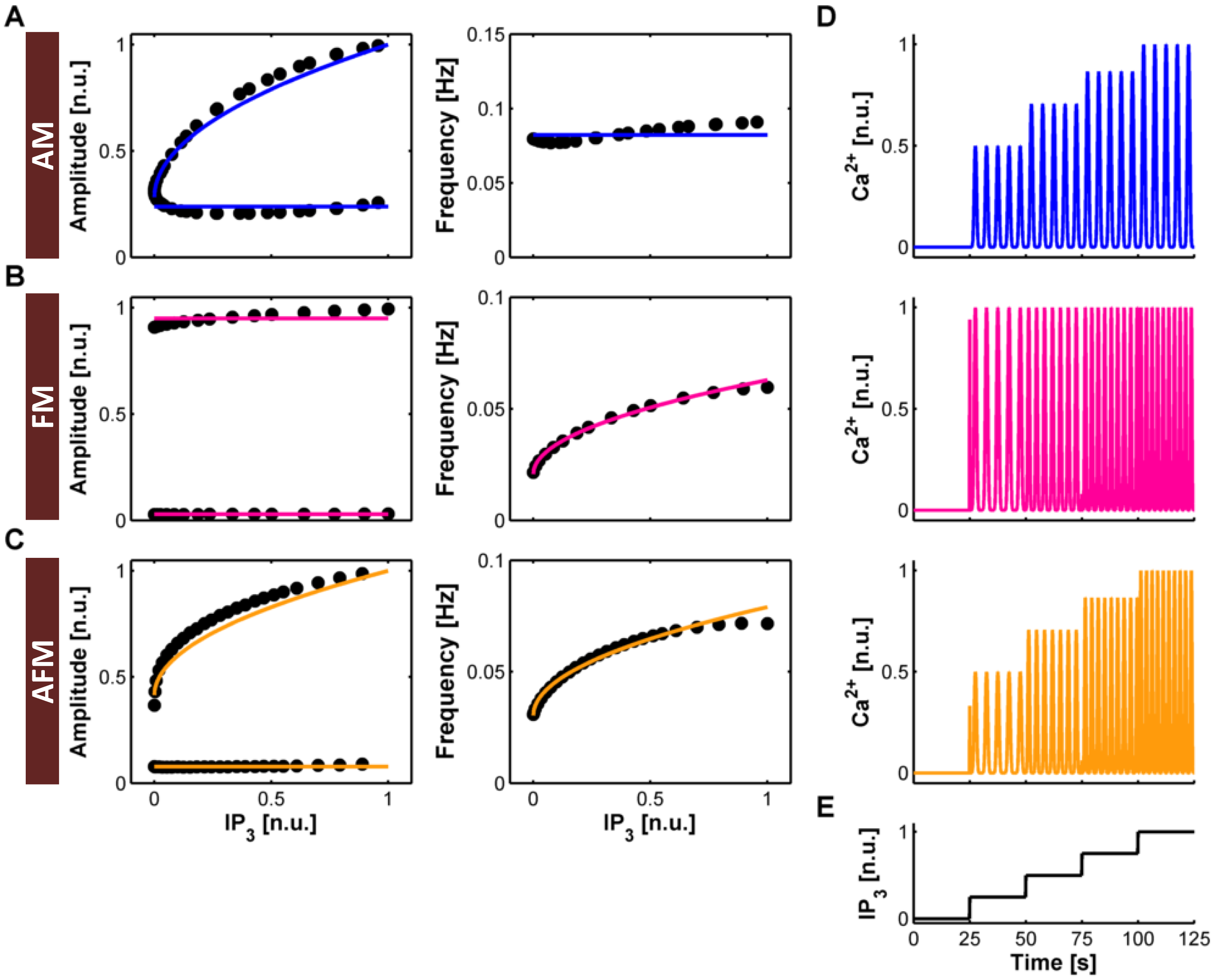

Supplement: Figure S4 — Astrocyte calcium dynamics. (A–C) Superposition of stereotypical functions (solid line) on numerically-solved (black circles) amplitude and frequency of (A) AM-encoding, (B) FM-encoding and (C) AFM-encoding Ca2+ oscillations as obtained from the Li-Rinzel model of Ca2+ dynamics [76], [91] (see Text S1, Section I.2). (D) Corresponding Ca2+ oscillations pertaining to these three modes for the case of an IP3 stimulus as in (E). Data in (A–C, left and middle) are from [76]. For convenience, only persistent oscillations are considered. The oscillatory range is rescaled between 0 and 1 and amplitude of oscillations is normalized with respect to the maximal Ca2+ concentration. Data were fitted by equations (S4, S5, S6) with assuming I b = 0. (A) C 0 = 0.239, m 0 = 0.256, k = 0.750; (B) C 0 = 0.029, C max = 0.939, m 0 = 0.210, k = 0.470, f C = 0.1 Hz; (C) C 0 = 0.079, m 0,AM = 0.449, k AM = 0.611, m 0,FM = 0.310, k FM = 0.480, f C = 0.1 Hz. (D) C 0 = 0, m 0,AM = 0, m 0,FM = 0 Hz, k AM = 1, k FM = 1, f C = 0.1 Hz, I b = 0. (TIF) [file pcbi.1002293.s004.tif]

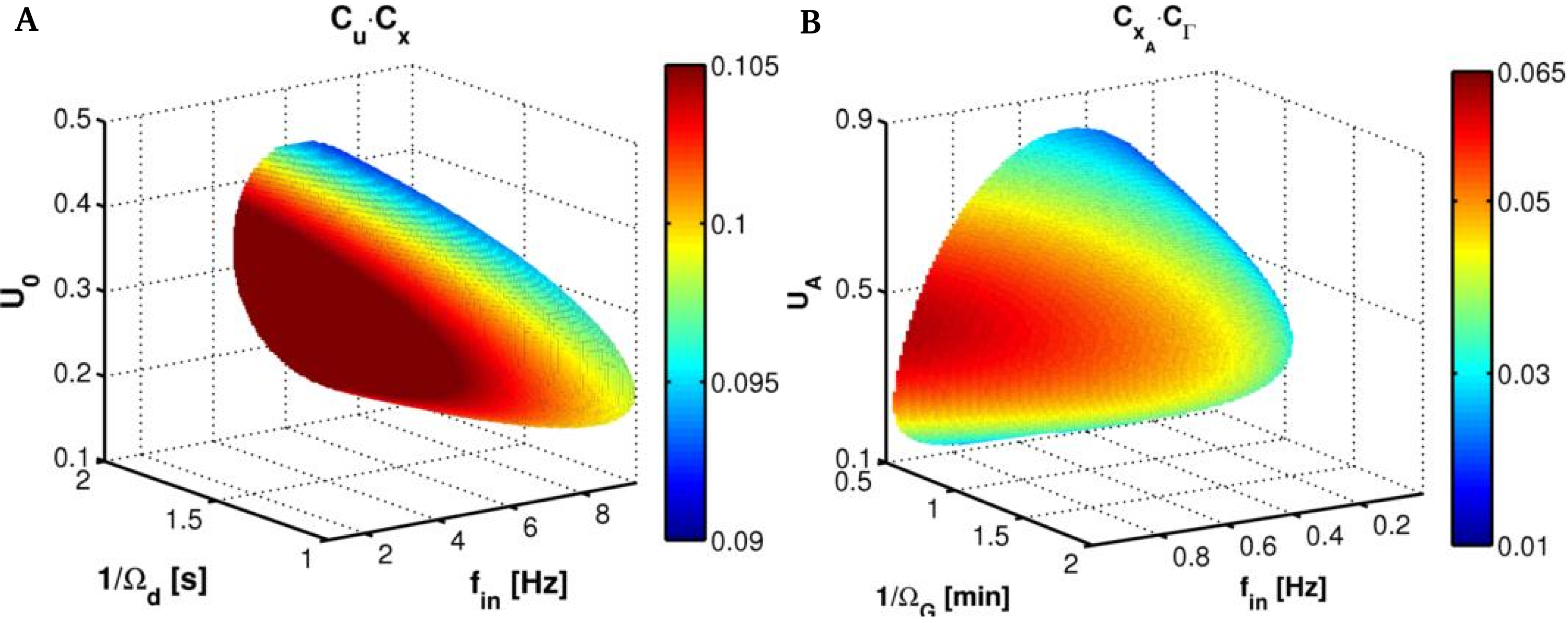

Supplement: Figure S7 — Range of validity of the mean-field description. (A) Product of coefficients of variations for the two synaptic variables x and u as a function of frequency, allows to estimate the region of validity of the mean-field description (equations S31–S32). In particular, in the domain of the parameter space considered in this study, the error made by averaging exceeds 10% only for a narrow region of such space confined between 4<f in<6 Hz. (B) Analogous considerations hold for averaging of equations (S7, S18, S19). Mapping of the product of coefficients of variations of x A and Γ shows that in this case, the error is less than than 7% in the whole parameter space. Parameters: (A) Ωf = 2.5 s−1; (B) O G = 1.5 µMs−1. Other parameters as in Table S1. (TIF) [file pcbi.1002293.s007.tif]

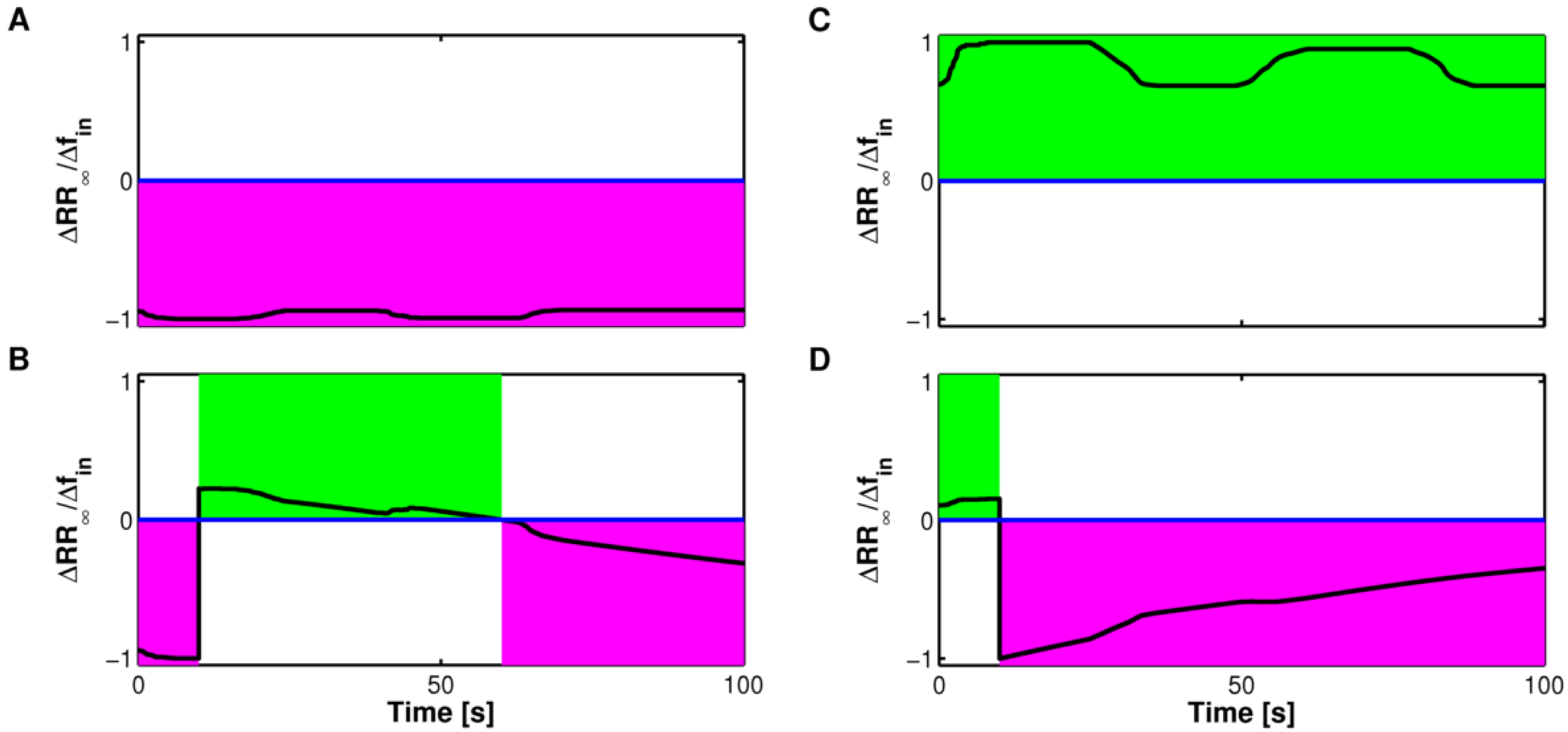

Supplement: Figure S9 — Slope analysis. Estimation of the trial-averaged slope of the synaptic frequency response curve for any value in time of the input frequency f in (that is the derivative of (equation 3) with respect to f in) allows characterization of any transitions of synaptic plasticity. The method is alternative to that outlined in Figure 7, and relies on the observation that in our model of synaptic plasticity, short-term facilitation is likely to occur whenever for given input rates, otherwise short-term depression is predominant (see also Text S1, Section II.1). Letters correspond to those in Figure 7, and refer to results of slope analysis for the corresponding cases therein, that is: (A) depressing synapse without and (B) with release-decreasing astrocyte, and (C) facilitating synapse without and (D) with release-increasing astrocyte. Green-shaded areas denote predominant PPF, magenta-shaded areas stand for predominant PPD. Slope values are normalized by their maximum absolute value. Parameters are as in Table S1. (TIF) [file pcbi.1002293.s009.tif]

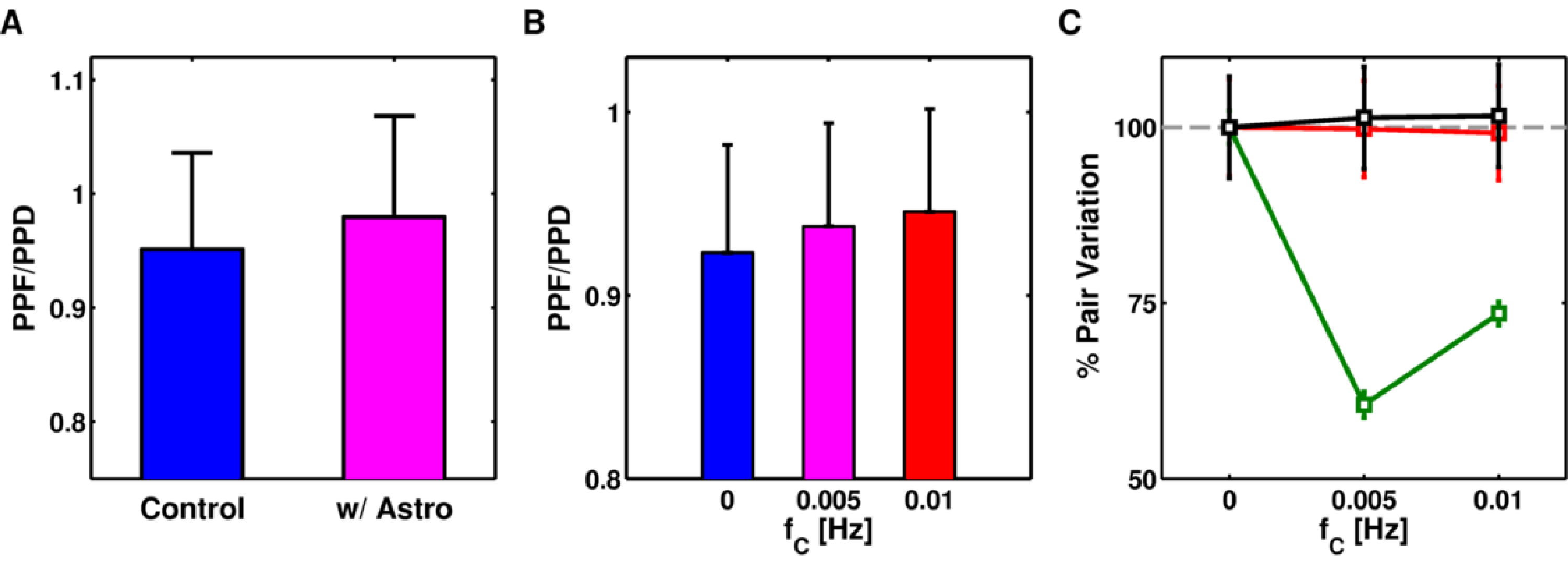

Supplement: Figure S10 — Release-decreasing astrocyte on a facilitating synapse. (A) Analysis of paired-pulse plasticity in presence of a single glutamate exocytotic event from the astrocyte (same conditions of Figure 5A) shows an increase of the number of facilitated spike pairs (green bar) with respect to “Control” simulations (i.e. without astrocyte) (blue bar) (bar+error bar: mean+standard deviation). (B) Moreover, the larger the frequency of glutamate release from the astrocyte, the stronger the effect. (C) Detailed analysis of the different forms of short-term plasticity ongoing within spike pairs – PPF (dark green), PPD (red) and “recovery from depression” (black) – reveals that the increase of the ratio PPF/PPD detected in (A–B) is mainly imputable to an increase of PPF accompanied by a reduction of recovery from depression. These results confirm the general notion discussed in the text that the effect of a release-decreasing astrocyte coincides with an increase of paired-pulse facilitation (PPF) (see also Figure 7D). Nonetheless, we note that this effect is less pronounced than in a depressing synapse (compare Figures 5A with S9A and Figure 9A with S9B). Data based on n = 100 Poisson input spike trains with average rate as in Figure 7C. Data in (C) are normalized with respect to their “Control” value: PPF = 197, PPD = 205, recovery = 27. Parameters as in Table S1 with α = 0. (TIF) [file pcbi.1002293.s010.tif]

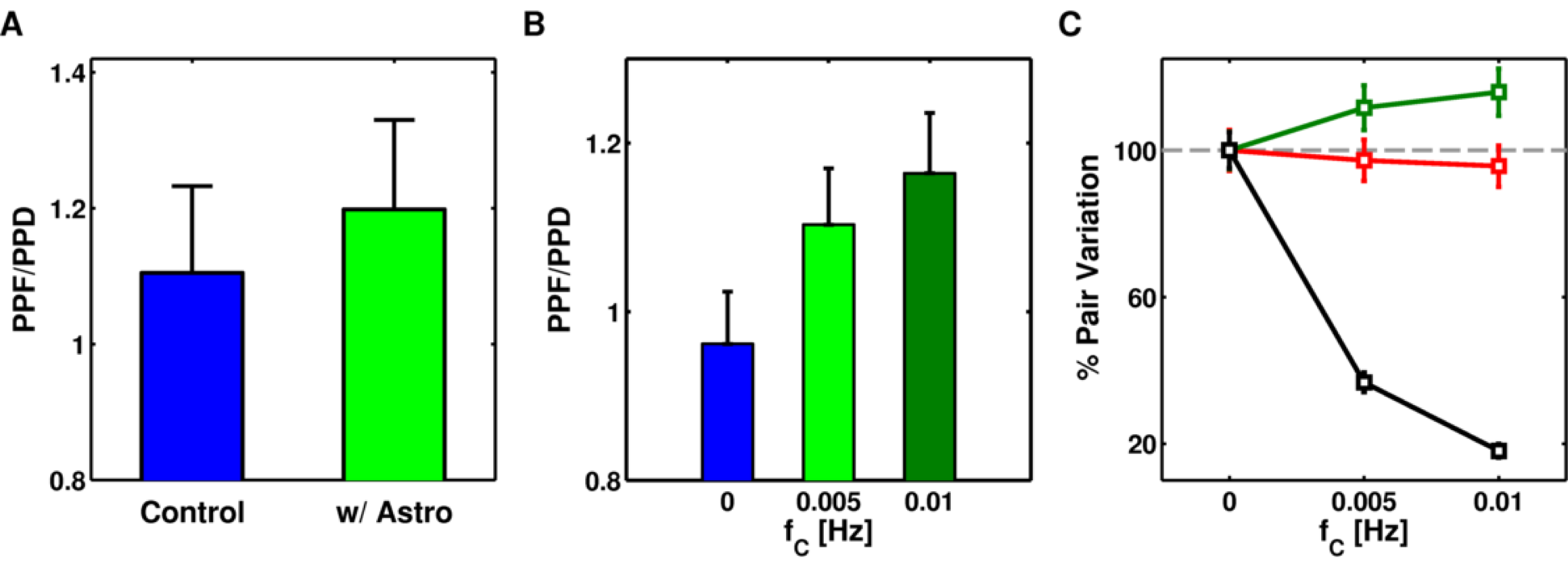

Supplement: Figure S11 — Release-increasing astrocyte on a depressing synapse. (A) Analysis of paired-pulse plasticity either for a single (same conditions of Figure 5B) and (B) for persistent glutamate exocytosis from the astrocyte, shows an increase of facilitated spike pairs (magenta/red bars) with respect to the “Control” simulations (i.e. in absence of the astrocyte) (blue bars). (C) A closer inspection on the nature of ongoing paired-pulse plasticity (PPF: green, PPD: red and “recovery from depression”: black) reveals that such increase is actually caused by an increase of recovery from depression (Control: PPF = 4, PPD = 146, recovery = 135). Bar+Error bar: Mean+Standard deviation. Data based on n = 100 Poisson input spike trains with average rate as in Figure 7A. Parameters as in Table S1 with α = 1. (TIF) [file pcbi.1002293.s011.tif]
