# Supplementary material for: A Tale of Two Stories: Astrocyte Regulation of Synaptic Depression and Facilitation
Source: PLoS Comput Biol. 2011 Dec 1;7(12):e1002293. doi: 10.1371/journal.pcbi.1002293 (PMC3228793; doi:10.1371/journal.pcbi.1002293)
Supplement: Table S1 — Table of parameters of the model of astrocyte-synapse interactions, and corresponding values used in the simulations. (DOC) [file pcbi.1002293.s012.doc]

**Table S1**.

Model parameters and respective values used in simulations.

| **Parameter** | **Description** | **Value** | **Units** |
| --- | --- | --- | --- |
| ***U*_0_*** | Basal probability of synaptic glutamate release | <0.05 – 0.9^a^ | – |
| **Ω_d_** | Rate of recovery of released synaptic vesicles | 0.5 – 100^a^ | s^-1^ |
| **Ω_f_** | Rate of synaptic facilitation | 0.5 – 2^a^ | s^-1^ |
| **α** | Effect parameter of astrocyte regulation of synaptic release | 0 – 1 | – |
| ***U*_A_** | Basal release probability of astrocytic glutamate vesicles | <0.8 (0.6) | – |
| **Ω_A_** | Rate of recovery of released astrocytic vesicles | 0.01 – 450 (0.6) | s^-1^ |
| ***n*_v_** | Number of readily releasable astrocytic vesicles | 1 – 6 (4) |  |
| ***G*_v_** | Glutamate content of astrocytic vesicles | 20 – 150 (50) | mM |
| ***V_v_*** | Volume of astrocytic vesicles | 2 – 700 ·10^-21^ | dm^3^ |
| ***V*_e_** | Mixing volume of released astrocytic glutamate | ~10^-16^ | dm^3^ |
| ***ρ*_A_** | Volume ratio *V*_v_/*V*_e_ | 6.5·10^-4^ | – |
| **Ω_c_** | Glutamate clearance rate | >50 – 150 (60) | s^-1^ |
| ***O*_G_** | Onset rate of astrocyte modulation | 0.2 – 2 (1.5) | μM^-1^ s^-1^ |
| **Ω_G_** | Recovery rate of astrocyte modulation | <0.5 – 1.2 (0.5) | min^-1^ |
| ***f*_C_** | Frequency of Ca^2+^ oscillations in the astrocyte | 0.01 – 1 (0.1) | Hz |
| ***C*_0_** | Basal Ca^2+^ concentration | 0 | – |
| ***I*_b_** | IP_3_ threshold concentration for astrocyte Ca^2+^ dynamics | 0 | – |
| ***k*** | Scaling factor for the IP_3_ signal | 1 | – |
| ***w*** | Shape factor | 20^b^ | – |
| ***C*_thr_** | Ca^2+^ threshold for astrocyte exocytosis of glutamate | 0.13 – 0.8 (0.4) | – |
| ***φ*_C_** | Phase of Ca^2+^ oscillations | 0 | rad |

^a^ In the simulations, a *depressing* synapse was characterized by:  Ω_d_ = 2 s^-1^, Ω_f_ = 3.33 s^-1^, *U*_0_* = 0.5; whereas a *facilitating* synapse was given by:  Ω_d_ = 2 s^-1^, Ω_f_ = 2 s^-1^, *U*_0_* = 0.15.

^b^ ***w*** must be a positive even integer.
