# Supplementary material for: A Tale of Two Stories: Astrocyte Regulation of Synaptic Depression and Facilitation
Source: PLoS Comput Biol. 2011 Dec 1;7(12):e1002293. doi: 10.1371/journal.pcbi.1002293 (PMC3228793; doi:10.1371/journal.pcbi.1002293)
Supplement: Text S1 — Detailed description and derivation of the model of astrocyte-synapse interaction and analytical methods. (PDF) [file pcbi.1002293.s013.pdf]

A tale of two stories: astrocyte regulation of synaptic depression and facilitation

Maurizio De Pittà, Vladislav Volman, Hugues Berry, and Eshel Ben-Jacob

SUPPLEMENTARY TEXT S1

The organization of the present Supporting Text is as following.

I. **Model description**

1. The Tsodyks-Markram model of synaptic release
2. Astrocytic calcium dynamics
3. Glutamate exocytosis from the astrocyte
4. Glutamate time course in the extrasynaptic space
5. Astrocyte modulation of synaptic release

II. **Model analysis**

1. Mechanisms of short-term depression and facilitation in the TM model of synaptic release
2. Characterization of paired-pulse depression and facilitation
3. Mean-field description of synaptic release
4. Frequency response and limiting frequency of a synapse
5. Mean-field description of astrocyte-to-synapse interaction

III. **Appendix A:** Model equations

IV. **Appendix B:** Estimation of the coefficient of variation of Γ

V. **Appendix C:** Parameter estimation

VI. **References**

I. Model description

1. The Tsodyks-Markram model of synaptic release

Mechanisms of short-term synaptic depression and facilitation at excitatory hippocampal synapses can be realistically mimicked by the Tsodyks-Markram (TM) model of activity-dependent synapse [1, 2]. The model considers two variables *u* and *x*, which respectively correlate with the state of occupancy of the calcium (Ca^{2+}) sensor of synaptic glutamate exocytosis and the fraction of glutamate available for release at any time [3].

At resting (basal) conditions, the occupancy of the sensor is minimal so that $u = 0$. Each presynaptic spike occurring at time t_i (and modeled by a Dirac delta) triggers Ca^{2+} influx into the presynaptic terminal thus increasing *u*. In particular, the model assumes that a fraction U_0 of 1-*u* vacant states of the sensor is first occupied by incoming Ca^{2+} ions [4, 5], and is following recovered at rate Ω_f. Hence, $u(t)$ evolves according to the equation

$$\dot{u} = -\Omega_f u + U_0 \sum_i (1-u)\delta(t-t_i) \tag{1}$$

Following the increase of *u* upon action potential arrival, an amount *ux* of presynaptic glutamate is released into the cleft while the pool of synaptic glutamate (assumed to be constant in size) is replenished at rate Ω_d. The equation for $x(t)$ then reads

$$\dot{x} = \Omega_d (1-x) - \sum_i ux\delta(t-t_i) \tag{2}$$

The fraction of released glutamate resources *RR*, upon arrival of a presynaptic spike at $t = t_i$ is given by

$$RR(t_i) = u(t_i^+)\cdot x(t_i^-) \tag{3}$$

where $u(t_i^+)$ and $x(t_i^-)$ denote the values of *u* and *x* immediately respectively *after* and *before* the spike at $t = t_i$.

On a par with the classical quantal model of synaptic transmission [6], $x(t)$ is analogous to the probability of a glutamate-containing vesicle to be available for release at any time *t*; $u(t)$ corresponds instead to the probability of release of a docked vesicle; and finally $RR(t_i)$ represents the probability of release (for every release site) at the time t_i of the spike [7]. Then the parameter U_0 in the above equation (1), coincides with the value reached by *u* immediately after the first spike of a train, starting from resting conditions (i.e. $u(0) = 0$, $x(0) = 1$). Since this situation also corresponds to the case of basal stimulation, that is of a stimulus at very low frequency, U_0 can be regarded as the basal value of synaptic release

probability too [8]. The TM formulation ignores the stochastic nature of synaptic release and reproduces the average synaptic release event generated by any presynaptic spike train [7].

2. Astrocytic calcium dynamics

Intracellular Ca^{2+} concentration in astrocytes can be modulated by several mechanisms [9]. These include Ca^{2+} influx from the extracellular space or controlled release from intracellular Ca^{2+} stores such as the endoplasmic reticulum (ER) and mitochondria [10]. Inositol trisphosphate- (IP_3-) dependent Ca^{2+}-induced Ca^{2+} release (CICR) from the ER is considered though the primary mechanism responsible of intracellular Ca^{2+} dynamics in astrocytes [11]. In this latter, IP_3 second messenger binds to receptors localized on the cytoplasmic side of the ER which open releasing Ca^{2+} from the ER in an autocatalytic fashion [10]. Due to the nonlinear properties of such receptor/channels, CICR is essentially oscillatory [12]. The pattern of Ca^{2+} oscillations depends on the intracellular IP_3 concentration, hence one can think of the Ca^{2+} signal as being an encoding of information on this latter [13]. Notably, this information encoding could use amplitude modulations (AM), frequency modulations (FM) or both modulations (AFM) of Ca^{2+} oscillations [13-17]. Accordingly, we consider stereotypical functions that reproduce all these possible encoding modes (Figure S4). Let $C(t)$ denote the Ca^{2+} signal and $m_i(t)$ (with i = AM, FM) the modulating signal related to the IP_3 concentration. Then, AM-encoding Ca^{2+} dynamics can be modeled by:

$$C(t) = C_0 + m_{\mathrm{AM}}(t)\sin^w\left(2\pi f_{\mathrm{C}} t + \varphi_{\mathrm{C}}\right) \tag{4}$$

FM-encoding Ca^{2+} dynamics instead can be mimicked by the equation:

$$C(t) = C_0 + \sin^w\left(2\pi \cdot m_{\mathrm{FM}}(t) \cdot f_{\mathrm{C}} t + \varphi_{\mathrm{C}}\right) \tag{5}$$

Eventually, AFM-encoding comprises both the above in the single generic equation:

$$C(t) = C_0 + m_{\mathrm{AM}}(t)\sin^w\left(2\pi \cdot m_{\mathrm{FM}}(t) \cdot f_{\mathrm{C}} t + \varphi_{\mathrm{C}}\right) \tag{6}$$

In the above equations, f_C and φ_C denote the frequency and the phase of Ca^{2+} oscillations respectively. Moreover, the exponent w is taken as positive even integer to adjust the shape of Ca^{2+} oscillations, i.e. from sinusoidal to more pulse-like oscillations (namely pulses of width much smaller than their wavelength) [14, 18].

The exact functional form of $m_i(t)$ depends on inherent cellular properties of the astrocyte [13, 15, 19]. Notwithstanding, several theoretical studies showed that for increasing IP_3 concentrations, Ca^{2+} oscillations are born via some characteristic bifurcation pathways [20]. In particular,

while AM Ca^{2+} dynamics could be explained by a supercritical Hopf bifurcation, FM features are born via saddle-node on homoclinic bifurcation [13-15]. Notably, both these bifurcations are characterized by similar functional dependence on the bifurcation parameter, i.e. the IP_3 concentration in our case, respectively for amplitude and period of oscillations at their onset [21, 22]. This scenario thus allows considering analogous $m_i(t)$ in equations (4-6) of the form $m_i(t) = k_i\sqrt{(IP_3(t) - I_b)}$, where I_b is the threshold IP_3 concentration that triggers CICR and k_i is a scaling factor [21]. We assume that $IP_3(t)$ is externally driven either by gap junction-mediated intercellular diffusion from neighboring astrocytes [23-25], or by external stimulation of the cell [18, 26-28]. In this fashion we can control the pattern of Ca^{2+} oscillations yet preserving the essence of the complex network of chemical reactions underlying IP_3/Ca^{2+}-coupled signals [14, 24].

3. Glutamate exocytosis from the astrocyte

Calcium induced Ca^{2+} release triggered by IP_3 is observed to induce glutamate exocytosis from astrocytes [29, 30]. Additional data also suggest an involvement of ryanodine/caffeine-sensitive internal Ca^{2+} stores [31], notwithstanding evidence for the existence of RyR-mediated Ca^{2+} signaling in astrocytes are contradictory [32] and this possibility is not considered in this study.

A large amount of evidence suggests that glutamate exocytosis from astrocytes resembles its synaptic homologous [32, 33]. Astrocytes indeed possess a vesicular compartment that is competent for regulated exocytosis [34]. Glutamate-filled vesicles in astrocytic processes in rodents' dentate gyrus closely resemble synaptic vesicles in excitatory nerve terminals [30, 35]. Similarly to synapses, astrocytes also express SNARE proteins necessary for exocytosis [36] as well as the proteins responsible for sequestering glutamate into vesicles [37]. Indeed synaptic-like plasma-membrane fusion, trafficking and recycling of astrocytic glutamate vesicles were observed [38-40] and quantal glutamate release hallmarking vesicle exocytosis [6] was measured accordingly [28, 40, 41], [42]]. Moreover, experiments suggest that release of glutamate is likely much faster than its reintegration [38, 40] in a fashion akin to that of synaptic exocytosis [43].

Based on these arguments, we model astrocytic glutamate exocytosis similarly to synaptic release. Thus, we postulate the existence of an astrocytic pool x_A of releasable glutamate resources that is limited and constant in size. Then, upon any "proper" intracellular Ca^{2+} increase at time τ_i, an amount $U_A x_A$ of such

resources is released into the extrasynaptic space and is later reintegrated into the pool at rate Ω_A. Hence, the equation for x_A reads

$$\dot{x}_A = \Omega_A (1 - x_A) - U_A \sum_i x_A \, \delta(t - \tau_i) \tag{7}$$

where the parameter U_A is the astrocytic analogous of synaptic basal release probability U_0 (equation 1). The instants τ_i at which glutamate is released from the astrocyte are dictated by the Ca^{2+} dynamics therein. While Ca^{2+} oscillations trigger synchronous release, sustained nonoscillatory Ca^{2+} increases were observed to induce glutamate exocytosis only during their initial rising phase [28, 44]. Furthermore, glutamate release occurs only if intracellular Ca^{2+} concentration exceeds a threshold value [27, 28]. Therefore, in agreement with these experimental observations, we assume that astrocytic glutamate release occurs at any time $t = \tau_i$ such that $C(\tau_i) = C_{\text{thr}}$ and $dC/dt|_{\tau_i} > 0$, where $C(t)$ is described by equations (4-6) and C_{thr} stands for the Ca^{2+} threshold of glutamate exocytosis.

This description lumps into a single release event the *overall* amount of glutamate released by a Ca^{2+} increases beyond the threshold, independently of the underlying mechanism of exocytosis, which could involve either a single or multiple vesicles [30, 39, 45]. In this latter scenario, the error introduced by our description might be conspicuous if asynchronous release occurs in presence of fast clearance of glutamate in the extrasynaptic space. Nonetheless, further experiments are needed to support this possibility [29].

4. Glutamate time course in the extrasynaptic space

A detailed modeling of glutamate time course in the extrasynaptic space (ESS) is beyond the scope of this study. Accordingly, we simply assume that the ESS concentration of glutamate G_A is mainly dictated by: (1) the frequency and the amount of its release from the astrocyte; and (2) its clearance rate due to astrocytic glutamate transporters along with diffusion away from the release site [46-48]. In other words, G_A evolves according to the generic equation

$$\dot{G}_A = v_{\text{release}} - v_{\text{diffusion}} - v_{\text{uptake}} \tag{8}$$

where v_{release}, $v_{\text{diffusion}}$ and v_{uptake} respectively denote the rates of glutamate release, diffusion and uptake and are following detailed. Binding of astrocyte-released glutamate by astrocytic glutamate receptors [9, 30] is also not included in equation (8) for simplicity. Activation of such receptors by excess glutamate in the ESS is in fact strongly limited by fast glutamate buffering by astrocytic transporters [49] and is

further supported by the experimental evidence that autocrine receptor activation does not essentially affect intracellular Ca^{2+} dynamics in the astrocyte [50, 51].

Glutamate release in our description occurs instantaneously by exocytosis from the astrocyte. In particular, the fraction RR_A of astrocytic glutamate resources released at $t = \tau_i$ by the i-th Ca^{2+} increase beyond the threshold C_{thr} (Section I.3), is given by (equation 7):

$$RR_\mathrm{A}(\tau_i) = U_\mathrm{A} x_\mathrm{A}(\tau_i^-) \quad (9)$$

If we suppose that the total number of releasable glutamate molecules in the astrocyte is M then, the maximal contribution to glutamate concentration in the ESS, that is for $RR_\mathrm{A}(\tau_i) = 1$, equals to

$$G_\mathrm{max} = M / N_\mathrm{A} V_\mathrm{e} \quad (10)$$

where N_A is the Avogadro constant and V_e is the volume of the ESS "of interest", namely the ESS comprised between the astrocytic process where release occurs, and the presynaptic receptors targeted by astrocytic glutamate. In general though, the contribution G_{rel} to glutamate in the ESS is only a fraction $RR_A(\tau_i)$ of the maximal contribution, that is

$$G_\mathrm{rel}(\tau_i) = G_\mathrm{max} \cdot RR_A(\tau_i) \quad (11)$$

We can further express then the number M of glutamate molecules in the astrocyte in terms of parameters that can be experimentally estimated noting that, M equals the number of molecules per vesicle M_v, times the number of vesicles available for release n_v. In turn, the number of molecules per synaptic vesicle can be estimated to be proportional to the product of the vesicular glutamate concentration G_v, times the vesicular volume V_v, being [52]

$$M_\mathrm{v} = N_\mathrm{A} G_\mathrm{v} V_\mathrm{v} \quad (12)$$

Under the hypothesis that all glutamate-containing vesicles in the astrocyte are identical both in size and content, the overall number of glutamate molecules in the astrocyte can be estimated as

$$M = n_\mathrm{v} M_\mathrm{v} = n_\mathrm{v} N_\mathrm{A} G_\mathrm{v} V_\mathrm{v} \quad (13)$$

Accordingly, replacing equations (10) and (13) in (11) provides the generic exocytosis contribution to glutamate concentration in the ESS, which can be written as

$$G_\mathrm{rel}(\tau_i) = \rho_\mathrm{A} n_\mathrm{v} G_\mathrm{v} \cdot RR_\mathrm{A}(\tau_i) \quad (14)$$

where $\rho_\mathrm{A} = V_\mathrm{v} / V_\mathrm{e}$ is the ratio between vesicular volume and the volume of the ESS of interest. The total contribution of astrocytic glutamate exocytosis to the time course of glutamate in the ESS, is

therefore the sum of all contributions by each single release event (given by equation 14). Hence, the rate of glutamate release in equation (8) can be written as

$$v_{\text{release}} = \sum_i G_{\text{rel}}(\tau_i) = \rho_{\text{A}} n_{\text{v}} G_{\text{v}} \sum_i RR_{\text{A}}(\tau_i) \tag{15}$$

Let us consider now the glutamate degradation terms in equation (8). Glutamate clearance due to lateral diffusion out of the ESS volume of interest follows Fick's first law of diffusion [53]. Then, assuming ESS isotropy, the rate of decrease of ESS glutamate concentration can be taken proportional to the concentration of astrocyte-released glutamate (G_{A}) by a factor r_{d} which stands for the total rate of glutamate diffusion, that is

$$v_{\text{diffusion}} = r_{\text{d}} G_{\text{A}} \tag{16}$$

Glutamate uptake by astrocytic transporters can instead be approximated by Michaelis-Menten kinetics [54]. Accordingly, the uptake rate reads

$$v_{\text{uptake}} = v_{\text{u}} \frac{G_{\text{A}}}{G_{\text{A}} + K_{\text{u}}} \tag{17}$$

where v_{u} is the maximal uptake rate and K_{u} is the transporters' affinity for glutamate.

Equations (15), (16) and (17) replaced in equation (8) provide a generic concise description of glutamate time course in the ESS. A further simplification though can be made based on the experimental evidence that astrocytic glutamate transporters are not saturated in physiological conditions [55]. This scenario in fact is consistent with an ESS glutamate concentration G_{A} such that $G_{\text{A}} \leq K_{\text{u}}$. Accordingly, the uptake rate can be taken roughly linear in G_{A}, that is $v_{\text{uptake}} = (v_{\text{u}}/K_{\text{u}})G_{\text{A}}$ [46], and equation (8) reduces to

$$\dot{G}_{\text{A}} \approx \sum_i G_{\text{rel}}(\tau_i) - r_{\text{d}} G_{\text{A}} - \frac{v_{\text{u}}}{K_{\text{u}}} G_{\text{A}} = \sum_i G_{\text{rel}}(\tau_i) - \Omega_{\text{c}} G_{\text{A}} \tag{18}$$

where $\Omega_{\text{c}} = r_{\text{d}} + v_{\text{u}}/K_{\text{u}}$ denotes the overall rate of glutamate clearance (Figure S5). That is, under the hypothesis that astrocytic glutamate transporters are not saturated, the time course of ESS glutamate is monoexponentially decaying roughly in agreement with experimental observations [46, 56, 57].

Equations (7) and (18) provide a description of astrocyte glutamate exocytosis and control by the astrocyte of glutamate concentration in the ESS. A key assumption in their derivation is that despite part of the released glutamate re-enters the astrocyte by uptake, the contribution of this latter to the reintegration of releasable glutamate resources therein can be neglected at first instance [58]. In other

words transporters merely function as glutamate “sinks”, so that the supply of new glutamate needed to reintegrate releasable astrocytic resources must occur through a different route, independently of extracellular glutamate [29]. While this could constitute a drastic simplification, consistency of such assumption with experimental evidence can be based on the following arguments.

Differently from nerve terminals where its reuse as transmitter is straight forward [59], uptaken glutamate in astrocytes seems to be mostly involved in the metabolic coupling with neurons [60, 61], so that glutamate supply for astrocytic exocytosis is mainly provided by other routes [58]. Glutamate sequestered by astrocytic transporters is in fact metabolized either into glutamine or α-ketoglutarate, and this latter further into lactate [58, 61]. Both glutamine and lactate are eventually exported from astrocytes to the ESS from which they may enter neurons and be reused therein as precursors for synaptic transmitter glutamate [62].

Vesicular glutamate required for astrocytic glutamate exocytosis, can be synthetized *ex novo* instead mainly from glucose imported either from intracellular glycogen, circulation [63] or from neighboring astrocytes [64] by tricarboxylic acid cycle [29]. Alternatively it can also be obtained by transamination of amino acids such as alanine, leucine or isoleucine that could be made available intracellularly [58, 65]. Although α-ketoglutarate originated from glutamate uptake could also enter the tricarboxylic acid cycle, its role in astrocyte glutamate exocytosis however remains to be elucidated [29, 66, 67].

5. Astrocyte modulation of synaptic release

Glutamate released from astrocytes can modulate synaptic transmission at nearby synapses. In the hippocampus in particular, several studies have shown that astrocyte-released glutamate modulates neurotransmitter release at excitatory synapses either towards a decrease [68-70] or an increase of it [26, 35, 71, 72]. This is likely achieved by specific activation of pre-terminal receptors, namely presynaptic glutamate receptors located far from the active zone [73]. Ultrastructural evidence indeed hints that glutamate-containing vesicles could colocalize with these receptors suggesting a focal action of astrocytic glutamate on pre-terminal receptors [35]. Such action likely occurs with a spatial precision similar to that observed at neuronal synapses [33] and is not affected by synaptic glutamate [26].

While astrocyte-induced presynaptic depression links to activation of metabotropic glutamate receptors (mGluRs) [69, 70, 74], in the case of astrocyte-induced presynaptic facilitation, ionotropic NR2B-containing NMDA receptors could also play a role [35, 72]. The precise mechanism of

action in each case remains yet unknown. The inhibitory action of presynaptic mGluRs (group II and group III) might involve a direct regulation of the synaptic release/exocytosis machinery reducing Ca^{2+} influx by inhibition of P/Q-type Ca^{2+} channels [73, 75]. Conversely, the high Ca^{2+} permeability of NR2B-containing NMDAR channels could be consistent with an increase of Ca^{2+} influx that in turn would justify facilitation of glutamate release [76, 77]. Facilitation by group I mGluRs instead [26, 71] could be triggered by ryanodine-sensitive Ca^{2+}-induced Ca^{2+} release from intracellular stores which eventually modulates presynaptic residual Ca^{2+} levels [78].

Despite the large variety of possible cellular and molecular elements involved by different receptor types, all receptors ultimately modulate the likelihood of release of synaptic vesicles [73]. From a modeling perspective equations (1-2) can be modified to include such modulation in the fraction of released glutamate (equation 3). Accordingly, three scenarios can be drawn *a priori*. (1) Activation of presynaptic glutamate receptors could modulate one or both synaptic rates Ω_d and Ω_f. (2) Modulations of release probability could be consistent instead with modulation of synaptic basal release probability U_0. (3) Alternatively release probability could be modulated making x and/or u in equations (1-2) – respectively the release probabilities of available-for-release and docked vesicles – explicitly depend on astrocyte-released glutamate (equation 18). This could be implemented for example including additional terms $\beta(G_A)$, $\gamma(G_A)$ in equations (1-2) respectively, that could mimic experimental observations.

Synaptic recovery rates Ω_d and Ω_f could indeed be modulated by presynaptic Ca^{2+} [3] and thus by modulations of Ca^{2+} concentration at the release site mediated by presynaptic glutamate receptors. Incoming action potentials though transiently affect presynaptic Ca^{2+} levels, so that this process would depend on synaptic activity. On the contrary astrocyte modulation of synaptic release is activity independent [35, 66], and this first scenario does not seem to be realistic.

Modulation of release probability of available-for-release vesicles in the third scenario would occur for example, if the pool size of readily releasable vesicles changes [79]. Although this possibility cannot be ruled out, there is no evidence that such mechanism could be mediated by presynaptic glutamate receptors [73]. Furthermore, recordings of stratum radiatum CA1 synaptic responses to Schaffer collaterals paired-pulse stimulation showed paired-pulse ratios highly stable in time during astrocyte modulation [69]. Accordingly, it could be speculated that if the interpulse interval of delivered pulse pairs in such experiments was long enough to allow replenishment of the readily-releasable pool at

those synapses, then the constancy of paired-pulse ratio would also require that the size of the readily-releasable pool is preserved. Therefore direct modulation of x is unlikely.

We thus assume that astrocytic regulation of synaptic release could be brought forth by modulations of u, the release probability of docked vesicles. In this case then, either the modulation of U_0, i.e. the synaptic basal release probability, or the addition of a supplementary term to the right hand side of equation (1) could be implemented with likely similar effects. The former scenario though seems more plausible based on the following experimental facts. First, presynaptic receptors can modulate presynaptic residual Ca^{2+} concentration by modulations of Ca^{2+} influx thereinto [73]. Second, basal residual Ca^{2+} could sensibly affect evoked synaptic release of neurotransmitter [80, 81]. Finally, third, astrocyte-modulation of synaptic release is independent of synaptic activity [35, 66, 71, 74], and so is the modulation of U_0 rather than the addition of a supplementary term $\gamma(G_A)$. Accordingly, we lump the effect of astrocytic glutamate G_A into a functional dependence of U_0 such that $U_0 = U_0(G_A)$.

The time course of extracellular glutamate is estimated in the range of few seconds [56], notwithstanding the effect of astrocytic glutamate on synaptic release could last much longer, from tens of seconds [35, 71, 72] up to minutes [70]. This hints that the functional dependence of U_0 on G_A mediated by presynaptic receptors is nontrivial. However, rather than attempting a detailed biophysical description of the complex chain of events leading from astrocytic glutamate binding of presynaptic receptors to modulation of resting presynaptic Ca^{2+} levels, we proceed in a phenomenological fashion [82]. Accordingly, we define a dynamical variable Γ that phenomenologically captures this interaction so that $U_0(G_A) = U_0(\Gamma(G_A))$. The variable Γ exponentially decays at rate Ω_G which must be small in order to mimic the long-lasting effect of astrocytic glutamate on synaptic release. On the other hand, in presence of extracellular glutamate following astrocyte exocytosis, Γ increases by $O_G G_A(1-\Gamma)$ which includes the possible saturation of presynaptic receptors by astrocytic glutamate. Accordingly, the equation for Γ reads

$$\dot{\Gamma} = O_G G_A(1-\Gamma) - \Omega_G \Gamma \qquad (19)$$

Under the hypothesis that presynaptic Ca^{2+} levels are proportional to presynaptic receptor occupancy [73], then Γ biophysically correlates with the fraction of receptor bound to astrocyte-released glutamate. The total amount of receptors that could be potentially targeted by astrocytic glutamate is assumed to be preserved in time and so are the two rate constants in equation (19). Experimental evidence however hints a more complex reality. The coverage of synapses by astrocytic processes in fact could be highly dynamic [83] and trigger repositioning of the astrocytic sites for gliotransmission release [84]. It has

been argued that this mechanism could further regulate the onset and duration of astrocyte modulation of synaptic release [33]. Nevertheless the lack of evidence in this direction allows the approximation of our description in equation (19).

The exact functional form of $U_0(\Gamma)$ depends on the nature of presynaptic receptors targeted by astrocytic glutamate. In the absence of more detailed data, we just assume that the related function $U_0(\Gamma)$ is analytic around zero and consider its first-order Taylor expansion

$$U_0(\Gamma) \cong U_0(0) + U_0^{'}(0)\Gamma \qquad (20)$$

In this framework, the expansion of order zero coincides with the synaptic basal release probability in the genuine TM model (equation 1), that is $U_0(0) = \text{const} = U_0^*$. On the contrary, the first-order term must be such that: (1) $U_0(\Gamma) \cong U_0^* + U_0^{'}(0)\Gamma$ is comprised between $[0,1]$ (being U_0 a probability); (2) for astrocyte-induced presynaptic depression, $U_0(\Gamma)$ must *decrease* with increasing Γ in agreement with the experimental observation that the more the bound receptors the stronger the inhibition of synaptic release [70]; (3) for receptor-mediated facilitation of synaptic release instead, $U_0(\Gamma)$ must *increase* with the fraction of bound receptors. Accordingly, we consider the generic expression $U_0^{'}(0) = -U_0^* + \alpha$ with $\alpha \in [0,1]$ being a parameter that lumps information on the nature of presynaptic receptors targeted by astrocytic glutamate. The resulting expression for $U_0(\Gamma)$ is then

$$U_0(\Gamma) = (1-\Gamma)U_0^* + \alpha\Gamma \qquad (21)$$

It is easy to show that all the above constraints are satisfied. The fact that $\Gamma \in [0,1]$ and $\alpha \in [0,1]$ also assures that $0 \le U_0(\Gamma) \le 1$, in agreement with the first condition. For $\alpha = 0$, it is $U_0(\Gamma) = U_0^* - \Gamma U_0^*$, so that while in absence of the astrocyte (i.e. $\Gamma = 0$), $U_0(\Gamma)$ is maximal and equals U_0^*, in presence of astrocytic glutamate (i.e. $\Gamma > 0$), $U_0(\Gamma)$ decreases by a factor ΓU_0^* consistently with a *release-decreasing* action of the astrocyte on synaptic release (second condition) (Figure S6). Conversely, if $\alpha = 1$, it is $U_0(\Gamma) = U_0^* + \Gamma(1-U_0^*)$, and in absence of the astrocyte $U_0(\Gamma = 0)$ coincides with U_0^* and is minimal while in presence of the astrocyte, U_0 increases by a factor $\Gamma(1-U_0^*)$, as expected by a *release-increasing* action of astrocytic glutamate on synaptic release (third condition) (Figure S6).

In general, for intermediate values of $0 < \alpha < 1$ astrocyte-induced decrease and increase of synaptic release coexist, mirroring the activation of different receptor types at the same synaptic terminal by astrocytic glutamate [73]. However the distinction between release-decreasing vs. release-increasing action of the astrocyte is still possible. For $0 < \alpha < U_0^*$, it is in fact $U_0(\Gamma) \le U_0^*$, so that a decrease of

synaptic release prevails on an increase of this latter. On the contrary, when $U_0^* < \alpha < 1$, it is $U_0(\Gamma) \geq U_0^*$ and increase is predominant over decrease.

Our description can be adopted in principle to study modulation of synaptic release by astrocyte-released glutamate, independently of the specific type of presynaptic receptor that is involved. This especially holds true for the case of NMDAR-mediated astrocyte-induced increase of synaptic release. While activation of such receptors at postsynaptic terminals depends on the membrane voltage for the existence of a voltage-dependent Mg^{2+} block [85, 86], this is apparently not the case for NR2B-containing presynaptic NMDA receptors targeted by the astrocyte [33]. Although the mechanism remains unknown [73], this scenario allows to use equations (19) and (21) for different receptor types which are then characterized on the mere basis of their different rates O_G and Ω_G [87].

II. Model analysis

1. Mechanisms of short-term depression and facilitation in the TM model of synaptic release

Depending on the frequency f_{in} of presynaptic spikes and the choice of values of the three synaptic parameters Ω_d, Ω_f and U_0, the TM model can mimic dynamics of both depressing and facilitating synapses [1, 8].

Presynaptic depression correlates with a decrease of probability of neurotransmitter release. Although this latter could be put forth by multiple mechanisms, the most widespread one appears to be a decrease in the release of neurotransmitter that likely reflects a depletion of the pool of ready-releasable vesicles [3]. In parallel, presynaptic depression could also be observed in concomitance of a reduction of Ca^{2+} influx into the presynaptic terminal. Such reduction is consistent with subnormal residual Ca^{2+} and thus with a reduced probability of release of docked vesicles [88]. On the contrary, synaptic facilitation is consistent with a short-term enhancement of release that correlates with increased residual Ca^{2+} concentration in the presynaptic terminal. Because, increases of residual Ca^{2+} correlate with increases of the released probability of docked vesicles [89], synaptic facilitation is therefore associated to higher release probability [3].

In the TM model, each presynaptic spike decreases the amount of glutamate available for release by RR (equation 3). The released glutamate by one spike is subsequently recovered at a rate Ω_d. Yet, if the

spike rate f_{in} is larger than the recovery rate, namely $f_{\text{in}} \geq \Omega_{\text{d}}$, progressive depletion of the pool of releasable glutamate occurs. Thus each spike will release less glutamate than the preceding one and synaptic release is progressively depressed (Figure S1A). Clearly the onset of depression is more pronounced the larger the basal release probability U_0 since, in these conditions, depletion is deeper (Figure S1B).

Immediately after each presynaptic spike, the release probability is augmented by a factor $U_0(1-u)$ and following recovers to its original baseline value U_0 at rate Ω_{f}. If the spike rate is larger than Ω_{f} though, i.e. $f_{\text{in}} \geq \Omega_{\text{f}}$, the release probability progressively grows with incoming spikes, and facilitation occurs (Figure S1C). One though should keep in mind that facilitating and depressing mechanisms are intricately interconnected and stronger facilitation leads to higher u values which in turn leads to stronger depression. Accordingly, facilitating presynaptic spike trains, namely spike trains that are characterized by $f_{\text{in}} \geq \Omega_{\text{f}}$, eventually bring forth depression of synaptic release if they last sufficiently long (Figure S1D).

2. Characterization of paired-pulse depression and facilitation

Short-term depression and facilitation can be characterized by paired-pulse stimulation [90]. Consider the pair of neurotransmitter release events, labeled by RR_1 and RR_2, triggered by a pair of presynaptic spikes. Then, the paired-pulse ratio (PPR) is defined as $\text{PPR} = RR_2 / RR_1$ and can be used to discriminate between short-term paired-pulse depression (PPD) and/or facilitation (PPF) displayed by the synapse under consideration [3]. Indeed, if the paired-pulse stimulus is delivered to the synapse at rest, then PPR values larger than 1 imply that the amount of resources released by the second spike in the pair is larger than the one due to the first spike, i.e. $RR_2 > RR_1$, thus synaptic release is facilitated or equivalently, PPF occurs. Conversely, if $\text{PPR} < 1$ then $RR_2 < RR_1$ which marks the occurrence of PPD (Figure S2A).

When trains of presynaptic spikes in any sequence are considered instead, the above scenario is complicated by the fact that for each i-th pair of consecutive spikes in the train, the value of $\text{PPR}_i = RR_i / RR_{i-1}$ depends on the past activity of the synapse. In this context in fact the released resources RR_{i-1} at the (i-1)-th spike are dictated by the state of the synapse upon arrival of the (i-1)-th spike which in turn depends on the previous spikes in the train. Accordingly, values of $\text{PPR}_i > 1$ ($\text{PPR}_i < 1$)

are not any longer a sufficient condition to discriminate between PPD and PPF. This concept can be elucidated considering the difference of released resources ΔRR_i associated with PPR_i, namely:

$$\begin{aligned}\Delta RR_i &= RR_i - RR_{i-1} \\ &= u_i x_i - u_{i-1} x_{i-1} \\ &= (u_i - u_{i-1}) x_i + u_{i-1}(x_i - x_{i-1}) \\ &= x_i \Delta u_i + u_{i-1} \Delta x_i\end{aligned} \qquad (22)$$

where $u_i = u(t_i^+)$ and $x_i = x(t_i^-)$ (equation 3). According to definition of PPF, one would expect to measure $PPR_i > 1$ and thus $\Delta RR_i > 0$ merely when the probability of release of docked vesicles has increased from one spike to the following one, that is when $\Delta u_i > 0$ [3]. Nonetheless, equation (22) predicts that $\Delta RR_i > 0$ (thus $PPR_i > 1$) can also be found when $\Delta u_i < 0$, if in between spikes, sufficient synaptic resources are *recovered*, that is if $\Delta x_i > 0$. This situation occurs whenever $|\Delta u_i| / u_{i-1} < \Delta x_i / x_i$ (equation 22) and corresponds to the mechanism of synaptic plasticity dubbed as “recovery from depletion”, a further mechanisms of short-term synaptic plasticity that is different from both PPF and PPD [91] (Figure S2B-C).

For the purpose of our analysis nevertheless, distinction between facilitation (or PPF) and recovery was observed to be redundant in the characterization of astrocyte modulations of paired-pulse plasticity in most of the studied cases (results not shown). Accordingly, PPD and PPF were distinguished on the mere basis of their associated PPR value: PPD when PPR < 1 and PPF when PPR > 1 (Figures 5-7, 9, S10, S11). The only exception to this rationale was found when release-increasing astrocytes regulate neurotransmitter release from depressing synapses. Here, subtler changes of paired-pulse plasticity by astrocytic glutamate required the mechanism of recovery to be taken into account too (Figure S11).

3. Mean-field description of synaptic release

An advantage of the TM model is that it can be used to derive a mean-field description of the average synaptic dynamics in responses to many different inputs sharing the same statistics without having to solve an equally large number of equations [8, 92]. The derivation of such description, originally developed for the mean-field dynamics of large neural populations [8], is following outlined.

The first step in the derivation is to rewrite the equation for u (equation 1) in terms of $u(t_i^+)$, since this latter value, namely the value of u immediately after the arrival of a spike at t_i, is the one that appears in equation (2). With this regard, we note that [92]:

$$u(t_i^+) = u + U_0(1-u)$$
$$\Rightarrow u = \frac{u(t_i^+) - U_0}{1 - U_0} \quad (23)$$

Accordingly, substituting this latter into equation (1) and redefining u hereafter as $u \leftarrow u(t_i^+)$, we obtain:

$$\dot{u} = \Omega_{\mathrm{f}}(U_0 - u) + U_0 \sum_i (1-u)\delta(t - t_i) \quad (24)$$

In this fashion, we can update x and u simultaneously at each spike, rather than first compute u and then update x as otherwise required by equations (1-2).

Consider then N presynaptic spike trains of same duration delivered to the synapse at identical initial conditions. The trial-averaged synaptic dynamics is described by equations (1-2), in terms of the mean quantities $\bar{u} = 1/N \sum_k^N u_k$ and $\bar{x} = 1/N \sum_k^N x_k$. That is

$$\dot{\bar{x}} = \Omega_{\mathrm{d}}(1-\bar{x}) - \frac{1}{N}\sum_k^N \sum_i ux\delta(t - t_{ik}) \quad (25)$$

$$\dot{\bar{u}} = \Omega_{\mathrm{f}}(U_0 - \bar{u}) + \frac{U_0}{N}\sum_k^N \sum_i (1-u)\delta(t - t_{ik}) \quad (26)$$

Focusing on a time interval Δt, the above equations can be rewritten by their equivalent difference form

$$\bar{x}(t+\Delta t) - \bar{x}(t) = \Omega_{\mathrm{d}}(1-\bar{x}(t))\Delta t - \frac{1}{N}\sum_k^N u(t)x(t)\Delta_k(\Delta t) \quad (27)$$

$$\bar{u}(t+\Delta t) - \bar{u}(t) = \Omega_{\mathrm{f}}(U_0 - \bar{u}(t))\Delta t + \frac{U_0}{N}\sum_k^N (1-u(t))\Delta_k(\Delta t) \quad (28)$$

where $\Delta_k(\Delta t)$ is the number of spikes per time interval Δt for the k-th trial and is a strongly fluctuating (stochastic) quantity itself.

Analysis of neurophysiological data revealed that individual neurons in vivo fire irregularly at all rates reminiscent of the so-called Poisson process [93]. Mathematically, the Poisson assumption means that at each moment, the probability that a neuron will fire is given by the value of the instantaneous firing rate and is independent of the timing of previous spikes. We thus take the N spike trains under consideration to be different realizations of the same Poisson process of average frequency $f_{\mathrm{in}}(t)$ and we average in time over Δt equations (27-28) (denoted by "$\langle\ \rangle$"). Thanks to the Poisson hypothesis, the variables $u(t)$, $u(t)x(t)$ and $\Delta_k(\Delta t)$ can be considered independent and thus averaged independently.

Therefore, taking Δt of the order of several interspike intervals and shorter than the longest time scale in the system between $1/\Omega_d$ and $1/\Omega_f$ [94], we note that the time average of $\Delta_k(\Delta t)$ can be estimated by $\langle \Delta_k(\Delta t) \rangle = f_{in} \Delta t$. Accordingly,

$$\langle \bar{x}(t+\Delta t) \rangle - \langle \bar{x}(t) \rangle = \Omega_d (1 - \langle \bar{x}(t) \rangle) \Delta t - \frac{1}{N} \sum_k^N \langle u(t) x(t) \rangle f_{in} \Delta t \tag{29}$$

$$\langle \bar{u}(t+\Delta t) \rangle - \langle \bar{u}(t) \rangle = \Omega_f (U_0 - \bar{u}(t)) \Delta t + \frac{U_0}{N} \sum_k^N (1 - \langle u(t) \rangle) f_{in} \Delta t \tag{30}$$

Finally, dividing by Δt the two equations above yields

$$\langle \dot{\bar{x}} \rangle = \Omega_d (1 - \langle \bar{x} \rangle) - \langle \bar{u} \rangle \langle \bar{x} \rangle f_{in} \tag{31}$$

$$\langle \dot{\bar{u}} \rangle = \Omega_f (U_0 - \langle \bar{u} \rangle) + U_0 (1 - \langle \bar{u} \rangle) f_{in} \tag{32}$$

where we made the approximation that $1/N \sum_k^N \langle ux \rangle = \langle \bar{u} \rangle \langle \bar{x} \rangle$. This would be possible only if $u(t)$ and $x(t)$ were statistically independent while they are not, as both are functions of the same presynaptic spikes. The relative error of this approximation can be estimated using the Cauchy-Schwarz inequality of the probability theory [8]:

$$\frac{|\langle ux \rangle - \langle u \rangle \langle x \rangle|}{\langle u \rangle \langle x \rangle} \le c_u c_x \tag{33}$$

where c_u and c_x stand for the coefficient of variation of the random variables u and x respectively and satisfy the following

$$c_u^2 = \frac{(1 - U_0) f_{in}}{(\Omega_f + f_{in})^2 (2\Omega_f + U_0 (2 - U_0) f_{in})} \tag{34}$$

$$c_x^2 = \frac{(1 + c_u^2) \langle u \rangle f_{in}}{2\Omega_d + \langle u \rangle (1 - \langle u \rangle (1 + c_u^2)) f_{in}} \tag{35}$$

Accordingly, the self-consistency of the mean-field theory can be checked plotting the product $c_u c_x$ as a function of the frequency f_{in} of the presynaptic spikes and the synaptic basal release probability U_0 (Figure S7A). For the cases considered in our study, the error does not exceed 10%.

4. Frequency response and limiting frequency of a synapse

For the sake of clarity we will following denote by capital letters the mean quantities, hence $U = \langle \bar{u} \rangle$ and $X = \langle \bar{x} \rangle$. One of the advantages of the mean-field description derived above is the possibility to obtain an analytical expression for the mean amount of released resources as a function of the average input frequency. At steady state in fact, that is for $\dot{U} = \dot{X} = 0$, equations (31-32) can be solved for the equivalent steady state values (denoted by the subscript '∞'):

$$U_\infty = \frac{U_0(\Omega_\mathrm{f} + f_\mathrm{in})}{(\Omega_\mathrm{f} + U_0 f_\mathrm{in})} \tag{36}$$

$$X_\infty = \frac{\Omega_\mathrm{d}}{\Omega_\mathrm{d} + U_\infty f_\mathrm{in}} \tag{37}$$

Accordingly, the mean steady-state released synaptic resources RR_∞ are given by (equation 3):

$$RR_\infty = U_\infty X_\infty = \frac{U_0 \Omega_\mathrm{d}(\Omega_\mathrm{f} + f_\mathrm{in})}{\Omega_\mathrm{d}\Omega_\mathrm{f} + U_0(\Omega_\mathrm{d} + \Omega_\mathrm{f}) f_\mathrm{in} + U_0 f_\mathrm{in}^2} \tag{38}$$

The above equation provides the *frequency response* of the synapse in steady-state conditions.

The slope of the frequency response curve, that is $RR_\infty^{'} = \lim_{\Delta f_\mathrm{in} \to 0} \Delta RR_\infty / \Delta f_\mathrm{in}$, can be used to distinguish between facilitating and depressing synapses. Indeed a negative slope implies that $\Delta RR_\infty < 0$ for increasing frequencies, which marks the occurrence of depression. Conversely, a positive slope value is when $\Delta RR_\infty > 0$ for $\Delta f_\mathrm{in} > 0$, which reflects ongoing facilitation (Section II.2). Notably, for vanishing input frequencies, that is for $f_\mathrm{in} \to 0$, the slope of the frequency response is $RR_\infty^{'}(f_\mathrm{in} \to 0) = ((1 - U_0)\Omega_\mathrm{d} - U_0 \Omega_\mathrm{f}) / (\Omega_\mathrm{d}\Omega_\mathrm{f})^2$, which can be either positive or negative depending on the sign of the numerator. With this regard, a threshold value of U_0, i.e. U_thr, can be defined as

$$U_\mathrm{thr} = \frac{\Omega_\mathrm{d}}{\Omega_\mathrm{d} + \Omega_\mathrm{f}} \tag{39}$$

so that if $U_0 < U_\mathrm{thr}$ a synapse can be facilitating, if not, that is if $U_0 > U_\mathrm{thr}$, the synapse is depressing (Figure S3A).

The frequency response of a depressing synapse is thus maximal for $f_\mathrm{in} \to 0$, for which $RR_\infty \cong U_0$, and then monotonously decreases towards zero for increasing frequency. In this case, a cut-off

frequency can be defined, beyond which the onset of depression is marked by a strong attenuation with respect to the maximal RR_∞, that is an attenuation larger than 3 dB. For a facilitating synapse instead, the frequency response is nonmonotonic and bell-shaped and a peak frequency f_{peak} can be recognized, at which RR_∞ is maximal. This follows from the coexistence of facilitation and depression by means of which the stronger facilitation, the stronger depression. That is, for $0 < f_{\mathrm{in}} < f_{\mathrm{peak}}$, the slope of the frequency response is positive and thus facilitation occurs. For increasing frequencies on the other hand, the increase of facilitation is accompanied by growing depression, up to $f_{\mathrm{in}} = f_{\mathrm{peak}}$ when the two compensate, and afterwards depression takes over facilitation for $f_{\mathrm{in}} > f_{\mathrm{peak}}$.

Both the cut-off frequency and the peak frequency can be regarded as the *limiting frequency* f_{lim} of the synapse for the onset of depression. Accordingly, f_{lim} can be obtained from equation (38) and reads

$$f_{\mathrm{lim}} = \begin{cases} \Omega_{\mathrm{f}}\left(\sqrt{\dfrac{\Omega_{\mathrm{d}}}{\Omega_{\mathrm{f}}}\left(\dfrac{1-U_0}{U_0}\right)}-1\right) & \text{if } U_0 < U_{\mathrm{thr}} \\ \dfrac{\Omega_{\mathrm{d}}}{\left(1+\sqrt{2}\right)U_0} & \text{if } U_0 > U_{\mathrm{thr}} \end{cases} \qquad (40)$$

The corresponding value RR_{lim} of steady-state average released synaptic resources is obtained replacing f_{in} by f_{lim} in equation (38) (Figure S3B):

$$RR_{\mathrm{lim}} = \begin{cases} \dfrac{\Omega_{\mathrm{d}}U_0}{U_0\left(\Omega_{\mathrm{d}}-\Omega_{\mathrm{f}}\right)+2U_0\left(1-U_0\right)\Omega_{\mathrm{d}}\Omega_{\mathrm{f}}} & \text{if } U_0 < U_{\mathrm{thr}} \\ \dfrac{\Omega_{\mathrm{d}}U_0}{\sqrt{2}} & \text{if } U_0 > U_{\mathrm{thr}} \end{cases} \qquad (41)$$

From the above analysis therefore, it follows that two are the conditions needed for a synapse to exhibit facilitation. These are: (1) $U_0 < U_{\mathrm{thr}}$, and (2) $f_{\mathrm{in}} < f_{\mathrm{lim}}$. Alternatively, if we are able to estimate the slope of the frequency response curve for a given input frequency, then it is necessary and sufficient for the occurrence of facilitation that $\lim_{\Delta f_{\mathrm{in}}\to 0} \Delta RR_\infty / \Delta f_{\mathrm{in}} > 0$ (Figure S9).

5. Mean-field description of astrocyte-to-synapse interaction

We can extend the mean-field description of synaptic release to include modulation of this latter by astrocytic glutamate. The difference with respect to the mean-field description of synaptic release (equations 31-32) is that, when the astrocyte is taken into account, the synaptic basal release probability U_0 changes in time according to equation (21). Notwithstanding, in the case that

astrocytic Ca^{2+} dynamics and synaptic glutamate release are statistically independent (see "The road map of astrocyte regulation of presynaptic short-term plasticity" in "Methods" in the main text), equation (32) can be rewritten as following:

$$\left\langle \dot{\bar{u}} \right\rangle = \Omega_{\mathrm{f}} \left(\left\langle \bar{U}_0 \right\rangle - \left\langle \bar{u} \right\rangle \right) + \left\langle \bar{U}_0 \right\rangle \left(1 - \left\langle \bar{u} \right\rangle \right) f_{\mathrm{in}} \quad (42)$$

The mean basal probability of synaptic release ultimately depends on the frequency of glutamate exocytosis from the astrocyte. In order to seek an average description of this latter though, we need that at each moment the probability of glutamate exocytosis is independent of the timing of the previous release event, namely that glutamate exocytosis from the astrocyte is a Poisson process [94]. Indeed recent studies provide support to this scenario, hinting that the period of spontaneous astrocytic Ca^{2+} oscillations could be reminiscent of a Poisson process [95, 96]. Moreover, glutamate exocytosis from astrocytes is reported to occur for Ca^{2+} concentrations as low as 200 nM [27], that is lower than the average reported minimal peak Ca^{2+} concentration of 200 – 250 nM [28, 97]. This allows to assume that the majority of Ca^{2+} oscillations triggers glutamate exocytosis from the astrocyte. Accordingly, the inter-event intervals between two consecutive glutamate release events, can be also assumed to be Poisson distributed. This allows averaging of equation (7) to obtain

$$\left\langle \dot{\bar{x}}_{\mathrm{A}} \right\rangle = \Omega_{\mathrm{A}} \left(1 - \left\langle \bar{x}_{\mathrm{A}} \right\rangle \right) - U_{\mathrm{A}} \left\langle \bar{x}_{\mathrm{A}} \right\rangle f_{\mathrm{C}} \quad (43)$$

where f_{C} denotes the frequency of exocytosis-triggering astrocytic Ca^{2+} oscillations. Similarly, we can do averaging of equation (18) to obtain a mean-field description of glutamate concentration in the ESS, that is

$$\left\langle \dot{\bar{G}}_{\mathrm{A}} \right\rangle = -\Omega_{\mathrm{c}} \left\langle \bar{G}_{\mathrm{A}} \right\rangle + \beta U_{\mathrm{A}} \left\langle \bar{x}_{\mathrm{A}} \right\rangle f_{\mathrm{C}} \quad (44)$$

where $\beta = \rho_{\mathrm{A}} n_{\mathrm{v}} G_{\mathrm{v}}$. Since the time course of glutamate in the ESS can be assumed to be much faster than the duration of astrocyte effect on synaptic release [35, 47] we can take into account only those processes of glutamate time course that are slower than the overall clearance rate Ω_{c}. Equation (44) above, thus simplifies to

$$\left\langle \bar{G}_{\mathrm{A}} \right\rangle = \frac{\beta}{\Omega_{\mathrm{c}}} U_{\mathrm{A}} \left\langle \bar{x}_{\mathrm{A}} \right\rangle f_{\mathrm{C}} \quad (45)$$

Averaging of equation (19) then provides

$$\left\langle \dot{\bar{\Gamma}} \right\rangle = -\Omega_{\mathrm{G}} \left\langle \bar{\Gamma} \right\rangle + O_{\mathrm{G}} \left\langle \bar{G}_{\mathrm{A}} \right\rangle \left(1 - \left\langle \bar{\Gamma} \right\rangle \right) \quad (46)$$

where we have made the approximation that $\left\langle \overline{G_{\mathrm{A}}(1-\Gamma)} \right\rangle = \left\langle \overline{G}_{\mathrm{A}} \right\rangle \left(1-\left\langle \overline{\Gamma} \right\rangle\right)$. Substituting $\left\langle \overline{G}_{\mathrm{A}} \right\rangle$ in equation (45) into equation (46), allows to express the fraction of bound receptors Γ as a function of the astrocyte-released glutamate, which is ultimately dependent on the exocytosis frequency f_{C} (equation 43). Therefore

$$\left\langle \dot{\overline{\Gamma}} \right\rangle = -\Omega_{\mathrm{G}} \left\langle \overline{\Gamma} \right\rangle + \beta \frac{O_{\mathrm{G}}}{\Omega_{\mathrm{c}}} U_{\mathrm{A}} \left\langle \overline{x}_{\mathrm{A}} \right\rangle \left(1-\left\langle \overline{\Gamma} \right\rangle\right) f_{\mathrm{C}} \tag{47}$$

Hence, at steady-state

$$X_{\mathrm{A}\infty} = \frac{\Omega_{\mathrm{A}}}{\Omega_{\mathrm{A}} + U_{\mathrm{A}} f_{\mathrm{C}}} \tag{48}$$

$$\Gamma_{\infty} = \frac{\beta O_{\mathrm{G}} U_{\mathrm{A}} X_{\mathrm{A}\infty} f_{\mathrm{C}}}{\Omega_{\mathrm{c}}\Omega_{\mathrm{G}} + \beta O_{\mathrm{G}} U_{\mathrm{A}} X_{\mathrm{A}\infty} f_{\mathrm{C}}} = \frac{\beta \Omega_{\mathrm{A}} O_{\mathrm{G}} U_{\mathrm{A}} f_{\mathrm{C}}}{\Omega_{\mathrm{A}}\Omega_{\mathrm{c}}\Omega_{\mathrm{G}} + \left(\Omega_{\mathrm{c}}\Omega_{\mathrm{G}} + \beta\Omega_{\mathrm{A}} O_{\mathrm{G}}\right) U_{\mathrm{A}} f_{\mathrm{C}}} \tag{49}$$

We can eventually substitute equation (49) in (42) to obtain an analytical expression of synaptic basal release probability as function of the frequency of astrocyte exocytosis. That is

$$U_{0\infty} = \frac{\Omega_{\mathrm{A}}\Omega_{\mathrm{c}}\Omega_{\mathrm{G}} U_0^* + \left(\Omega_{\mathrm{c}}\Omega_{\mathrm{G}} U_0^* + \alpha\beta\Omega_{\mathrm{A}} O_{\mathrm{G}}\right) U_{\mathrm{A}} f_{\mathrm{C}}}{\Omega_{\mathrm{A}}\Omega_{\mathrm{c}}\Omega_{\mathrm{G}} + \left(\Omega_{\mathrm{c}}\Omega_{\mathrm{G}} + \beta\Omega_{\mathrm{A}} O_{\mathrm{G}}\right) U_{\mathrm{A}} f_{\mathrm{C}}} \tag{50}$$

The relative error of this approximation can be estimated by the Cauchy-Schwarz inequality of the probability theory as pointed out in Section II.3 (equation 33):

$$\frac{\left|\left\langle x_{\mathrm{A}}\Gamma \right\rangle - \left\langle x_{\mathrm{A}} \right\rangle \left\langle \Gamma \right\rangle\right|}{\left\langle x_{\mathrm{A}} \right\rangle \left\langle \Gamma \right\rangle} \leq c_{x_{\mathrm{A}}} c_{\Gamma} \tag{51}$$

where $c_{x_{\mathrm{A}}}$ and c_{Γ} stand for the coefficients of variation of the random variables x_{A} and Γ respectively and satisfy the following:

$$c_{x_{\mathrm{A}}}^2 = \frac{U_{\mathrm{A}}^2 f_{\mathrm{C}}}{2\left(\Omega_{\mathrm{A}} + f_{\mathrm{C}}\left(1-U_{\mathrm{A}}\right)U_{\mathrm{A}}/2\right)} \tag{52}$$

$$c_{\Gamma}^2 \approx \frac{(1-\vartheta)(2\Omega_{\mathrm{G}} + f_{\mathrm{C}})\left((1-\vartheta)\Omega_{\mathrm{G}} + f_{\mathrm{C}}\left(1+\left(2\tilde{\Gamma}-1\right)\vartheta\right)\right)}{(\Omega_{\mathrm{G}} + f_{\mathrm{C}})\left(2\Omega_{\mathrm{G}} + f_{\mathrm{C}}\left(1-\vartheta^2\right)\right)\tilde{\Gamma}^2} - 1 \tag{53}$$

with $\tilde{\Gamma} = 1 + \vartheta\Omega_{\mathrm{G}} / \left((\vartheta-1) f_{\mathrm{C}} - \Omega_{\mathrm{G}}\right)$ and $\vartheta = \exp\left(-O_{\mathrm{G}}\beta U_{\mathrm{A}}^2/\Omega_{\mathrm{c}}\right)$ (Appendix B). For the cases considered in our study, the error does not exceed ~7% (Figure S7B).

Appendix A: Model equations

1. Astrocyte Ca^{2+} dynamics

1.1. Conditioning IP_3 signal

$$m(t) = m_0 + k\sqrt{IP_3(t) - I_b}$$

with:

- m_0, constant component of the IP_3 signal (in normalized units);
- k, scaling factor;
- $IP_3(t)$, externally driven IP_3 signal (in normalized units);
- I_b, IP_3 threshold for CICR (in normalized units).

1.2. Calcium signal:

$$C(t) = C_0 + \lambda_{\mathrm{AM}} m_{\mathrm{AM}}(t) \sin^w\left(2\pi \cdot \lambda_{\mathrm{FM}} m_{\mathrm{FM}}(t) \cdot f_{\mathrm{C}} t + \varphi_{\mathrm{C}}\right)$$

with:

- C_0, constant component of the Ca^{2+} signal (in normalized units);
- λ_{AM}, λ_{FM}, binary parameters that equal to 1 if AM and/or FM encoding features are taken into account in the astrocyte Ca^{2+} dynamics;
- f_C, frequency of Ca^{2+} oscillations;
- φ_C, phase of Ca^{2+} oscillations;
- w, shape factor (it must be a positive even integer).

2. Astrocytic glutamate release

2.1. Glutamate exocytosis

$$\dot{x}_{\mathrm{A}} = \Omega_{\mathrm{A}}(1 - x_{\mathrm{A}}) - U_{\mathrm{A}} \sum_i x_{\mathrm{A}}\, \delta(t - \tau_i)$$

with:

- τ_i, instants of glutamate release from the astrocyte, such that $\exists \tau_i : C(\tau_i) = C_{\mathrm{thr}} \wedge \dot{C}(\tau_i) > 0$;
- x_A, fraction of astrocytic glutamate vesicles available for release;
- U_A, basal release probability of astrocytic glutamate vesicles;
- Ω_A, recovery rate of released astrocyte glutamate vesicles.

2.2. Glutamate time course in the extracellular space

$$G_{\mathrm{rel}}(\tau_i) = \rho_A n_v G_v \cdot U_A x_A(\tau_i^-)$$

$$\dot{G}_{\mathrm{A}} = \sum_i G_{\mathrm{rel}}(\tau_i) - v_{\mathrm{u}} \frac{G_{\mathrm{A}}}{G_{\mathrm{A}} + K_{\mathrm{u}}} - r_{\mathrm{d}} G_{\mathrm{A}}$$

This latter equation is implemented as

$$\dot{G}_{\mathrm{A}} = \sum_i G_{\mathrm{rel}}(\tau_i) - \Omega_{\mathrm{c}} G_{\mathrm{A}}$$

where $\Omega_{\mathrm{c}} = r_{\mathrm{d}} + v_{\mathrm{u}}/K_{\mathrm{u}}$.

In the above, it is:

- G_{rel}, glutamate released from the astrocyte into the ESS;
- G_{A}, glutamate concentration in the ESS;
- G_{v}, glutamate concentration within astrocytic vesicles;
- n_{v}, number of ready-releasable astrocytic vesicles;
- ρ_{A}, ratio between the average volume of astrocytic vesicles and the ESS volume;
- r_{d}, glutamate clearance rate in the ESS by diffusion;
- v_{u}, maximal glutamate uptake rate by transporters;
- K_{u}, transporters' affinity for glutamate;
- Ω_{c}, glutamate clearance rate in the ESS.

3. Presynaptic receptors

$$\dot{\Gamma} = O_{\mathrm{G}} G_{\mathrm{A}} (1-\Gamma) - \Omega_{\mathrm{G}} \Gamma$$

with:

- Γ, fraction of activated presynaptic receptors;
- O_{G}, onset rate of astrocyte modulation of synaptic release probability;
- Ω_{G}, recovery rate of astrocyte modulation of synaptic release probability.

4. Synaptic release

$$U_0 = (1-\Gamma) U_0^* + \alpha \Gamma$$

$$\dot{x} = \Omega_{\mathrm{d}} (1-x) - \sum_i u x \delta(t - t_i)$$

$$\dot{u} = \Omega_{\mathrm{f}} (U_0 - u) + U_0 \sum_i (1-u) \delta(t - t_i)$$

with:

– U_0, basal synaptic release probability function;

– x, fraction of synaptic glutamate vesicles available for release;

– u, per-spike usage of available glutamate vesicles;

– t_i, instant of synaptic release upon arrival of the i-th action potential;

– U_0*, basal synaptic release probability (i.e. without astrocyte);

– α, "effect parameter" of astrocyte regulation of synaptic release;

– Ω_d, recovery rate of released synaptic glutamate vesicles;

– Ω_f, rate of synaptic facilitation.

Appendix B: Estimation of the coefficient of variation of Γ

In order to estimate the coefficient of variation of Γ we need a recursive expression of the peak value Γ_n associated to *n*-th glutamate release event from the astrocyte. This can be done by solving equation (19) for Γ(t), nonetheless some approximations can be done in our case to make the solution analytically tractable.

We start from the observation that the frequency of Ca^{2+} oscillations in astrocytes can be assumed to be much lower than the rate of replenishment of astrocytic glutamate resources, i.e. $f_C << \Omega_A$ [37, 41]. As discussed in the section "Persistent Ca^{2+} oscillations in astrocytes can regulate presynaptic short-term plasticity" of "Results", it follows that astrocytic glutamate exocytosis can be described by quantal release events of almost identical magnitude roughly equal to $RR_{An} = U_A x_{An} \approx U_A^2$. Accordingly the time course of glutamate released from the astrocyte by the *n*-th exocytotic event occurring at $t = \tau_n$, is (equation 18):

$$G_A(t \geq \tau_n) = \beta \cdot RR_{An} \exp(-\Omega_c t) = \beta U_A^2 \exp(-\Omega_c t) \tag{A1}$$

Experimental evidences also suggest that the onset of astrocyte effect on synaptic release and the rate of glutamate degradation in the extrasynaptic space are much faster than the recovery rate from astrocyte modulation, i.e. $\Omega_G << \Omega_c, O_G G_A$ (see Appendix C). Thus we can assume that at its onset till it reaches its peak value Γ_n at $t = \hat{t}_n$, astrocyte modulation is set mainly by the time course of release glutamate and the binding of this latter to such receptors. Accordingly, for $\tau_n \leq t \leq \hat{t}_n$, equation (19) can be simplified into

$$\dot{\Gamma} \cong O_G G_A (1 - \Gamma) \tag{A2}$$

where G_A is given by equation (A1). On the other hand, once glutamate in the extrasynaptic space is cleared, astrocyte modulation monoexponentially decays from its peak value at rate Ω_G till the next glutamate release from the astrocyte (assumed to occur at $t = \tau_{n+1} = \tau_n + \Delta t$). Namely for $\hat{t}_n \leq t \leq \tau_n + \Delta t$ it is

$$\Gamma(t) = \Gamma_n \exp(-\Omega_G t) \tag{A3}$$

Since $f_C \ll \Omega_A \ll \Omega_c, O_G G_A$ (Appendix C) we can assume that the whole glutamate released by the *n*-th release event at $t = \tau_n$ is cleared before the following exocytotic event. Thus solving equation (A2) with the initial condition $\Gamma(t = \tau_{n+1}) = \Gamma_n \exp(-\Omega_G \Delta t)$, provides an iterative expression for Γ_n such as

$$\Gamma_{n+1} = \vartheta \cdot \Gamma_n \exp(-\Omega_G \Delta t) + 1 - \vartheta \tag{A4}$$

with $\vartheta = \exp\left(-O_G \beta U_A^2 / \Omega_c\right)$. Assuming steady-state conditions, i.e. $\Gamma_{n+1} \cong \Gamma_n$, equation (A4) can be used to estimate $\langle \Gamma \rangle$ and $\langle \Gamma^2 \rangle$ that are needed to eventually compute c_Γ, the coefficient of variation of Γ.

The average value of the exponential decay factor in equation (A4) is the integral over all positive Δ*t* values of $\exp(-\Omega_G \Delta t)$ times the probability density for a Poisson train of glutamate exocytotic events occurring at rate f_C and that produce an inter-event interval of duration Δ*t* (see "Persistent Ca^{2+} oscillations in astrocytes can regulate presynaptic short-term plasticity" of "Results" in the main text). Recall that inter-event intervals of a Poisson distribution are exponentially distributed so that the probability of occurrence of a inter-event interval of duration Δ*t* is $f_C \exp(-f_C \Delta t)$. Thus, the average exponential decrement is

$$\langle \exp(-\Omega_G \Delta t) \rangle = f_C \int_0^\infty \exp(-\Omega_G \Delta t) \exp(-f_C \Delta t) d\Delta t = \frac{f_C}{\Omega_G + f_C} \tag{A5}$$

In order for Γ to return on average to its steady-state value between glutamate release events, we must therefore require that

$$\langle \Gamma \rangle = \tilde{\Gamma} = \frac{(\vartheta - 1)(\Omega_G + f_C)}{f_C(\vartheta - 1) + \Omega_G} \tag{A6}$$

Averaging over the square of Γ_n as given by equation (A4) provides $\langle \Gamma^2 \rangle$ and c_Γ can be thus computed accordingly. Comparison of equation (A6) with equation (49) (Figure S8) shows that the error introduced by the above rationale in the computation of c_Γ is roughly up to ~10% within the frequency range, i.e. 0.01–1 Hz, of Ca^{2+} oscillations considered in this study.

Appendix C: Parameter estimation

Synaptic parameters. Single hippocampal boutons normally release at most a single quantum of neurotransmitter [98, 99]. Accordingly, reported release probabilities (U_0*) for these synapses are small, generally comprised between ~0.09 [52] and ~0.6 [98] with average values between ~0.3 – 0.55 [71]. Notwithstanding there could also be specific synapses that exhibit probabilities ranging <0.05 – 0.9 [100]. In general, facilitating hippocampal synapses are found with lower (basal) release probability [100].

Vesicles in the readily releasable pool preferentially undergo rapid endocytosis, typically occurring within 1 – 2 s (i.e. Ω_d = 0.5 – 1 Hz) [101]. However, vesicle recycling could be as fast as 10 – 20 ms [98, 102], implying a maximum recovery rate of Ω_d = 50 – 100 Hz. Facilitation rates (Ω_f) can be estimated by the decay time of intracellular Ca^{2+} increases at presynaptic terminals following action potential arrival [103, 104]. Accordingly, typical decay times for Ca^{2+} transients are reported to be <500 ms [103] with an upper bound between 0.65 – 2 s [104]. Such Ca^{2+} transients though shall be taken as upper limit of Ca^{2+} level decay due to the high affinity of the Ca^{2+} indicator used to image them [105, 106]. Therefore, estimated facilitation rates can be as low as Ω_f = 0.5 Hz and range up to 2 Hz [90] or beyond [104].

Astrocytic calcium dynamics. For the purposes of our study both Ca^{2+} and IP_3 signals in equations (4-6) can be assumed to be normalized with respect to their maxima. Furthermore, because glutamate exocytosis from astrocytes likely occurs in concomitance only with Ca^{2+} increases above basal Ca^{2+} concentration [26, 27, 107], we can take C_0 and I_b equal to 0. In this fashion both $C(t)$ and $IP_3(t)$ in equations (4-6) vary within 0 and 1.

The threshold Ca^{2+} concentration (C_{thr}) of glutamate exocytosis in astrocytes is estimated to be between ~125 nM [27] and ~850 nM [28]. Given that in stimulated astrocytes, peak Ca^{2+} concentration could reach 1 μM or beyond [27], these values suggest at most the range of ~0.13 – 0.8 for C_{thr} in our model. Finally, reported values for the frequency of evoked Ca^{2+} oscillations (f_C) in astrocytes can be as low as ~0.01 Hz [97, 108] and range up to 0.1 Hz [18]. In our description we assume that maximal amplitude or frequency of Ca^{2+} oscillations correspond to maximal stimulus, i.e. IP_3 = 1. Accordingly, we take k = 1.

Astrocytic glutamate exocytosis. Exocytosis of glutamate from astrocytes is seen to occur more readily at processes than at cell bodies [39, 109]. Vesicles observed in astrocytic processes have regular (spherical) shape with typical diameters (d_v) between 27.6 ± 12.3 nm [30] and 110 nm [39]. Accordingly, vesicular volume V_v ranges between ~2 – 700 $\cdot 10^{-21}$ dm^3. Vesicular glutamate content is approximately the same or at most as low as one third of synaptic vesicles at adjacent nerve terminals [35, 37]. Given that glutamate concentration in synaptic vesicles is estimated between ~60 – 150 mM [58], then astrocytic vesicular glutamate (G_v) likely is in the range of ~20 – 150 mM.

The majority of glutamate vesicles at astrocytic processes clusters in close proximity to the plasma membrane, i.e. <100 nm, but about half of them is found within a distance of 40 – 60 nm, suggesting the presence of 'docked' vesicles in the astrocytic process [35]. Borrowing the synaptic rationale that docked vesicles corresponds approximately to readily releasable ones [52], then the average number of glutamate vesicles available for release (n_v) could be between ~1 – 6 [35]. Furthermore, because release probability is proportional to the number of docked vesicles [52], and such docked vesicles approximately correspond to 13% of vesicles at astrocytic processes [35] we can estimate that (basal) release probability of astrocytic exocytosis U_A is < 0.13. On the other hand, single Ca^{2+}-increases can decrease the number of vesicles in the process up to 18 ± 14% its original value [39]. In the approximation of a single exocytotic event, this sets the upper limit of U_A as high as 0.82 ± 0.14. In reality multiple releases from the same process likely occur when Ca^{2+}-dependent glutamate exocytosis from astrocytes is observed [30, 45, 110] hinting that U_A could be smaller than this limit.

Rate of vesicle recycling is dictated by the exocytosis mode. Both full-fusion of vesicles and kiss-and-run events have been observed at astrocytic processes [30] with the latter likely to occur more often [30, 40]. The most rapid recycling pathway corresponds to kiss-and-run fusion, where the rate is mainly limited by vesicle fusion with plasma membrane and subsequent pore opening [111]. Indeed reported pore-open times can be as short as 2.0 ± 0.3 ms [40]. This value corresponds to a maximal rate of vesicle recycling Ω_A of approximately Ω_A < 450 ± 80 Hz. Actual astrocytic vesicle recycling rates could be though much slower than this value if recycling could depend on timing of calcium oscillations [36]. In this latter case, Ca^{2+} oscillations at single astrocytic processes could be as slow as ~0.010 Hz [35]. Notwithstanding, for fast release events confined within 100 nm from the astrocyte plasma membrane, the re-acidification time course of a vesicle, could be as long as ~1.5 s [41] hinting an average recycling rate for astrocyte exocytosis of $\Omega_A \approx 0.6$ s^{-1}, yet slower than that measured for hippocampal neurons.

Glutamate time course. An astrocytic vesicle of 50 nm diameter (i.e. $V_v \approx 65\cdot10^{-21}$ dm^3) filled with 50 mM glutamate could release into the ESS up to (equation 12) $M = (50\cdot10^{-3}$ M$)(65\cdot10^{-21}$ dm$^3)N_A \approx 2000$ molecules, roughly one third of those estimated in synaptic vesicles [37]. The average distance from release site ℓ travelled by a glutamate molecule during the release time t_{rel} can be estimated by the Einstein-Smoluchowski relationship [52] as $\ell = \sqrt{2D^* t_{rel}}$ where D^* is the glutamate diffusion coefficient in the ESS (treated as an isotropic porous medium [112]). With $D^* \approx 0.2$ [113] and $t_{rel} \approx 1$ ms [40], it is $\ell \approx 0.63$ µm. If we take as mixing volume for the released vesicle the diffusion volume within ℓ distance from the release site, then [53] $V_e = 4\pi\zeta\,\ell^3/3$, where ζ is the volume fraction [112]. With $\zeta = 0.1$ [54] then it is $V_e \approx 10^{-16}$ dm^3 and the corresponding contribution to glutamate concentration in the ESS space given by a released vesicle is (equation 14) $G_A = (2000$ molecules$)/(N_A\cdot10^{-21}$ dm$^3) \approx 30$ µM, that is $\rho_A = V_v/V_e = (65\cdot10^{-21})/10^{-16}$ dm$^3 \approx 65\cdot10^{-5}$. Assuming an astrocytic pool of $n_v = 4$ docked vesicles, and an average release probability of $U_A = 0.5$, our estimations suggest a peak glutamate concentration immediately after exocytosis of (equation 14) $G_A = 0.5\cdot4\cdot30$ µM = 60 µM, which is indeed in the experimentally-measured range of 1 – 100 µM [114].

Glutamate transporters are likely not saturated by astrocytic glutamate [55]. This is indeed the case for our estimations too, given an effective glutamate binding affinity for the transporters between $K_u \approx 100 - 150$ mM [115]. Therefore, we approximate glutamate time course by a monoexponentially decaying term as in equation (18). Imaging of extrasynaptic glutamate dynamics in hippocampal slices hints that the decay is fast, with glutamate clearance that is mainly carried out within ~100 ms from peak concentration [56]. Indeed, under the hypothesis of sole diffusion, we can estimate that the concentration in the mixing volume V_e after $t^* = 50$ ms is [53] $G_A = \left(M/8\zeta\, N_A\left(\pi D^* t^*\right)^{3/2}\right)\exp\left(-\ell^2/4D^* t^*\right) \approx 40$ nM which is indeed close to the suggested extracellular glutamate resting concentration of ~25 nM [116]. Accordingly to equation (18) then: $G_A\left(t = t^*\right) = G_A\left(t = 0\right)\exp\left(-\Omega_c t^*\right) \quad \Rightarrow \Omega_c = 1/t^* \cdot \ln\left(G_A\left(t = 0\right)/G_A\left(t = t^*\right)\right) = 1/(50\cdot10^{-3}\text{ s})\cdot\ln(60\cdot10^{-6}\text{ M}/40\cdot10^{-9}\text{ M}) \approx 150\text{ s}^{-1}$. Alternatively the fact that extracellular glutamate concentration decays to 25 nM within 100 ms from its peak [56], leads to an estimation of $\Omega_c \approx 80$ s^{-1}. The effective clearance rate is expected to be larger for the existence of uptake [49], which is not explicitly included in our estimation.

Astrocyte modulation of synaptic release. Presynaptic depression observed following activation of presynaptic mGluRs by astrocytic glutamate could lasts from tens of seconds [70] to ~2 – 3 min [74]. Similarly group I mGluR-mediated facilitation by a single Ca^{2+} increase in an astrocytic process, may affect synaptic release at adjacent synapses for as long as ~50 – 60 s [26, 71]. Values within ~1 – 2 min have been also reported in the case of an involvement of NMDA receptors [35, 72]. Accordingly for astrocyte-mediated facilitation we can estimate the rate of recovery from astrocyte modulation Ω_G to be <0.5 – 1.2 min^{-1}.

According to our description (equation 19), the rising time of astrocyte effect depends on a multitude of factors of difficult estimation such as the glutamate time course in proximity of presynaptic receptors, the kinetics of these latter as well as their density and the intracellular mechanism that they trigger. Nonetheless, the astrocyte effect on synaptic release usually reaches its maximum within the first 1 – 5 seconds from the rise of Ca^{2+} in the astrocyte [26, 70, 72]. This observation motivated us to assume that, for the purpose of our analysis, the effect of astrocyte modulation of synaptic release could be negligible during its rise with respect to its decay. Accordingly, we consider heuristic values of O_G that could be consistent with such fast onset. In particular, a single Ca^{2+} increase could lead up to ~150 – 200% increase of release from adjacent synapses [35, 66, 71]. However evidence from early studies in vitro hints that the entity of modulation of synaptic release due to the astrocyte could virtually be any up to ~10 times the original [72].

In our model the possible maximal astrocyte-induced facilitation depends on the resting value U_0* as well as on the value of the effect parameter a. Indeed from equation (21) in order to have facilitation (i.e for $U_0^* < \alpha \leq 1$), it must be $(1-\Gamma)U_0^* + \alpha\Gamma \geq \eta U_0^*$ for $\eta > 1$ which requires $(\eta-1)U_0^*/(\alpha-U_0^*) \leq \Gamma \leq 1 \Rightarrow 1 < \eta \leq \alpha/U_0^*$. That is, for $\alpha = 1$, starting from $U_0^* = 0.5$, the astrocyte could at most increase the release probability up to two times its original values, since indeed $U_0^* \leq (2)(0.5) \leq 1$. In the opposite case of astrocyte-induced presynaptic depression, namely for $0 \leq \alpha < U_0^*$, maximal depression sets instead an upper bound for the allowed value of Γ. From equation (21) indeed it follows that $(1-\Gamma)U_0^* + \alpha\Gamma \geq \eta U_0^*$ with $0 < \eta < 1$ if and only if $\Gamma \geq (1-\eta)U_0^*/(U_0^*-\alpha)$. If we assume maximal depression and minimal facilitation to be respectively ~20% and 120% the resting U_0* value, it follows that $0.2 < \Gamma < 0.8$. Given that the peak of

astrocyte effect can be estimated as $\Gamma_{\text{peak}} = O_{\text{G}} U_{\text{A}} G_{\text{A}} / \Omega_{\text{c}}$ (equations 15, 19, 45), it follows that ~0.4 < O_{G} < 2.

References

1. Markram H, Wang Y, Tsodyks M (1998) Differential signaling via the same axon of neocortical pyramidal neurons. Proc Natl Acad Sci USA 95: 5323–5328.

2. Tsodyks MV, Markram H (1997) The neural code between neocortical pyramidal neurons depends on neurotransmitter release probability. Proc Natl Acad Sci USA 94: 719-723.

3. Zucker RS, Regehr WG (2002) Short-term synaptic plasticity. Annual Rev Physiol 64: 355-405.

4. Bollmann JH, Sakmann B, Gerard J, Borst G (2000) Calcium sensitivity of glutamate release in a calyx-type terminal. Science 289: 953-957.

5. Schneggenburger R, Neher E (2000) Intracellular calcium dependence of transmitter release rates at a fast central synapse. Nature 406: 889-893.

6. Del Castillo J, Katz B (1954) Quantal components of the end-plate potential. J Physiol 124: 560-573.

7. Fuhrmann G, Segev I, Markram H, Tsodyks M (2002) Coding of temporal information by activity-dependent synapses. J Neurophysiol 87: 140-148.

8. Tsodyks M, Pawelzik K, Markram H (1998) Neural networks with dynamic synapses. Neural Computation 10: 821-835.

9. Agulhon C, Petravicz J, McMullen AB, Sweger EJ, Minton SK, et al. (2008) What is the role of astrocyte calcium in neurophysiology? Neuron 59: 932-946.

10. Berridge MJ, Bootman MD, Roderick HL (2003) Calcium signalling: dynamics, homeostasis and remodelling. Nature Rev 4: 517-529.

11. Nimmerjahn A (2009) Astrocytes going live: advances and challenges. J Physiol 587: 1639-1647.

12. Bezprozvanny I, Watras J, Ehrlich BE (1991) Bell-shaped calcium-response curves of Ins(1,4,5)P_3- and calcium-gated channels from endoplasmic reticulum of cerebellum. Nature 351: 751-754.

13. De Pittà M, Volman V, Levine H, Pioggia G, De Rossi D, et al. (2008) Coexistence of amplitude and frequency modulations in intracellular calcium dynamics. Phys Rev E 77: 030903(R).

14. De Pittà M, Goldberg M, Volman V, Berry H, Ben-Jacob E (2009) Glutamate-dependent intracellular calcium and IP_3 oscillating and pulsating dynamics in astrocytes. J Biol Phys 35: 383-411.

15. De Pittà M, Volman V, Levine H, Ben-Jacob E (2009) Multimodal encoding in a simplified model of intracellular calcium signaling. Cogn Proc 10: 55-70.

16. Carmignoto G (2000) Reciprocal communication systems between astrocytes and neurones. Prog Neurobiol 62: 561-581.

17. Berridge MJ (1997) The AM and FM of calcium signaling. Nature 389: 759-760.

18. Cornell-Bell AH, Finkbeiner SM, Cooper MS, Smith SJ (1990) Glutamate induces calcium waves in cultured astrocytes: long-range glial signaling. Science 247: 470-473.

19. Berridge MJ, Lipp P, Bootman MD (2000) The versatility and universality of calcium signalling. Nat Rev Mol Cell Biol 1: 11-21.

20. Falcke M (2004) Reading the patterns in living cells - the physics of Ca^{2+} signaling. Adv Phys 53: 255-440.

21. Izhikevich EM (2000) Neural excitability, spiking and bursting. Int J Bif Chaos 10: 1171-1266.

22. Kuznetsov Y (1998) Elements of Applied Bifurcation Theory. New York, U.S.A.: Springer, 2nd edition.

23. Kuga N, Sasaki T, Takahara Y, Matsuki N, Ikegaya Y (2011) Large-scale calcium waves traveling through astrocytic networks *in vivo*. J Neurosci 31: 2607-2614.

24. Goldberg M, De Pittà M, Volman V, Berry H, Ben-Jacob E (2010) Nonlinear gap junctions enable long-distance propagation of pulsating calcium waves in astrocyte networks. PLoS Comput Biol 6: e1000909.

25. Scemes E, Giaume C (2006) Astrocyte calcium waves: What they are and what they do. Glia 54: 716-725.

26. Fiacco TA, McCarthy KD (2004) Intracellular astrocyte calcium waves *in situ* increase the frequency of spontaneous AMPA receptor currents in CA1 pyramidal neurons. J Neurosci 24: 722-732.

27. Parpura V, Haydon PG (2000) Physiological astrocytic calcium levels stimulate glutamate release to modulate adjacent neurons. Proc Natl Acad Sci USA 97: 8629-8634.

28. Pasti L, Zonta M, Pozzan T, Vicini S, Carmignoto G (2001) Cytosolic calcium oscillations in astrocytes may regulate exocytotic release of glutamate. J Neurosci 21: 477-484.

29. Parpura V, Zorec R (2010) Gliotransmission: exocytotic release from astrocytes. Brain Res Rev 63: 83-92.

30. Bezzi P, Gundersen V, Galbete JL, Seifert G, Steinhäuser C, et al. (2004) Astrocytes contain a vesicular compartment that is competent for regulated exocytosis of glutamate. Nat Neurosci 7: 613-620.

31. Hua X, Malarkey EB, Sunjara V, Rosenwald SE, Li WH, et al. (2004) Ca^{2+}-dependent glutamate release involves two classes of endoplasmic reticulum Ca^{2+} stores in astrocytes. J Neurosci Res 76: 86-97.

32. Verkhratsky A, V P (2010) Recent advances in (patho)physiology of astroglia. Acta Pharmacologica Sinica 31: 1044-1054.

33. Santello M, Volterra A (2009) Synaptic modulation by astrocytes via Ca^{2+}-dependent glutamate release. Neuroscience 158: 253-259.

34. Bergersen LH, Gundersen V (2009) Morphological evidence for vesicular glutamate release from astrocytes. Neuroscience 158: 260-265.

35. Jourdain P, Bergersen LH, Bhaukaurally K, Bezzi P, Santello M, Domercq M, et al. (2007) Glutamate exocytosis from astrocytes controls synaptic strength. Nature Neurosci 10: 331-339.

36. Zhang Q, Pangršic T, Kreft M, Kržan M, Li N, et al. (2004) Fusion-related release of glutamate from astrocytes. J Biol Chem 279: 12724-12733.

37. Montana V, Malarkey EB, Verderio C, Matteoli M, Parpura V (2006) Vesicular transmitter release from astrocytes. Glia 54: 700-715.

38. Stenovec M, Kreft S M Grilc, Pokotar M, Kreft ME, Pangršic T, et al. (2007) Ca^{2+}-dependent mobility of vesicles capturing anti-VGLUT1 antibodies. Exp Cell Res 313: 3809-3818.

39. Crippa D, Schenk U, Francolini M, Rosa P, Verderio C, et al. (2006) Synaptobrevin2-expressing vesicles in rat astrocytes: insights into molecular characterization, dynamics and exocytosis. J Physiol 570: 567-582.

40. Chen X, Wang L, Zhou Y, Zheng LH, Zhou Z (2005) “Kiss-and-run" glutamate secretion in cultured and freshly isolated rat hippocampal astrocytes. J Neurosci 25: 9236-9243.

41. Bowser DN, Khakh BS (2007) Two forms of single-vesicle astrocyte exocytosis imaged with total internal reflection fluorescence microscopy. Proc Natl Acad Sci USA 104: 4212-4217.

42. Marchaland J, Calì C, Voglmaier SM, Li H, Regazzi R, et al. (2008) Fast subplasma membrane Ca^{2+} transients control exo-endocytosis of synaptic-like microvesicles in astrocytes. J Neurosci 28: 9122-9132.

43. Südhof TC (2004) The synaptic vesicle cycle. Annu Rev Neurosci 27: 509-547.

44. Perea G, Araque A (2005b) Properties of synaptically evoked astrocyte calcium signal reveal synaptic information processing by astrocyte. J Neurosci 25: 2192-2203.

45. Santello M, Bezzi P, Volterra A (2011) TNFα controls glutamatergic gliotransmission in the hippocampal dentate gyrus. Neuron 69: 988-1001.

46. Diamond JS (2005) Deriving the glutamate clearance time course from transporter currents in CA1 hippocampal astrocytes: transmitter uptake gets faster during development. J Neurosci 25: 2906 – 2916.

47. Diamond JS, Jahr CE (1997) Transporters buffer synaptically released glutamate on a submillisecond time scale. J Neurosci 17: 4672– 4687.

48. Clements JD (1996) Transmitter timecourse in the synaptic cleft: its role in central synaptic function. Trends Neurosci 19: 163-171.

49. Tzingounis AV, Wadiche JI (2007) Glutamate transporters: confining runaway excitation by shaping synaptic transmission. Nature Rev 8: 935-947.

50. Aguado F, Espinosa-Parrilla JF, Carmona MA, Soriano E (2002) Neuronal activity regulates correlated network properties of spontaneous calcium transients in astrocytes *in situ*. J Neurosci 22: 9430-9444.

51. Venance L, Stella N, Glowinski J, Giaume C (1997) Mechanism involved in initiation and propagation of receptor-induced intercellular calcium signaling in cultured rat astrocytes. J Neurosci 17: 1981-1992.

52. Schikorski T, Stevens CF (1997) Quantitative ultrastructural analysis of hippocampal excitatory synapses. J Neurosci 17: 5858-5867.

53. Barbour B, Häusser M (1997) Intersynaptic diffusion of neurotransmitter. Trends Neurosci 20: 377-384.

54. Rusakov DA, Kullmann DM (1998) Extrasynaptic glutamate diffusion in the hippocampus: ultrastructural constraints, uptake, and receptor activation. J Neurosci 18: 3158-3170.

55. Diamond JS, Jahr CE (2000) Synaptically released glutamate does not overwhelm transporters on hippocampal astrocytes during high-frequency stimulation. J Neurophysiol 83: 2835-2843.

56. Okubo Y, Sekiya H, Namiki S, Sakamoto H, Iinuma S, et al. (2010) Imaging extrasynaptic glutamate dynamics in the brain. Proc Nat Acad Sci USA 107: 6526-6531.

57. Scimemi A, Tian H, Diamond JS (2009) Neuronal transporters regulate glutamate clearance, NMDA receptor activation, and synaptic plasticity in the hippocampus. J Neurosci 29: 14581-14595.

58. Danbolt NC (2001) Glutamate uptake. Progress Neurobiol 65: 1-105.

59. Südhof T (1995) The synaptic vesicle cycle: a cascade of protein-protein interactions. Nature 375: 645-653.

60. Suzuki A, Stern SA, O B, Huntely G, Wlaker RH, et al. (2011) Astrocyte-neuron lactate transport is required for long-term memory formation. Cell 144: 810-823.

61. Nedergaard M, Takano T, Hansen A (2002) Beyond the role of glutamate as a neurotransmitter. Nature Reviews Neuroscience 3: 748-755.

62. Hertz L, Zielke H (2004) Astrocytic control of glutamatergic activity: astrocytes as stars of the show. TRENDS in Neurosciences 27: 735-743.

63. Tsacopoulos M, Magistretti P (1996) Metabolic coupling between glia and neurons. The Journal of Neuroscience 16: 877-885.

64. Cruz N, Ball K, Dienel G (2007) Functional imaging of focal brain activation in conscious rats: impact of [^{14}C] glucose metabolite spreading and release. Journal of Neuroscience Research 85: 3254-3266.

65. Daikhin Y, Yudkoff M (2000) Compartmentation of brain glutamate metabolism in neurons and glia. The Journal of Nutrition 130: 1026S-1031S.

66. Bonansco C, Couve A, Perea G, Ferradas CA, Roncagliolo M, et al. (2011) Glutamate released spontaneously from astrocytes sets the threshold for synaptic plasticity. Eur J Neurosci 33: 1483-1492.

67. Stenovec M, Kreft M, Grilc S, Pangršic T, Zorec R (2008) EAAT2 density at the astrocyte plasma membrane and Ca^{2+}-regulated exocytosis. Mol Membr Biol 25: 203-215.

68. Andersson M, Hanse E (2010) Astrocytes impose postburst depression of release probability at hippocampal glutamate synapses. J Neurosci 30: 5776-5780.

69. Andersson M, Blomstrand F, Hanse E (2007) Astrocytes play a critical role in transient heterosynaptic depression in the rat hippocampal CA1 region. J Physiol 585: 843-852.

70. Araque A, Parpura V, Sanzgiri RP, Haydon PG (1998) Glutamate-dependent astrocyte modulation of synaptic transmission between cultured hippocampal neurons. Eur J Neurosci 10: 2129-2142.

71. Perea G, Araque A (2007) Astrocytes potentiate transmitter release at single hippocampal synapses. Science 317: 1083-1086.

72. Araque A, Sanzgiri RP, Parpura V, Haydon PG (1998) Calcium elevation in astrocytes causes an NMDA receptor-dependent increase in the frequency of miniature synaptic currents in cultured hippocampal neurons. J Neurosci 18: 6822-6829.

73. Pinheiro PS, Mulle C (2008) Presynaptic glutamate receptors: physiological functions and mechanisms of action. Nature Rev 9: 423-436.

74. Liu QS, Xu Q, Kang J, Nedergaard M (2004) Astrocyte activation of presynaptic metabotropic glutamate receptors modulates hippocampal inhibitory synaptic transmission. Neuron Glia Biol 1: 307-316.

75. Niswender CM, Conn PJ (2010) Metabotropic glutamate receptors: physiology, pharmacology, and disease. Annu Rev Pharmacol Toxicol 50: 295-322.

76. Corlew R, Brasier DJ, Feldman DE, Philpot BD (2010) Presynaptic NMDA receptors: newly appreciated roles in cortical synaptic function and plasticity. Neuroscientist 14: 609-625.

77. Engelman HS, MacDermott AB (2004) Presynaptic ionotropic receptors and control of transmitter release. Nature Rev 5: 135-145.

78. Cochilla AJ, Alford S (1998) Metabotropic glutamate receptor-mediated control of neurotransmitter release. Neuron 20: 1007-1016.

79. Worden MK, Bykhovskaia M, Hackett JT (1997) Facilitation at the lobster neuromuscular junction: a stimulus-dependent mobilization model. J Neurophysiol 78: 417-428.

80. Hori T, Takahashi T (2009) Mechanisms underlying short-term modulation of transmitter release by presynaptic depolarization. J Physiol 587: 2987-3000.

81. Awatramani GB, Price GD, Trussell LO (2005) Modulation of transmitter release by presynaptic resting potential and background calcium levels. Neuron 48: 109-121.

82. Volman V, Ben-Jacob E, Levine H (2007) The astrocyte as a gatekeeper of synaptic information transfer. Neur Comput 19: 303-326.

83. Haber M, Zhou L, Murai KK (2006) Cooperative astrocyte and dendritic spine dynamics at hippocampal excitatory synapses. J Neurosci 26: 8881-8891.

84. Panatier A, Theodosis DT, Mothet JP, Touquet B, Pollegioni L, et al. (2006) Glia-derived D-serine controls NMDA receptor activity and synaptic memory. Cell 125: 775-784.

85. Destexhe A, Mainen ZF, Sejnowski TJ (1994) Synthesis of models for excitable membranes, synaptic transmission and neuromodulation using a common kinetic formalism. J Comp Neurosci 1: 195–230.

86. Jahr CE, Stevens CF (1990) Voltage dependence of NMDA-activated macroscopic conductances predicted by single-channel kinetics. J Neurosci 10: 3178-3182.

87. Nadkarni S, Jung P (2007) Modeling synaptic transmission of the tripartite synapse. Phys Biol 4: 1-9.

88. Schneggenburger R, Sakaba T, Neher E (2002) Vesicle pools and short-term synaptic depression: lessons form a large synapse. Trends Neurosci 25: 206-212.

89. Katz B, Miledi R (1968) The role of calcium in neuromuscular facilitation. J Physiol 195: 481-492.

90. Debanne D, Guerineau NC, Giihwiler BH, Thompson SM (1996) Paired-pulse facilitation and depression at unitary synapses in rat hippocampus: quantal fluctuation affects subsequent release. J Physiol 491: 163-176.

91. Dittman JS, Kreitzer AC, Regehr WG (2000) Interplay between facilitation, depression, and residual calcium at three presynaptic terminals. J Neurosci 20: 1374-1385.

92. Tsodyks M (2005) Activity-dependent transmission in neocortical synapses. In: Chow CC, Gutkin B, D H, C M, Dalibard J, editors, Methods and Models in Neurophysics, Elsevier. pp. 245-266.

93. Softky W, Koch C (1993) The highly irregular firing pattern of cortical cells is inconsistent with temporal integration of random EPSPs. J Neurosci 13: 334-350.

94. Amit DJ, Tsodyks MV (1991) Quantitative study of attractor neural network retrieving at low spike rates: I. Substrate-spikes, rates and neuronal gain. Network 2: 259-273.

95. Skupin A, Falcke M (2010) Statistical analysis of calcium oscillations. Eur Phys J Special Topics 187: 231-240.

96. Takata N, Hirase H (2008) Cortical layer 1 and 2/3 astrocytes exhibit distinct calcium dynamics. PLoS ONE 3: e2525.

97. Shigetomi E, Kracun S, Sovfroniew MS, Khakh BS (2010) A genetically targeted optical sensor to monitor calcium signals in astrocyte processes. Nature Neurosci 13: 759-766.

98. Stevens CF, Wang Y (1995) Facilitation and depression at single central synapses. Neuron 14: 795-802.

99. Stevens CF, Wang Y (1994) Changes in reliability of synaptic function as a mechanism for plasticity. Nature 371: 704-707.

100. Murthy VN, Sejnowski TJ, Stevens CF (1997) Heterogeneous release properties of visualized individual hippocampal synapses. Neuron 18: 599-612.

101. Pyle JL, Kavalali ET, Piedras-Rentería ES, Tsien RW (2000) Rapid reuse of readily releasable pool vesicles at hippocampal synapses. Neuron 28: 221-231.

102. Brody DL, Yue DT (2000) Release-independent short-term synaptic depression in cultured hippocampal neurons. J Neurosci 20: 2480-2494.

103. Emptage NJ, Reid CA, Fine A (2001) Calcium stores in hippocampal synaptic boutons mediate short-term plasticity, store-operated Ca^{2+} entry, and spontaneous transmitter release. Neuron 29: 197-208.

104. Regehr WF, Delaney KR, Tank DW (1994) The role of presynaptic calcium in short-term enhancement at the hippocampal mossy fiber synapse. J Neurosci 14: 523-537.

105. Helmchen F, Borst JGG, Sakmann B (1997) Calcium-dynamics associated with a single action potential in a CNS presynaptic terminal. Biophys J 72: 1458-1471.

106. Regehr WG, Atluri PP (1995) Calcium transients in cerebellar granule cell presynaptic terminals. Biophys J 68: 2156-2170.

107. Fellin T, Pascual O, Gobbo S, Pozzan T, Haydon PG, et al. (2004) Neuronal synchrony mediated by astrocytic glutamate through activation of extrasynaptic NMDA receptors. Neuron 43: 729-743.

108. Pasti L, Volterra A, Pozzan T, Carmignoto G (1997) Intracellular calcium oscillations in astrocytes: a highly plastic, bidirectional form of communication between neurons and astrocytes *in situ*. J Neurosci 17: 7817-7830.

109. Anlauf E, Derouiche A (2005) Astrocytic exocytosis vesicles and glutamate: a high-resolution immunofluorescence study. Glia 49: 96-106.

110. Domercq M, Brambilla L, Pilati E, Marchaland J, Volterra A, et al. (2006) P2Y1 receptor-evoked glutamate exocytosis from astrocytes: control by tumor necrosis factor-α and prostaglandins. J Biol Chem 281: 30684-30696.

111. Valtorta F, Meldolesi J, Fesce R (2001) Synaptic vesicles: is kissing a matter of competence? Trends Cell Biol 11: 324-328.

112. Nicholson C, Phillips JM (1981) Ion diffusion modified by tortuosity and volume fraction in the extracellular microenvironment of the rat cerebellum. J Physiol 321: 225-257.

113. Nielsen TA, DiGregorio DA, Silver RA (2004) Modulation of glutamate mobility reveals the mechanism underlying slow-rising AMPAR EPSCs and the diffusion coefficient in the synaptic cleft. Neuron 42: 757-771.

114. Innocenti B, Parpura V, Haydon PG (2000) Imaging extracellular waves of glutamate during calcium signaling in cultured astrocytes. J Neurosci 20: 1800-1808.

115. Bergles DE, Tzingounis AV, Jahr CE (2002) Comparison of coupled and uncoupled currents during glutamate uptake by GLT-1 transporters. J Neurosci 22: 10153-10162.

116. Herman MA, Jahr CE (2007) Extracellular glutamate concentration in hippocampal slice. J Neurosci 27: 9736-9741.
